# What Influences Language Impairment in Bilingual Aphasia? A Meta-Analytic Review

**DOI:** 10.3389/fpsyg.2019.00445

**Published:** 2019-04-04

**Authors:** Ekaterina Kuzmina, Mira Goral, Monica Norvik, Brendan S. Weekes

**Affiliations:** ^1^Faculty of Humanities, Center for Multilingualism in Society Across the Lifespan, University of Oslo, Oslo, Norway; ^2^The Graduate Center and Lehman College, City University of New York, New York, NY, United States; ^3^Department of Speech and Language Disorders, Statped, Oslo, Norway; ^4^Department of Language and Literature, Norwegian University of Science and Technology, Trondheim, Norway; ^5^Melbourne Graduate School of Education, University of Melbourne, Melbourne, VIC, Australia; ^6^Laboratory for Communication Science, Faculty of Education, The University of Hong Kong, Pokfulam, Hong Kong; ^7^State Key Laboratory for Brain and Cognitive Sciences, The University of Hong Kong, Pokfulam, Hong Kong

**Keywords:** bilingual aphasia, stroke, linguistic similarity, AoA, premorbid proficiency, language use, meta-analysis

## Abstract

Patterns of language impairment in multilingual speakers with post-stroke aphasia are diverse: in some cases the language deficits are parallel, that is, all languages are impaired relatively equally, whereas in other cases deficits are differential, that is, one language is more impaired than the other(s). This diversity stems from the intricate structure of the multilingual language system, which is shaped by a complex interplay of influencing factors, such as age of language acquisition, frequency of language use, premorbid proficiency, and linguistic similarity between one's languages. Previous theoretical reviews and empirical studies shed some light on these factors, however no clear answers have been provided. The goals of this review were to provide a timely update on the increasing number of reported cases in the last decade and to offer a systematic analysis of the potentially influencing variables. One hundred and thirty cases from 65 studies were included in the present systematic review and effect sizes from 119 cases were used in the meta-analysis. Our analysis revealed better performance in L1 compared to L2 in the whole sample of bilingual speakers with post-stroke aphasia. However, the magnitude of this difference was influenced by whether L2 was learned early in childhood or later: those who learned L2 before 7 years of age showed comparable performance in both of their languages contrary to the bilinguals who learned L2 after 7 years of age and showed better performance in L1 compared to L2. These robust findings were moderated mildly by premorbid proficiency and frequency of use. Finally, linguistic similarity did not appear to influence the magnitude of the difference in performance between L1 and L2. Our findings from the early bilingual subgroup were in line with the previous reviews which included mostly balanced early bilinguals performing comparably in both languages. Our findings from the late bilingual subgroup stressed the primacy of L1 and the importance of age of L2 learning. In addition, the evidence from the present review provides support for theories emphasizing the role of premorbid proficiency and language use in language impairment patterns in bilingual aphasia.

## Introduction

Aphasia describes a multitude of acquired language impairment resulting from brain injury, most often but not exclusively following a stroke. Bilinguals are individuals who use more than one language on a regular basis (Grosjean, 2013). Reports of individuals with bilingual aphasia have emerged as an important constraint on theories of the neurobiology of language (Gollan and Kroll, 2001; Ullman, 2001; Abutalebi et al., 2009; Miozzo et al., 2010; Weekes, 2010). Studies of bilingual aphasia began with anecdotal case studies reported by Ribot (1882) and Pitres (1895/1983). However, the wider theoretical implications of these cases are only more obvious today with the advent of sophisticated models of bilingual language processing. Two enduring questions in the field are whether a first-acquired language (L1) is less vulnerable to brain damage compared with later-learned languages (L2), and whether a language that is used more often premorbidly can be privileged after injury. Ribot's law holds that earlier acquired memories (including linguistic) are more resistant to brain damage whereas Pitres' law assumes that the premorbidly dominant language will be less vulnerable, independent of the age of acquisition (AoA) of that language. A related question is whether the cognitive and neural representations for L1 and L2 are shared or depend on different cognitive and neural mechanisms (e.g., Chee et al., 1999, 2000; Abutalebi et al., 2001; Ullman, 2001; Green, 2003; Perani and Abutalebi, 2005; Giussani et al., 2007). In our view, answers to these questions can be revealing for theories of the neurobiology of language (e.g., Libben, 2017) as well as for the design of intervention for language impairments in multilingual speakers in a variety of contexts, including immigrants and refugees across the globe (Pot et al., 2018).

The evidence emerging from studies of bilingual individuals who are recovering language function after a stroke shows that both early acquisition and premorbid language dominance contribute to language recovery and should constrain therapy (Lorenzen and Murray, 2008; Faroqi-Shah et al., 2010; Knoph, 2013; Conner et al., 2018). In many instances, equivalent patterns of aphasia in all languages spoken premorbidly are assumed, an assumption that implies shared cognitive and neural representations for these languages. The shared bilingual neural substrate (SBNS) hypothesis specifically assumes that bilingual speakers who acquire L2 early in life have a common neural network with shared lexical-semantic and syntactic representations from each language in the brain (Miozzo et al., 2010; Costa et al., 2012; Nadeau, 2019). This assumption is compatible with cognitive neuropsychological models of typical bilingual language processing (Gollan and Kroll, 2001; Faroqi-Shah and Waked, 2010) and with the view that linguistic differences between languages spoken premorbidly do not matter. One prediction from these accounts is that brain damage from stroke will result in equivalent impairment for bilingual speakers in any two languages spoken premorbidly (Paradis, 2004; Weekes, 2010).

Methodological limitations in the sampling of multilingual people with aphasia reported in previous reviews, as detailed below, and the generally accepted view that L2 processing is moderated by AoA (for a review see Abutalebi, 2008), lead us to conjecture that language status (L1 vs. L2) would be a significant predictor of language impairment after stroke for bilingual speakers. According to the convergence hypothesis proposed by Green (2003), which is consistent with the SBNS, dissociations observed in bilingual speakers between L1 and L2 could reflect greater recruitment of cognitive resources assumed to be necessary to process an explicitly learned language (L2), rather than differential neuronal representations (see Chee et al., 1999, 2000; Ullman, 2001). Furthermore, the dominance of language use in the linguistic environment of a person with aphasia will have an impact upon the patterns of aphasia after stroke, according to Pitres' law (Goral et al., 2012; see also Gollan et al., 2015). Therefore, there is merit to explore the roles that AoA, premorbid language proficiency and use, as well as language similarity have on performance in bilingual speakers after a stroke.

The goal of the present meta-analysis is thus to examine what constrains language impairment following stroke in multilingual speakers, and specifically, to investigate whether AoA, premorbid language proficiency, use and exposure, as well as linguistic similarity between spoken languages determine reported patterns of aphasia in L1 and L2.

## Background

Decades of research show that language difficulties associated with aphasia are highly selective and can affect only one language modality (e.g., comprehension vs. production) or linguistic aspect (e.g., syntactic processing). Many persons living with aphasia are multilingual (Roberts and Kiran, 2007; Ansaldo and Saidi, 2014). When a multilingual speaker has aphasia following a stroke, the languages spoken premorbidly may show comparable or differential patterns of impairment (Paradis, 2004; Weekes, 2010). Differential patterns may manifest as greater impairment in one language compared to another, or as differences in the characteristics of aphasia. The reasons for differential impairments are less certain. Theories of differential language processing and of impaired mechanisms of language control have been put forward to account for the patterns observed (e.g., Ullman, 2001; Abutalebi and Green, 2008). Furthermore, research shows that AoA, premorbid language proficiency, use and exposure, as well as linguistic similarity between spoken languages influence patterns of differential impairment observed in multilingual aphasia (e.g., Fabbro, 2001; Paradis, 2001, 2004; Lorenzen and Murray, 2008; Goral et al., 2012, 2013).

## Previous Reviews and Studies

Previous reviews asked whether multilingual speakers with aphasia evidence comparable levels of language impairment in all languages spoken premorbidly. For example, Albert and Obler (1978) reviewed 108 cases of multilingual aphasia and found comparable distributions of parallel and non-parallel impairment among those who were early “compound” bilinguals and those who learned their L2 later in life. Their review demonstrated no dominant pattern of results supporting only Ribot's law or only Pitres' law, and that variables, such as age, age of language acquisition, and education influenced the outcome.

Paradis (2001) reviewed 132 cases published in the period from 1990 to 1999 and found that 81 cases (61%) showed parallel impairment in both languages [“when both (or all) languages are similarly impaired and restored at the same rate,” p. 70], 24 (18%) had differential impairment (“impairment is of different degree in each language relative to premorbid mastery,” p. 70), and the remainder was shared by 12 cases (9%) with blended impairment (“when patients systematically mix or blend features of their languages at any all levels of linguistic structure,” p. 70), 9 cases (7%) with selective impairment (“when patients do not regain the use of one or more of their languages,” p. 70), and 6 cases (5%) with successive impairment (“when one language does not begin to reappear until another has been maximally recovered,” p. 70). It is important to notice that the distribution of the impairment pattern percentages in this review was highly influenced by the two relatively large group studies from which 99 cases (75%) were taken. In the first study by Junqué et al. (1995), impairment patterns of 50 early equally proficient Catalan-Spanish aphasic bilinguals with unequal premorbid frequency of language use were reported. In the second one by Vilariño et al. (1997), comparable impairment corresponding to premorbid proficiency was reported as the most frequent pattern based on the assessment of 49 early Galician-Spanish bilinguals with aphasia. Thus, the majority (75%) of the 132 cases included in the Paradis (2001) review were early, relatively balanced bilingual speakers of two closely related languages. Additionally, it is important to note that, firstly, the reviewed cases were of varying etiology (e.g., stroke, tumor), secondly, it was not systematically specified what language performance measures were used for assessment, and, finally, the criteria for making a decision about the comparability of impairments in both languages were not explicitly defined.

Fabbro (2001) used the Bilingual Aphasia Test (BAT, Paradis and Libben, 1987) to assess 20 Friulian-Italian early (L2 learned between 5 and 7 year) bilingual speakers with aphasia, who premorbidly used both languages on a regular basis and had a stroke from 1 to 96 months before the assessment. Premorbid proficiency of the participants was not directly assessed, the author allegedly assumed that all participants were equally proficient in both languages. According to the author's interpretation of the results, 13 participants (65%) had comparable impairments in both languages, 4 participants (20%) performed significantly worse in L2, and 3 participants (15%) performed significantly worse in L1 (We note that for one of these three last participants, *p*-value was 0.07 indicating the absence of significant differences.) The researcher concluded that these percentages were in line with the previous review by Paradis (2001). However, Fabbro's study included early balanced (having comparable premorbid proficiency in both languages) bilinguals only. Moreover, decisions about the difference between performance in L1 and L2 were made based on running significance tests separately for each of 20 participants, subjecting the results to a Type I error (overestimation of significant difference).

Other reviews have identified additional relevant variables. In their review, Lorenzen and Murray (2008) suggested that language similarity (proportion of cognates shared) was a significant constraint on language recovery in bilingual speakers after stroke. Ansaldo et al. (2008) argued, as others have earlier (see Paradis, 2004), that motivation impacts on recovery. Overall, extant reviews suggest that equivalent patterns of language impairment in bilingual aphasia are more common, but a large minority of cases do show differential or selective patterns of impairment. These reviews also highlight the variables that predict recovery in post-stroke bilingual aphasia: AoA, language proficiency, language use, and linguistic similarity.

## Age of Language Acquisition

AoA refers to the age at which people learn language. It has long been argued that words acquired at an early age are the ones that are most preserved in aphasia (Rochford and Williams, 1965; Brysbaert and Ellis, 2016; Bakhtiar et al., 2017) although experimental evidence has been mixed, with some later-learned words found to be more easily retrieved in some cases of aphasia (e.g., Goral et al., 2013). Much research has studied the question of whether languages that are learned later in childhood or in adulthood, as compared to early acquired first language(s), are organized or processed by different neural mechanisms (this discussion is beyond the scope of this paper but see Ullman, 2001; Birdsong, 2006; Abutalebi and Green, 2007 among others). In the literature on bilingual aphasia, most reports highlight the age in which the languages spoken were first acquired (e.g., for all 130 cases included in this review).

Whereas this question preoccupied early reviews (Albert and Obler, 1978; Junqué et al., 1995), relatively few recent studies of bilingual aphasia explicitly addressed the role of age of language learning on language impairment. Among those who did, Tschirren et al. (2011) found no evidence of differential performance in their late bilinguals, suggesting that late learning of L2 is not always an impediment after a stroke. They did, however, find that AoA had an impact on syntactic processing in the two languages. Other studies have found lower performance in a later-learned language than earlier-acquired ones despite pre-aphasia high levels of proficiency (e.g., Goral et al., 2006; Kiran and Iakupova, 2011; Kurland and Falcon, 2011). However, as noted by these authors, levels of premorbid proficiency in all languages spoken is difficult to assess. We address this issue next.

## Language Proficiency

To determine language impairments in multilingual speakers with aphasia one needs to estimate their premorbid proficiency in these languages. However, premorbid language proficiency can only be estimated indirectly primarily via subjective ratings. Several questionnaires have been developed to elicit such ratings (Paradis and Libben, 1987; Muñoz and Marquardt, 2003; Kiran et al., 2010), but it has been demonstrated that self-ratings are not completely consistent with objective measures (Tomoschuk et al., 2018). Therefore, having no access to objective measures of proficiency prior to brain damage is a limitation. Recent studies have examined the notion that levels of language proficiency are highly related to levels of language exposure and use, suggesting that understanding patterns of language use could augment decisions about degree of language proficiency when only subjective measures are available (Kiran and Tuchtenhagen, 2005).

## Language Use and Exposure

Multilinguals are likely to attain and maintain high proficiency in languages they use regularly and frequently, especially if these languages are spoken in their living environment. When one language or more is not used, it could undergo processes of reduced activation and attrition (Köpke et al., 2007). Furthermore, living in the environment where one language is predominantly used can lead to inhibition of less used languages in immersed L2 learners (Linck et al., 2009). Thus, it can be assumed that the linguistic context at the time of the stroke can contribute to better perseverance and/or recovery of the relevant language in people with aphasia. In several studies, findings pointed to the role of the linguistic environment on the response to therapy (Goral et al., 2012, 2013), which are consistent with the importance of language context in addition to age of acquisition and language proficiency.

## Linguistic Similarity

Differential performance between languages that are linguistically similar (e.g., Friulian and Italian) may be surprising but is in fact reported. Less expected is equivalent patterns of aphasia in languages that are linguistically different (e.g., Chinese and English). One reason for these reports is that manifestations of aphasia syndromes (e.g., agrammatism) are not possible in some languages and therefore similar patterns in linguistically different languages will not be observed (Weekes, 2010). Similarly, it is possible that different constraints that characterize linguistic systems (e.g., the depth of an orthography or complexity of morphology) will produce differential patterns of recovery (see Menn and Obler, 1990; Paradis, 2001; Weekes, 2005, 2012). When languages are similar in terms of their cognates (words that have a similar meaning and form in different languages), for example, Spanish and Catalan, linguistic distance is relatively small compared to languages without cognates, for example, Spanish and Mandarin. Despite this, selective impairments can be seen between linguistically similar languages.

Linguistic similarity has been associated with recovery in bilingual aphasia (Kohnert, 2004; Kendall et al., 2015). However, competition between cognates has also been observed (e.g., Kurland and Falcon, 2011). Linguistic similarity has been considered when testing differential impairment across languages. For example, Roberts and Deslauriers (1999) found that a group of 15 early balanced French-English bilinguals with aphasia were more accurate at naming pictures representing cognates compared to noncognates. As well, Goral et al. (2010) found cross-language effects from linguistic structures that were similar across languages but not for aspects that were different between languages of a trilingual speaker with aphasia. Similarly, Fabbro (2001) reported the most common error among Friulian-Italian bilinguals with aphasia when producing Friulian was pronoun omission, which is acceptable in many instances in Italian but ungrammatical in Friulian.

We note that the concept of linguistic similarity has been ill-defined in the literature. In the study of second or third language learning, one approach [Typological Primacy Model (TPM); Rothman, 2015] seeks to define language typology based on structural similarities and differences, rather than on the basis of language families and historical linguistics. In studies of bilingual language processing language similarities at the lexical level has been discussed with respect to the concept of cognates (e.g., Schepens et al., 2013). In most papers on bilingual aphasia no formal definition is offered (e.g., Ansaldo and Saidi, 2014).

## Clinical Relevance

Whether the first language of bilingual speakers who acquire aphasia is more likely to be better preserved, and the identification of the influencing variables that may moderate this outcome is not only interesting theoretically but is also critical to clinical practices. Language and communication assessment in aphasia may not reveal an accurate picture unless the individuals are assessed in all their languages and unless detailed information about their language history and use is obtained. Moreover, decisions about the language in which intervention is best conducted could be informed by evidence about relative degrees of impairments in the languages of the person with aphasia. There is a growing body of treatment studies that examine the effectiveness of intervention in aphasia depending on the language in which the treatment is delivered. Current findings are equivocal regarding the variables (premorbid proficiency, AoA, language use) that affect therapy outcome and cross-language generalization (e.g., Goral, 2012; Kiran et al., 2013a,b; Nadeau, 2019).

## Present Study

Aphasia is multidimensional and rarely presents as a pure syndrome in neurology (Caramazza, 1984; Caramazza and McCloskey, 1988; Nickels et al., 2011). Studies of bilingual speakers with aphasia—who are necessarily idiosyncratic in their language background—are ipso facto unique. It is therefore hardly surprising to find a majority of research in bilingual aphasia are case studies. Criticisms of case reports are longstanding, plentiful and still topical (Shallice, 1979; Caplan, 1988; McCloskey and Caramazza, 1988; Coltheart, 2017) and are not limited to the field of aphasia and have been usurped by the so called case-series approach (Schwartz and Dell, 2010; Lambon Ralph et al., 2011; Rapp, 2011). The defining quality of the case-series is the capacity to use patterns of covariance to understand underlying cognitive mechanisms, including key elements: a reasonable sample size suitable for identifying complex trends in idiosyncratic data; administration of a common set of cognitive tests; and open criteria for defining a sample motivated by theoretical questions or clinical and neuroanatomical criteria (Rapp, 2011). The single case is commonly associated with the universality assumption that characterizes “orthodox” cognitive neuropsychology (Caramazza, 1986), while the case-series approach is prima facie more compatible with the assumptions of “population thinking” (Bub, 2011). As Rapp (2011) notes despite the increase in “population thinking,” little work has been done in aphasia to understand the extent and nature of individual variability with regard to the types of cognitive mechanisms commonly investigated in cognitive psychology and neuroscience.

In this meta-analysis we attempt to honor the variability presented in the case studies and the case-series, and at the same time extract patterns that transcend the variability and allow us to generalize from the existing literature. Given the theoretical questions raised about the neurobiology of language and the above-mentioned reports about potential predictors of impairments in aphasia in bilingual speakers, we aimed to answer the five following research questions:

1. Do bilingual speakers with post-stroke aphasia show a difference in performance between the language acquired first (L1) and the later learned language (L2)?
2. Are the possible differences between L1 and L2 of different magnitude between early bilinguals and late bilinguals? Does AoA as a continuous variable moderate the outcomes in the early and late bilingual subgroups separately?
3. Does premorbid language proficiency moderate the possible differences between L1 and L2?
4. Does frequency of language use moderate the possible differences between L1 and L2?
5. Does linguistic similarity between the languages spoken by the bilingual moderate the possible differences between L1 and L2?

## Methods

### Literature Search

The following electronic databases were searched: PubMed, Science Direct, PsycINFO, CINAHL, TAYLOR, and FRANCIS Online. Five construct-related search terms (multilingual, bilingual, trilingual, quadrilingual, polyglot) and seven population-related search terms (aphasia, language disorder, language impairment, anomia, stroke, vascular, hemorrhage) were used. The search was limited to peer-reviewed papers published in the period from 2000 until 2018 and written in English. The search strings adapted for each database are reported in the Supplementary Material. First, titles of search hits were screened to define the relevance of a study to the review. Second, abstracts and method sections of results were screened for matching inclusion criteria.

### Inclusion and Exclusion Criteria

Papers reporting behavioral accuracy data on language performance of multilingual persons with post-stroke aphasia were included for complete screening.

Two inclusion criteria related to participants were used. The first criterion was presence of aphasia resulting from a single cerebrovascular accident. Participants with aphasia of other etiologies (e.g., tumor, head injury, dementia) were excluded. The second criterion was the bilingual or multilingual status of participants. The categorization of participants as bilinguals or multilinguals by an author was used to decide whether to include participants into the review. Although variation in the definitions of bilingualism/multilingualism used by different authors can be assumed, all of the included participants could be described as persons who used more than one language to communicate on a regular basis in everyday life before the stroke. This was done to ensure that a participant had at least sufficient proficiency for everyday conversation prior to their stroke (B1 level according to the Common European Framework of Reference for Languages). Thus, the operational definition of bilingualism/multilingualism was primarily based on a criterion of premorbid language use (Grosjean, 1982). For participants whose performance was reported in several papers, information was taken from all of the papers, if the assessment time was equivalent. When the same person was described in multiple papers at different data points, the earliest performance was coded. Five studies reported data on more than two languages of the participants. For all of these cases, performance in L1 and the most frequently used language were extracted and analyzed. If several L2s were equally used, the earlier acquired language was chosen for the analysis.

**Figure 1:**
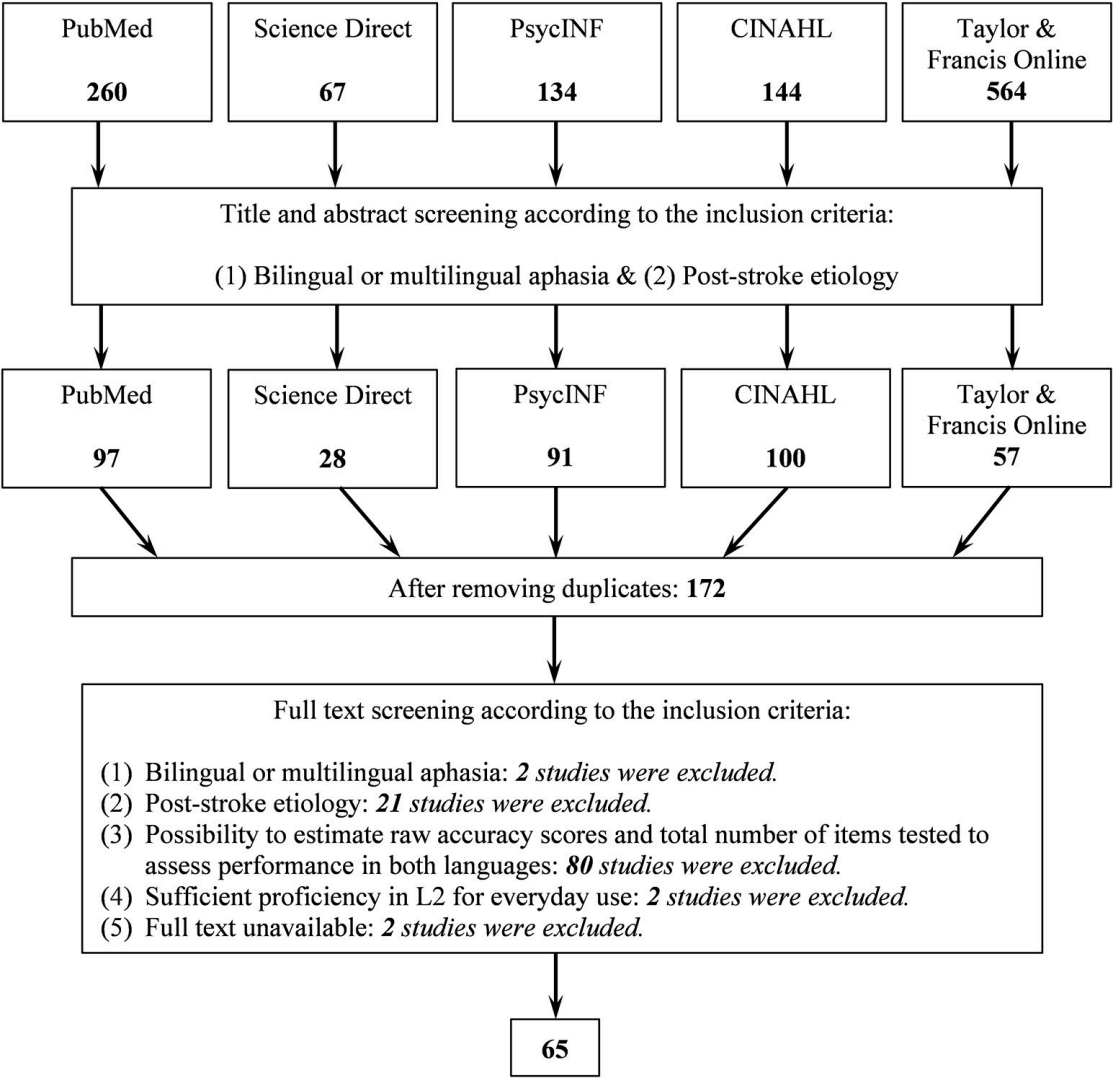
Flowchart of the search process. Numbers show how many studies were included at each stage.

Three inclusion criteria related to tests were also used. The first criterion was that a test should directly measure language performance (e.g., auditory syntactic comprehension, picture naming, reading aloud). Studies reporting performance only on tests indirectly measuring language performance (e.g., Color-Word Stroop) were not included. The second criterion was that reported performance was shown as correct responses out of the total number of tested items in various language tasks. Cases where accuracy was reported in percentages in a way that the total number of tested items in the task could not be estimated were excluded. Those studies where the total number of items used in the test was not reported, but a published test had this information (e.g., the Bilingual Aphasia Test) were included. The third criterion was the reported performance (accuracy and total number of items in the task) included data from more than one language. Cases were performance in only one language was reported were not included. After screening, 65 studies were included in the final dataset. Figure 1 shows the details of the literature search and screening process with resulting number of studies.

### Data Coding

Cases from the finally selected studies were coded according to the three study-related variables (first author or the first two authors, year of publication, first five words of the title), seven clinico-demographic case-related variables (gender, age in years at the time of assessment, years of education, month post onset at the assessment, type and severity of aphasia, lesion side), four language background variables (age of L2 acquisition, premorbid language proficiency, language use, linguistic similarity between languages), three test-related variables (test name, testing paradigm, language modality assessed by the test), and four language performance variables (numbers of items correctly performed in L1 in a specific test, total number of items in the test used to assess L1, number of items correctly performed in L2 in a specific test, total number of items in the test used to assess L2).

Coding of several above mentioned variables requires elaboration. Age of L2 acquisition was coded either as a number if it was directly reported as such, or as a time period (i.e., early childhood, primary school, later than early childhood, high school, early adulthood, adulthood) if it was directly reported or could be inferred from case descriptions. Based on the age of L2 acquisition variable, we created an adjusted variable, where we transformed categorical labels into numbers according to the following criteria: early childhood = 3 year, primary school = 7 year, later than early childhood = 10 year, high school = 14 year, early adulthood = 20 year, adulthood = 25 year. This adjustment allowed us to perform the moderator analysis treating AoA as a continuous variable.

Language proficiency was coded using three levels, namely “higher in L1,” “equal,” “higher in L2” proficiency, based on the information from case descriptions. Language use was coded based on the information from the cases using also three levels, namely “more in L1,” “equal,” “more in L2” use. To assist the coding of the language use variable, we additionally coded the following variables: language used (1) to communicate with parents, (2) with other relatives, (3) with a partner, (4) with children, (5) with friends, (6) in school as an instruction language, (7) in further education as an instruction language, (8) as a subject of formal language classes, (9) at work, (10) for reading, (11) for writing, (12) to watch TV and listen to the radio, (13) based on a self-report, (14) for therapy, (15) in the environment as an official language. These variables were used to facilitate the decision on the language use variable.

The linguistic similarity variable was coded based on how far languages are located from each other in the language family classification in two ways (rather than using for instance the TPM Rothman, 2015, which is less feasible for a meta-analysis of this scope). Firstly, language pairs from different families (e.g., English is from Indo-European family and Chinese is from Sino-Tibetan family) were coded with the level “different,” whereas all other pairs represented the “similar” level. Secondly, to make the coding of the linguistic similarity variable more precise, the three-level coding was applied: (1) language pairs from different families were coded “different” (e.g., English and Chinese), (2) language pairs which shared only the same family were coded “close” (e.g., German and Spanish), and finally, (3) language pairs which shared more than the same family were coded “very close” (i.e., English-Norwegian, Cantonese-Mandarin, Spanish-Catalan, Afrikaans-English, Malayalam-Kannada, English-Dutch, Yiddish-English, Balochi- Persian, Spanish-Italian, Italian-French, Kurdish-Persian, Spanish-French, Galician-Spanish).

Performance scores were recorded separately for each test (e.g., object naming, reading aloud words, syntactic auditory comprehension). Table 1 represents the variety of the tests included in the analysis. For tests without a defined maximum score from the spontaneous and semi-spontaneous production testing paradigm, numbers of correct information units, and corresponding total numbers of units were used as measures.

**Table 1:** Summary of the included modalities, testing paradigms, and tests.

**AUDITORY COMPREHENSION MODALITY**
**1. Commands and Yes/No questions:** *AAT:* Token test; *BAT:* Simple and semi-complex commands, Complex commands; *ILAT:* Commands; *MAST:* Yes/No questions; *WAB:* Commands, Yes/No questions.
**2. Story or paragraph:** *BAT:* Paragraph; *WAB:* Complex ideational material.
**3. Auditory input to picture matching:** *Authors' tasks:* Pointing - words, Pointing - sentences; *BAT*: Pointing - words, Auditory discrimination, Pointing - sentences; *BPVS:* Pointing - sentences; *CNL LSBA:* Lexical discrimination, Pointing - words; *ILAT:* Pointing - words; *PPVT:* Pointing - words; *PAL:* Pointing - words; *WAB:* Auditory discrimination, Pointing - words.
**4. Syntactic grammaticality judgment:** *Authors' task:* Grammaticality judgment; *BAT:* Grammaticality judgment; *CNL LSBA:* Grammaticality judgment.
**5. Lexical decision:** *Authors' task:* Lexical decision; *BAT:* Lexical decision; *CNL LSBA:* Lexical decision.
**6. Semantic relationship judgment:** *BAT:* Semantic acceptability, Semantic categories, Synonyms and antonyms, Semantic judgments.
**7. Other measures:** *Authors' task:* Auditory discrimination; *BAT:* Auditory comprehension, Auditory comprehension (pointing, semi-complex and complex commands), Auditory comprehension (pointing, semi-complex and complex commands, auditory discrimination), Sentence semantic violation judgment; *CAT:* Comprehension - words, sentences, and paragraph; *ILAT:* Phonemic analysis; *WAB:* Auditory comprehension, Auditory comprehension (Yes/No questions, word recognition, sequential commands).
**ORAL PRODUCTION MODALITY**
**8. Confrontation picture naming:** *AAT:* Naming; *Authors' task:* Naming - actions, Naming - objects; *BAT:* Naming - objects; *BNT:* Naming - objects; *CNL LSBA*: Naming - actions, Naming - objects; *ILAT:* Naming; *OANB:* Naming - objects and actions; *SWB:* Naming - objects; *WAB:* Naming; *Greek Action Test*: Naming - actions; *PALPA:* Naming.
**9. Repetition:** *AAT:* Repetition; *Authors' task:* Repetition - words and nonwords; *BAT:* Repetition - words and nonwords, Repetition - sentences; *CAT:* Repetition; *CNL LSBA:* Repetition - words, nonwords, and sentences; *PALPA:* Repetition; *WAB:* Repetition.
**10. Responsive speech and sentence completion:** *Authors' task:* Sentence completion; *CNL LSBA:* Sentence completion; *WAB:* Responsive speech, Sentence completion.
**11. Sentence construction:** *BAT:* Sentence construction; *CNL LSBA:* Sentence construction.
**12. Semantic opposites:** *BAT:* Semantic opposites.
**13. Producing morphological derivatives:** *BAT:* Morphological opposites; *CNL LSBA:* Morphological production, verb tense.
**14. Spontaneous and semi-spontaneous production:** *AAT:* Spontaneous production; *Authors' task:* Personal narrative in CIU (correct information units), Picture description in composite rubric scores; *BAT:* Picture description, Spontaneous speech; *BDAE:* Picture description; *CAT:* Picture description; *SPPA:* Picture description; *WAB:* Picture description, Narrative production.
**OTHER MODALITIES**
**15. Reading aloud:** *Authors' task:* Reading aloud - words and no words; *BAT:* Reading aloud - words, Reading aloud - sentences; *CAT:* Reading aloud; *WAB:* Reading aloud; *CNL LSBA:* Reading aloud - words, Reading aloud - nonwords.
**16. Written comprehension:** *Authors' task:* Visual lexical decision, Written word to picture matching; *BAT:* Reading comprehension - words, Reading comprehension - sentences, Reading comprehension - paragraph; *CAT:* Reading comprehension - words and sentences; *ILAT:* Reading comprehension - paragraph.
**17. Written production:** *AAT:* Writing; *BAT:* Copying, Writing to dictation - words, Writing to dictation - sentences; *CAT:* Copying; *CNL LSBA:* Writing to dictation; *PALPA:* Writing; *WAB:* Writing.
**UNCATEGORIZED MEASURES**
**18.** *AAT:* General comprehension; *BAT:* Semantics (semantic categories, synonyms and antonyms, semantic acceptability, semantic opposites); General comprehension, Total score; *MAST:* Total score.

*AAT, Aachen Aphasia Test; BAT, Bilingual Aphasia Test; BDAE, Boston Diagnostic Aphasia Evaluation; BNT, Boston Naming Task; BPVS, British Picture Vocabulary Scale; CAT, Comprehensive Aphasia Test; CNL LSBA, Cognitive Neuropsychology Laboratory Language Screening Battery Action; ILAT, Israeli Loewenstein Aphasia Test; MAST, Mississippi Aphasia Screening Test; OANB, Object and Action Naming Battery; PAL, Psycholinguistic Assessment of Language; PALPA, Psycholinguistic Assessments of Language Processing in Aphasia; PPVT, Peabody Picture Vocabulary Test; SPPA, Sentence Production Program for Aphasia; SWB, Snodgrass and Vanderwart Battery; WAB, Western Aphasia Battery*.

### Dealing With Heterogeneity in Measures

In the majority of the studies, participants were assessed with multiple tests. Firstly, scores from the individual tests were pooled together based on 18 testing paradigms summarized in Table 1. Then, scores from testing paradigms were pooled together based on the two main language modalities, namely auditory comprehension and oral production. Thus, seven testing paradigms (i.e., auditory comprehension of commands and yes/no questions, auditory comprehension of a story or paragraph, auditory based pointing, auditory syntactic grammaticality judgment, auditory lexical decision, auditory semantic relationship judgment, and other scores including sums of auditory comprehension related tests) were pooled together into auditory comprehension scores. Seven other testing paradigms (i.e., confrontation picture naming, repetition, responsive speech and sentence completion, sentence construction, oral production of semantic opposites, oral production of morphological derivatives, spontaneous and semi-spontaneous production) were pooled together into oral production scores. The other modalities category included three testing paradigms: reading aloud, written comprehension, and written production. Other tests which could not be categorized under these three modalities were kept separately. Finally, scores from auditory comprehension, oral production, other modalities, and uncategorized measures were pooled together to get the overall performance scores.

We performed correlational analysis to explore relationships between scores accumulated into the testing paradigms, scores pooled into the two main language modalities (auditory comprehension and oral production), and scores pooled into overall performance category (see Table 2). Spearman's correlation coefficients between the overall performance, total auditory comprehension, and total oral production scores varied from *rs* = 0.57 to rs = 0.94 suggesting moderate to very strong relationships. For the rest of the correlations, 79 (73%) varied from *rs* = 0.61 to *rs* = 0.95 indicating strong and very strong relationships, 21 (19%) varied from *rs* = 0.40 to *rs* = 0.59 indicating moderate relationships, and 8 (7%) correlation coefficients varied from *rs* = 0.30 to *rs* = 0.39 indicating weak relationships. Based on the results of this analysis, we concluded that the procedure of pooling scores from various test paradigms into the auditory comprehension and oral production modalities, as well as later pooling all available language performance scores into the overall performance category was justified.

**Table 2:** Correlations between the testing paradigms and the three types of scores used in the analysis.

**Testing paradigms**	**L1**						**L2**					
	**Auditory comprehension total**		**Oral production total**		**Overall performance**		**Auditory comprehension total**		**Oral production total**		**Overall performance**	
	***rs***	***n***	***rs***	***n***	***rs***	***n***	***rs***	***n***	***rs***	***n***	***rs***	***n***
**AUDITORY COMPREHENSION MODALITY**												
Commands and Yes/No questions	0.86^**^	48	0.59^**^	48	0.72^**^	48	0.88^**^	48	0.60^**^	48	0.75^**^	48
Story or paragraph	0.77^**^	23	0.61^**^	23	0.68^**^	23	0.82^**^	23	0.75^**^	23	0.83^**^	23
Auditory input to picture matching	0.83^**^	62	0.55^**^	54	0.74^**^	62	0.84^**^	62	0.62^**^	54	0.74^**^	62
Syntactic grammaticality judgment	0.85^**^	36	0.56^**^	27	0.74^**^	36	0.86^**^	36	0.72^**^	27	0.80^**^	36
Lexical decision	0.81^**^	38	0.67^**^	30	0.78^**^	38	0.85^**^	38	0.65^**^	30	0.79^**^	38
Semantic relationship judgment	0.84^**^	31	0.70^**^	31	0.78^**^	31	0.87^**^	31	0.68^**^	31	0.81^**^	31
Other	0.95^**^	21	0.71^**^	21	0.83^**^	21	0.90^**^	21	0.78^**^	21	0.84^**^	21
**Auditory comprehension total**			0.57^**^	83	0.80^**^	100			0.63^**^	83	0.80^**^	100
**ORAL PRODUCTION MODALITY**												
Confrontation picture naming	0.49^**^	79	0.89^**^	106	0.82^**^	106	0.59^**^	79	0.90^**^	106	0.84^**^	106
Repetition	0.47^**^	63	0.65^**^	64	0.61^**^	64	0.59^**^	63	0.72^**^	64	0.69^**^	64
Responsive speech and sentence completion	0.30	9	0.45	10	0.48	10	0.46	9	0.46	10	0.39	10
Sentence construction	0.79^**^	23	0.85^**^	24	0.90^**^	24	0.72^**^	23	0.86^**^	24	0.91^**^	24
Semantic opposites	0.82^**^	23	0.88^**^	25	0.89^**^	25	0.81^**^	23	0.85^**^	25	0.90^**^	25
Morphological derivates	0.87^**^	15	0.77^**^	15	0.80^**^	15	0.88^**^	15	0.85^**^	15	0.88^**^	15
Spontaneous and semi-spontaneous production	0.55^*^	17	0.73^**^	22	0.70^**^	22	0.48^*^	17	0.74^**^	22	0.69^**^	22
**Oral production total**					0.93^**^	113					0.94^**^	113
**OTHER MODALITIES**												
Reading aloud	0.40^**^	41	0.52^**^	33	0.65^**^	41	0.35^*^	41	0.30	33	0.55^**^	41
Written comprehension	0.83^**^	28	0.35	20	0.78^**^	28	0.61^**^	28	0.32	20	0.71^**^	28
Written production	0.38	23	0.53^**^	24	0.73^**^	24	0.50^*^	23	0.56^**^	24	0.69^**^	24
**UNCATEGORIZED MEASURES**												
	0.43^*^	27	0.75^**^	19	0.88^**^	27	0.30	27	0.52^*^	19	0.72^**^	27

* *p < 0.05*,

** *p < 0.01*.

### Interrater Reliability

In the beginning of the coding stage, the authors coded three studies together and agreed on the coding criteria. Disagreements were resolved via discussion. Then the first author coded 40 studies, 62%, and the second and the third authors coded the remaining studies. Later we randomly selected 16 studies, 25%, which were coded by two authors. For the language use and premorbid proficiency variables, which often required decision making, all studies were coded by two authors and any discrepancies were resolved by discussion including three authors. The Cohen's kappa values suggested strong interrater agreement for both language use, *k* = 0.807, *p* < 0.001, and proficiency, *k* = 0.818, *p* < 0.001 variables (Fleiss et al., 2003).

### Statistical Analysis

The *metafor* R package (Viechtbauer, 2010) was used for statistical analysis. To estimate effect sizes for the difference in performance between L1 and L2, we calculated risk ratios with the help of *escalc* function. According to the documentation of *escalc*, the argument RR provides logarithms of risk ratios, making them symmetric around zero as well as helping to decrease the positive skew in their distribution. The effect sizes in our sample are independent, because each effect size represents the difference in performance between L1 and L2 for a specific case.

First, we fitted random-effect models with the help of *rma* function to investigate whether there were differences in performance between L1 and L2 for the three types of scores: overall performance, auditory comprehension, and oral production. Then we performed the moderator analysis fitting mixed-effect models with the help of the same function to explore whether the possible difference in performance between L1 and L2 may be affected by the four variables of interest (i.e., early-late bilingual status, premorbid language proficiency, language use, and linguistic similarity). In addition to the moderator analysis on the early-late bilingual status variable, we analyzed whether AoA as a continuous variable moderates the outcomes in the early and late subgroups separately. The overall and moderator analyses were performed for the whole sample as well as for the early and late AoA subgroups, as well as separately for overall performance, auditory comprehension, and oral production scores.

Additionally, it was explored how participants' age at the time of assessment, years of education, and months post onset moderated the magnitude of the difference in performance between L1 and L2. The R scripts used for the analysis as well as the detailed report of the analysis are provided in Supplementary Material.

## Results

### Data Screening

**Figure 2:**
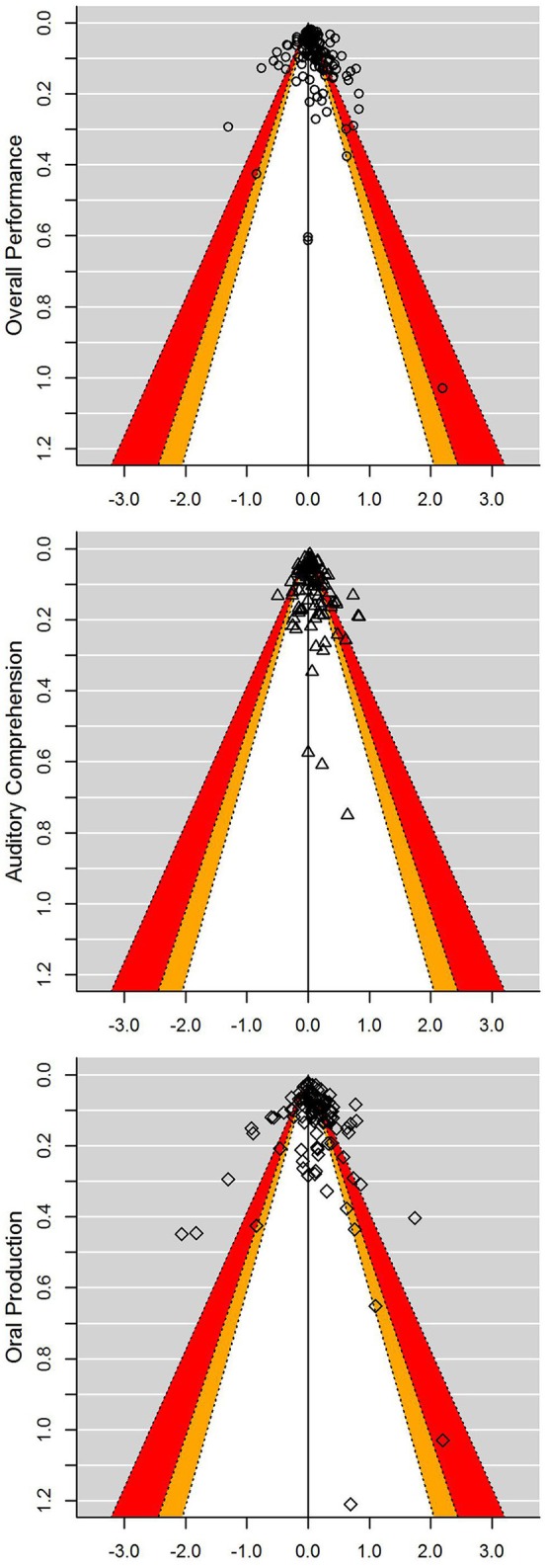
Contour enhanced funnel plots for each of the three types of scores analyzed. Contours change shades at *p*-levels 0.1 (white), 0.05 (orange), and 0.01 (red). Logarithms of risk ratios are plotted against the *SEs*, and the reference line indicating the random-effects model estimates for each the three types of scores analyzed. Positive and negative abscissas represent better performance in L1 and L2, respectively.

Three funnel plots, each showing distribution of effect sizes for overall performance, auditory comprehension, and oral production, were created to detect cases with immensely high standard errors (*SEs*) (see Figure 2). The standard error in the present analysis depended on the number of items used to assess a certain language modality: as the number of the tested items increases, SE gets smaller, and the precision gets higher. Based on visual examination of the funnel plots for overall performance, auditory comprehension, and oral production, the cut-off point was set at *SE* = 0.3. Thus, five, five, and 16 cases were removed for overall performance, auditory comprehension, and oral production scores, respectively. Given that large differences in performance between languages in the clinical population of persons with aphasia are meaningful and highly probable, we did not remove the data points with relatively large effect sizes. After deleting cases based on *SEs, log (RR)* = −1.30 had the largest absolute value among the datapoints from all three funnel plots. This value meant that in this case performance in L1 was 73% worse than in L2.

### Descriptive Characteristics

A total of 65 peer-reviewed published studies, from which 130 cases were extracted, were included in the review. Given that the analysis we performed required having information on which language was acquired first, six cases representing simultaneous bilinguals who acquired both languages from the age of zero, were excluded from the sample.

Twenty seven (22%), 65 (52%), and 32 (26%) cases were taken from group (*n* = 4), multi-case (*n* = 19), and single-case studies (*n* = 32), respectively. Sixty two (50%) cases were extracted from studies with research questions unrelated to testing differences between the languages of multilingual people with aphasia (*n* = 31); the remaining 62 (50%) cases were extracted from studies with research questions related to testing differences between one's languages (*n* = 24). Detailed information about the cases is summarized in Data Sheet 1 in Supplementary Material. Further analysis performed on the trimmed data showed that the study type (i.e., research question related vs. unrelated to testing L1/L2 differences) did not significantly moderate the outcomes for overall performance, *Q*_*M*_[1] = 2.89, *p* = 0.24, auditory comprehension, *Q*_*M*_[1] = 0.21, *p* = 0.90,or oral production, *Q*_*M*_[1] = 0.76, *p* = 0.68.

Descriptive information on the demographic and clinical details of the sample used for the analysis as well as the early and late AoA subgroups is summarized in Table 3.

**Table 3:** Demographic and clinical details of the whole sample and AoA subgroups.

**Characteristics**	**Whole group**			**Early AoA subgroup (< 7 year)**			**Late AoA subgroup (≥7 year)**		
	***N* = 119**			***n* = 44**			***n* = 75**		
	***Mean***	***SD***	**Range**	***Mean***	***SD***	**Range**	***Mean***	***SD***	**Range**
Age, year	58.5	14	17 - 91	52.9	14.2	17 - 84	61.8	12.9	33 - 91
Education, year	12.2	5.1	1 - 22	13.2	3.9	8 - 22	11.7	5.7	1 - 22
Female, % (*n*)	48% (57)			48% (21)			48% (36)		
Months post onset	28.3	14.9	1 - 53	28.3	15	2 - 53	28.3	15.0	1 - 52
AoA of L2, year	12.2	8.6	2.5 - 40	4.1	1.3	2.5 - 6	17.1	7.3	7 - 40
Lesion side: *n*	Left: 100; Right: 5; Both: 1; NA: 13			Left: 39; Right: 3; Both: 1; NA: 1			Left: 61; Right: 2; Both: 0; NA: 12		
Proficiency: *n*	L1: 27; Equal: 63; L2: 4; NA: 25			L1: 3; Equal: 30; L2: 4; NA: 7			L1: 24; Equal: 33; L2: 0; NA: 18		
Language use: *n*	L1: 27; Equal: 52; L2: 24; NA: 16			L1: 5; Equal: 21; L2: 13; NA: 5			L1: 22; Equal: 31; L2: 11; NA: 11		
Linguistic similarity, 2 levels: *n*	Similar: 90; Different: 29			Similar: 28; Different: 16			Similar: 62; Different: 13		
Linguistic similarity, 3 levels: *n*	Very close: 21; Close: 69; Different: 29			Very close: 7; Close: 21; Different: 16			Very close: 14; Close: 48; Different: 13		

### Language Status

**Figure 3:**
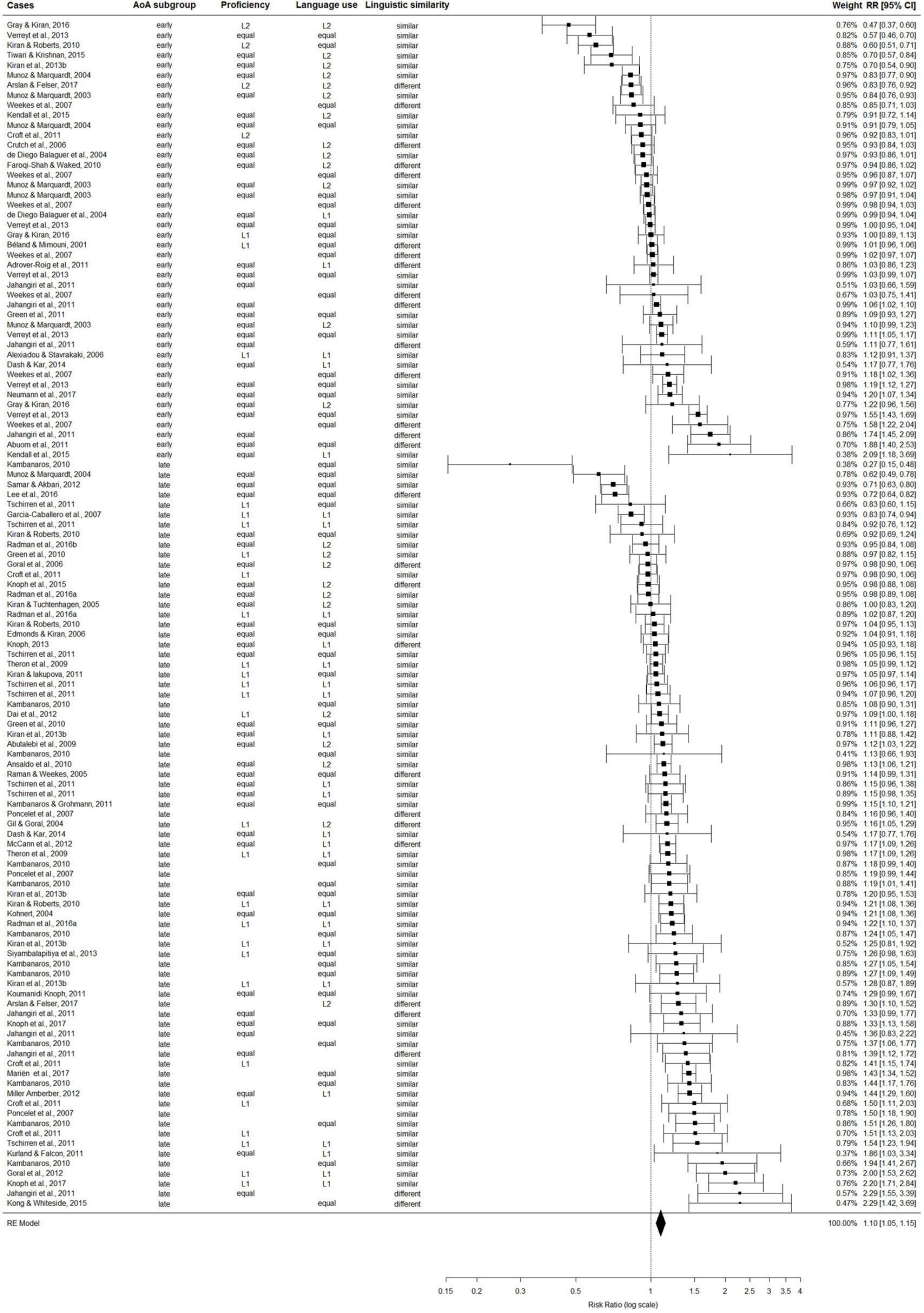
For the whole trimmed sample (*k* = 119), the figure displays effect sizes (Risk Ratios) and corresponding 95% confidence intervals (CI) for the comparison between overall language performance in the earlier-acquired (L1) and later-learned (L2) languages. Values larger than one indicate better performance in L1 compared to L2 and values smaller than one indicate worse performance in L1 compared to L2.

After data trimming, the difference in performance between L1 and L2 was investigated using overall performance scores. We found a statistically significant effect size, *RR* = 1.10 [1.05, 1.15], *p* < 0.0001, *Q*_*E*_ [118] = 1025.14, suggesting that overall performance in L1 was on average 10% better than in L2 (see Figure 3). For auditory comprehension scores, we also found a statistically significant effect size, *RR* = 1.06 [1.02, 1.10], *p* < 0.0001, *Q*_*E*_ [90] = 363.41, suggesting that auditory comprehension in L1 was on average 6% better than in L2. Similarly, a statistically significant effect size, *RR* = 1.10 [1.03, 1.17], *p* < 0.0001, *Q*_*E*_ [90] = 686.25, was found for oral production scores suggesting that performance in L1was on average 10% better than in L2.

### Age of Language Acquisition

Details of the moderator analysis (effects sizes, 95% CIs, and statistics of the moderator tests) are summarized in Table 4.

**Table 4:** Details of the moderator analysis.

**Moderators**	**Overall performance**						**Auditory comprehension**						**Oral production**					
	**Effect size (RR)**	**95% CI**	***k***	***Q_***M***_***	***df***	***p***	**Effect size (RR)**	**95% CI**	***k***	***Q_***M***_***	***df***	***p***	**Effect size (RR)**	**95% CI**	***k***	***Q_***M***_***	***df***	***p***
**WHOLE GROUP**																		
**AoA in years**	1.01^**^	[1.00, 1.01]	116	**8.84**	**1**	**<0.01**	1.00	[1.00, 1.01]	91	1.65	1	0.20	1.01^***^	[1.01, 1.02]	88	**13.61**	**1**	**<0.001**
**AoA status**			119	**11.37**	**1**	**<0.001**			91	2.59	1	0.11			91	**8.85**	**1**	**<0.01**
Early (AoA < 7 year)	1.00	[0.93, 1.07]	44				1.03	[0.97, 1.09]	38				0.97	[0.88, 1.07]	32			
Late (AoA ≥ 7 year)	1.16^***^	[1.10, 1.23]	75				1.09^***^	[1.04, 1.15]	53				1.17^***^	[1.09, 1.26]	59			
**Premorbid proficiency**			90	2.87	1	0.09			78	0.05	1	0.82			71	**6.13**	**1**	**<0.05**
Higher in L1	1.16^***^	[1.06, 1.26]	27				1.07	[0.99, 1.15]	25				1.23^**^	[1.08, 1.38]	19			
Equal	1.06^*^	[1.01, 1.12]	63				1.08^**^	[1.03, 1.13]	53				**1.03**	[0.95, 1.10]	52			
Higher in L2	0.70^**^	[0.57, 0.87]	4				0.88	[0.75, 1.03]	4				0.71^*^	[0.53, 0.97]	3			
**Language use**			103	**12.48**	**2**	**<0.01**			79	**6.49**	**2**	**<0.05**			79	**6.29**	**2**	**<0.05**
More in L1	1.19^***^	[1.09, 1.30]	27				1.11^**^	[1.03, 1.19]	22				1.26^**^	[1.09, 1.46]	17			
Equal	1.09^*^	[1.02, 1.16]	52				1.05	[0.99, 1.10]	34				1.07	[0.97, 1.17]	41			
More in L2	0.95	[0.87, 1.04]	24				0.98	[0.93, 1.04]	23				0.99	[0.87, 1.12]	21			
**Linguistic similarity**			119	0.35	1	0.55			91	0.45	1	0.50			91	0.06	1	0.81
Different	1.12^*^	[1.03, 1.23]	29				1.08^*^	[1.01, 1.16]	25				1.08	[0.94, 1.24]	18			
Similar	1.09^**^	[1.03, 1.15]	90				1.05^*^	[1.01, 1.10]	66				1.10^**^	[1.03, 1.18]	73			
**Age in years**	1.00^**^	[1.00, 1.01]	119	**8.71**	**1**	**<0.01**	1.00^*^	[1.00, 1.01]	91	**5.70**	**1**	**<0.05**	1.00	[1.00, 1.01]	91	3.72	1	0.05
**Years of education**	0.99^*^	[0.98, 1.00]	71	**3.90**	**1**	**<0.05**	1.00	[0.99, 1.01]	52	0.13	1	0.72	0.99	[0.97, 1.00]	63	3.04	1	0.08
**Months post onsent**	1.00	[1.00, 1.00]	106	0.97	1	0.32	1.00	[1.00, 1.00]	79	1.18	1	0.28	1.00	[1.00, 1.01]	85	2.04	1	0.15
**EARLY AoA SUBGROUP**																		
***AoA in years***	0.96	[0.91, 1.02]	44	1.72	1	0.19	0.97	[0.93, 1.01]	38	2.83	1	0.09	0.99	[0.91, 1.08]	32	0.06	1	0.80
**Premorbid proficiency**				-	-	-				-	-	-				-	-	-
Higher in L1	1.04	[0.80, 1.35]	3				1.04	[0.86, 1.26]	3				0.96	[0.64, 1.44]	2			
Equal	1.03	[0.95, 1.13]	30				1.06	[0.99, 1.15]	24				1.01	[0.90, 1.13]	27			
Higher in L2	0.70^**^	[0.55, 0.88]	4				0.88	[0.74, 1.04]	4				0.71^*^	[0.51, 0.99]	3			
**Language use**			34	**5.14**	**1**	**<0.05**			31	**6.57**	**1**	**<0.05**			24	0.50	1	0.48
More in L1	1.13	[0.90, 1.42]	5				0.97	[0.83, 1.13]	3				1.21	[0.86, 1.69]	4			
Equal	1.04	[0.94, 1.15]	21				1.06	[1.00, 1.12]	19				0.97	[0.82, 1.15]	14			
More in L2	0.86^*^	[0.76, 0.98]	13				0.94	[0.88, 1.00]	12				0.88	[0.73, 1.07]	10			
**Linguistic similarity**			44	2.64	1	0.10			38	0.04	1	0.83			32	1.73	1	0.19
Different	1.08	[0.96, 1.22]	16				1.04	[0.95, 1.13]	15				1.10	[0.89, 1.34]	8			
Similar	0.96	[0.87, 1.05]	28				1.02	[0.95, 1.10]	23				0.94	[0.83, 1.06]	24			
**Age in years**	1.00	[1.00, 1.01]	44	1.20	1	0.27	1.00	[1.00, 1.01]	38	0.98	1	0.32	1.00	[0.99, 1.01]	32	0.09	1	0.76
**Years of education**	0.99	[0.97, 1.01]	26	0.74	1	0.39	1.00	[0.98, 1.01]	22	0.02	1	0.89	0.98	[0.95, 1.02]	19	0.60	1	0.44
**Months post onsent**	1.00	[1.00, 1.01]	32	1.31	1	0.25	1.00	[0.99, 1.00]	27	0.71	1	0.40	1.01	[1.00, 1.01]	27	3.15	1	0.08
**LATE AoA SUBGROUP**																		
***AoA in years***	1.00	[1.00, 1.01]	72	1.41	1	0.24	1	[1.00, 1.01]	53	0.23	1	0.63	1.01^*^	[1.00, 1.02]	56	**6.15**	**1**	**<0.05**
**Premorbid proficiency**			57	1.60	1	0.21			51	0.11	1	0.74			42	**5.49**	**1**	**<0.05**
Higher in L1	1.17^***^	[1.08, 1.28]	24				1.07	[0.99, 1.16]	22				1.26^***^	[1.12, 1.42]	17			
Equal	1.09^*^	[1.02, 1.17]	33				1.09^*^	[1.02, 1.16]	29				1.05	[0.95, 1.16]	25			
Higher in L2	-	-	0				-	-	0				-	-	0			
**Language use**			64	2.77	2	0.25			45	3.46	2	0.18			51	2.20	2	0.33
More in L1	1.20^***^	[1.09, 1.32]	22				1.13^**^	[1.05, 1.23]	19				1.28^**^	[1.09, 1.49]	13			
Equal	1.12^**^	[1.04, 1.21]	31				1.03	[0.94, 1.12]	15				1.13^*^	[1.01, 1.26]	27			
More in L2	1.06	[0.93, 1.19]	11				1.03	[0.95, 1.13]	11				1.10	[0.93, 1.30]	11			
**Linguistic similarity**			75	0.02	1	0.88			53	1.49	1	0.22			59	1.60	1	0.21
Different	1.17^*^	[1.04, 1.32]	13				1.16^**^	[1.04, 1.29]	10				1.07	[0.90, 1.26]	10			
Similar	1.16^***^	[1.09, 1.23]	62				1.07^*^	[1.02, 1.14]	43				1.20^***^	[1.11, 1.29]	49			
**Age in years**	1.00	[1.00, 1.01]	75	3.36	1	0.07	1.00	[1.00, 1.01]	53	3.38	1	0.07	1.00	[1.00, 1.01]	59	2.40	1	0.12
**Years of education**	0.99	[0.98, 1.00]	45	2.36	1	0.12	0.99	[0.98, 1.01]	30	0.45	1	0.50	0.99	[0.98, 1.01]	44	1.39	1	0.24
**Months post onsent**	1.00	[1.00, 1.00]	74	0.16	1	0.69	1.00	[1.00, 1.00]	52	0.79	1	0.38	1.00	[1.00, 1.00]	58	0.01	1	0.92

* *p < 0.05*,

** *p < 0.01*,

*** *p < 0.001*.

*Bold values represent statistically significant results*.

In the whole sample, AoA as a continuous variable moderated overall performance, *Q*_*M*_[1] = 8.84, *p* < 0.01, and oral production, *Q*_*M*_[1] = 13.61, *p* < 0.001, in the direction that as AoA increased, the magnitude of the L1 advantage (better performance in L1 compared to L2) increased. AoA as a continuous variable did not moderate auditory comprehension in the whole sample, *Q*_*M*_[1] = 1.65, *p* = 0.20.

To decide on the cut-off point for making the early and late AoA subgroups, we visually explored the distribution of overall performance outcomes plotted against AoA as a continuous variable (see the plot in Data Sheet 3, p.53 in Supplementary Material). Based on this visual examination, 7 year appeared to be a reasonable cut-off point.

AoA status as a binary variable (early/late) significantly moderated overall performance, *Q*_*M*_[1] = 11.37, *p* < 0.001, and oral production, *Q*_*M*_[1] = 8.85, *p* < 0.01. Individuals who acquired L2 before 7 year showed a significantly smaller difference between L1 and L2 in overall performance, *RR* = 1.00 [0.93, 1.07], *p* = 0.99, compared to those who acquired L2 after 7 year, where performance in L1 was significantly better than in L2, *RR* = 1.16 [1.10, 1.23], *p* < 0.0001. Similarly for oral production, the early AoA subgroup showed a significantly smaller difference between L1 and L2, *RR* = 0.97 [0.88, 1.07], *p* = 0.60, compared to the late AoA subgroup, where performance in L1 was significantly better than in L2, *RR* = 1.17 [1.09, 1.26], *p* < 0.0001. The early-late bilingual status did not moderate auditory comprehension, *Q*_*M*_[1] = 2.59, *p* = 0.11.

Additionally, we explored how AoA as a continuous variable moderated the outcomes in the early and late AoA subgroups separately. In the early AoA subgroup, AoA did not moderate overall performance, *Q*_*M*_[1] = 1.72, *p* = 0.19, auditory comprehension, *Q*_*M*_[1] = 2.83, *p* = 0.09, or oral production, *Q*_*M*_[1] = 0.06, *p* = 0.80. In the late AoA subgroup, AoA moderated oral production, *Q*_*M*_[1] = 6.15, *p* < 0.05, but not overall performance, *Q*_*M*_[1] = 1.41, *p* = 0.24, or auditory comprehension, *Q*_*M*_[1] = 0.23, *p* = 0.63.

### Premorbid Language Proficiency

Given that there were only four effect sizes in the higher L2 proficiency group, they were excluded from the analysis and described separately. In the whole group, premorbid language proficiency did not moderate either overall performance *Q*_*M*_[1] = 2.87, *p* = 0.09, or auditory comprehension, *Q*_*M*_[1] = 0.05, *p* = 0.82. Proficiency significantly moderated oral production, *Q*_*M*_[1] = 6.13, *p* < 0.05. Individuals with equal proficiency in both languages had a significantly smaller difference between L1 and L2 in oral production, *RR* = 1.03 [0.95, 1.10], *p* = 0.49, compared to those who were more proficient in L1, *RR* = 1.23 [1.08, 1.38], *p* < 0.01. Individuals with higher L2 proficiency performed significantly better in L2 overall, *RR* = 0.70 [0.57, 0.87], *p* < 0.01, and in oral production, *RR* = 0.71 [0.53, 0.97], *p* < 0.05, but not in auditory comprehension, *RR* = 0.88 [0.75, 1.03], *p* = 0.12.

Given that 81% (*n* = 30) of the cases in the early AoA subgroup had equal proficiency and other two proficiency groups included three (L1) and four (L2) cases each, the moderator analysis was not performed and only descriptive statistics are reported here. Individuals with higher L1 proficiency performed comparably in the two languages overall, *RR* = 1.04 [0.80, 1.35], *p* = 0.77, as well as in auditory comprehension, *RR* = 1.04 [0.86, 1.26], *p* = 0.67, and oral production, *RR* = 0.96 [0.64, 1.44], *p* = 0.84. Similarly, participants with equal proficiency performed comparably overall, *RR* = 1.03 [0.95, 1.13], *p* = 0.47, as well as in auditory comprehension, *RR* = 1.06 [0.99, 1.15], *p* = 0.10, and oral production, *RR* = 1.01 [0.90, 1.13], *p* = 0.84. In contrast, the four participants with higher L2 proficiency described above were all early bilinguals; again, they showed significantly better performance in L2 overall, *RR* = 0.70 [0.55, 0.88], *p* < 0.01, as well as in oral production, *RR* = 0.71 [0.51, 0.99], *p* < 0.05, but not in auditory comprehension, *RR* = 0.88 [0.74, 1.04], *p* = 0.13.

In the late AoA subgroup, where 58% (*n* = 33) had equal proficiency and the rest (*n* = 24) had higher L1 proficiency, language proficiency did not moderate overall performance, *Q*_*M*_[1] = 1.60, *p* = 0.21, and auditory comprehension, *Q*_*M*_[1] = 0.11, *p* = 0.74, but was a significant moderator for oral production, *Q*_*M*_[1] = 5.49, *p* < 0.05. Individuals with higher L1 proficiency performed significantly better in L1, *RR* = 1.26 [1.12, 1.42], *p* < 0.001, whereas the equal proficiency group showed comparable performance in oral production, *RR* = 1.05 [0.95, 1.16], *p* = 0.36.

### Language Use

For the whole group, language use moderated overall performance, *Q*_*M*_[2] = 12.48, *p* < 0.01, auditory comprehension, *Q*_*M*_[2] = 6.49, *p* < 0.05, and oral production, *Q*_*M*_[2] = 6.29, *p* < 0.05. Individuals who premorbidly used L1 more frequently had significantly greater magnitude of L1 advantage in overall performance, *RR* = 1.19 [1.09, 1.30], *p* < 0.001, as well as individuals who equally used both languages, *RR* = 1.09 [1.02, 1.16], *p* < 0.05, compared to the group with more L2 use who showed comparable performance in both languages, *RR* = 0.95 [0.87, 1.04], *p* = 0.25. Individuals who premorbidly used L1 more frequently performed significantly better in L1 in both auditory comprehension, *RR* = 1.11 [1.03, 1.19], *p* < 0.01, and oral production, *RR* = 1.26 [1.09, 1.46], *p* < 0.01, compared to the L2 more frequent usage group who again showed comparable performance in both languages, (auditory comprehension: *RR* = 0.98 [0.93, 1.04], *p* = 0.60; oral production: *RR* = 0.99 [0.87, 1.12], *p* = 0.84). There were no significant differences between the more L1 and equal use groups for overall performance, auditory comprehension, and oral production.

Given that there were only five individuals in the early AoA group with greater L1 use, they were excluded from the moderator analysis and described separately. In the early AoA subgroup, language use moderated the outcomes for overall performance, *Q*_*M*_[1] = 5.14, *p* < 0.05, and auditory comprehension, *Q*_*M*_[1] = 6.57, *p* < 0.05, but not for oral production, *Q*_*M*_[1] = 0.50, *p* = 0.48. In overall performance, individuals with more frequent L2 use showed significantly better performance in L2, *RR* = 0.86 [0.76, 0.98], *p* < 0.05, compared to those with equal use, who showed comparable overall performance, *RR* = 1.04 [0.94, 1.15], *p* = 0.43. Similarly for auditory comprehension, individuals with more L2 use showed comparable performance with a trend toward better performance in L2, *RR* = 0.94 [0.88, 1.00], *p* = 0.07, whereas the equal use group showed comparable performance with a trend toward better performance in L1, *RR* = 1.06 [1.00, 1.12], *p* = 0.07. The more L1 use group showed comparable performance overall, *RR* = 1.13 [0.90, 1.42], *p* = 0.27, in auditory comprehension, *RR* = 0.97 [0.83, 1.13], *p* = 0.70, and oral production, *RR* = 1.21 [0.86, 1.69], *p* = 0.27.

For the late AoA subgroup, language use did not moderate overall performance, *Q*_*M*_[2] = 2.77, *p* = 0.25, auditory comprehension, *Q*_*M*_[2] = 3.46, *p* = 0.18, or oral production, *Q*_*M*_[2] = 2.20, *p* = 0.33.

### Linguistic Similarity

For the whole group, binary linguistic similarity (different/similar languages) did not moderate overall performance, *Q*_*M*_[1] = 0.35, *p* = 0.55, auditory comprehension, *Q*_*M*_[1] = 0.45, *p* = 0.50, or oral production, *Q*_*M*_[1] = 0.06, *p* = 0.81.

For the early AoA subgroup, binary linguistic similarity did not moderate overall performance, *Q*_*M*_[1] = 2.64, *p* = 0.10, auditory comprehension, *Q*_*M*_[1] = 0.04, *p* = 0.83, or oral production, *Q*_*M*_[1] = 1.73, *p* = 0.19.

For the late AoA subgroup, binary linguistic similarity did not moderate overall performance, *Q*_*M*_[1] = 0.02, *p* = 0.88, auditory comprehension, *Q*_*M*_[1] = 1.49, *p* = 0.22, or oral production, *Q*_*M*_[1] = 1.60, *p* = 0.21.

Similarly, linguistic similarity coded with three levels (very close/close/different languages) did not appear to be a significant moderator for overall performance (whole group: *k* = 119, *Q*_*M*_[2] = 0.78, *p* = 0.68; early: *k* = 44, *Q*_*M*_[2] = 2.87, *p* = 0.24; late: *k* = 75, *Q*_*M*_[2] = 1.96, *p* = 0.38), auditory comprehension (whole group: *k* = 91, *Q*_*M*_[2] = 0.52, *p* = 0.77; early: *k* = 38, *Q*_*M*_[2] = 0.05, *p* = 0.98; late: *k* = 53, *Q*_*M*_[2] = 1.49, *p* = 0.48), or oral production (whole group: *k* = 91, *Q*_*M*_[2] = 0.17, *p* = 0.92; early: *k* = 32, *Q*_*M*_[2] = 1.99, *p* = 0.37; late: *k* = 59, *Q*_*M*_[2] = 2.08, *p* = 0.35).

### Additional Variables

In the whole sample, age moderated the outcomes for overall performance, *Q*_*M*_[1] = 8.71, *p* < 0.01, and auditory comprehension, *Q*_*M*_[1] = 5.70, *p* < 0.05: as age increased, the magnitude of L1 advantage increased. There was no significant moderation for oral production, *Q*_*M*_[1] = 3.72, *p* = 0.054. Years of education moderated overall performance, *Q*_*M*_[1] = 3.90, *p* < 0.05: as years of education increased, the magnitude of L1 advantage decreased. There were no significant effects of education either for auditory comprehension, *Q*_*M*_[1] = 0.13, *p* = 0.72, or for oral production, *Q*_*M*_[1] = 3.04, *p* = 0.08. Months post onset did not moderate overall performance, *Q*_*M*_[1] = 0.97, *p* = 0.32, auditory comprehension, *Q*_*M*_[1] = 1.18, *p* = 0.28, or oral production, *Q*_*M*_[1] = 2.04, *p* = 0.15.

When the AoA subgroups were analyzed separately, age did not moderate outcomes for overall performance (early: *Q*_*M*_[1] = 1.20, *p* = 0.27; late: *Q*_*M*_[1] = 3.36, *p* = 0.07), auditory comprehension (early: *Q*_*M*_[1] = 0.98, *p* = 0.32; late: *Q*_*M*_[1] = 3.38, *p* = 0.07), or oral production (early: *Q*_*M*_[1] = 0.09, *p* = 0.76; late: *Q*_*M*_[1] = 2.40, *p* = 0.12). Similarly, years of education did not moderate overall performance (early: *Q*_*M*_[1] = 0.74, *p* = 0.39; late: *Q*_*M*_[1] = 2.36, *p* = 0.12), auditory comprehension (early: *Q*_*M*_[1] = 0.02, *p* = 0.89; late: *Q*_*M*_[1] = 0.45, *p* = 0.50), or oral production (early: *Q*_*M*_[1] = 0.60, *p* = 0.44; late: *Q*_*M*_[1] = 1.39, *p* = 0.24). Finally, months post onset did not moderate overall performance (early: *Q*_*M*_[1] = 1.31, *p* = 0.25; late: *Q*_*M*_[1] = 0.16, *p* = 0.69), auditory comprehension (early: *Q*_*M*_[1] = 0.71, *p* = 0.40; late: *Q*_*M*_[1] = 0.79, *p* = 0.38),or oral production (early: *Q*_*M*_[1] = 3.15, *p* = 0.08; late: *Q*_*M*_[1] = 0.01, *p* = 0.92).

## Discussion

The questions motivated this systematic review were whether people with aphasia are likely to exhibit better performance in their first-acquired (L1) than in a later-learned (L2) language, and whether age of acquisition (AoA), premorbid language proficiency, use and exposure, and linguistic similarity between the person's languages affect the consequences of aphasia in L1 and L2. We followed the PRISMA guidelines for a systematic review (Gates and March, 2016) and included 65 studies and 130 bilingual individuals with aphasia. Meta-analyses of effects sizes revealed the following answers to our questions.

### L1 Primacy

We found that in the 119 bilingual speakers included in the analysis as a group, L1 was significantly better preserved than L2. This finding could be considered at odds with the view that different languages are processed in shared neural substrata for bilingual speakers (e.g., Abutalebi, 2008) and with the view held by many researchers and clinicians that bilingual people with aphasia tend to show equivalent language impairments after a stroke. The comparable impairment view has been supported by several reports in the literature. For example, Fabbro (2001) identified equivalent impairments in L1 and L2 in ≈60% of the cases he reviewed, who were early bilinguals with high proficiency in both languages. Unlike the findings reported by Fabbro (2001) and those reported in Albert and Obler (1978), our results appear to support Ribot (1882), which predicts that the earlier acquired language is more resistant to brain damage. This is also consistent with findings of better preservation in aphasia of words that are learned early in life compared to those learned later in life (for review see Brysbaert and Ellis, 2016).

We contend that our more rigorous analysis, which included a larger number of participants from a diversity of multilingual speakers of typologically different languages, is more reliable than the conclusions drawn from prior reviews. We note that the effect of language status (L1 vs. L2) confirmed here is often seen in case reports of bilingual speakers with aphasia but has rarely been analyzed according to the criteria developed in the present review.

Moreover, there has been a tendency in the literature on bilingual aphasia toward reporting performance of single cases according to the question of whether language impairments are parallel or differential (Paradis, 1983; Fabbro, 2001). We believe that posing this question can be misleading. It is critical to first determine whether parallel impairments should be expected, depending on the characteristics of the bilingual individuals. Indeed, it is possible that the reports of ≈60% of bilingual participants with aphasia demonstrating comparable impairments in both their L1 and L2 found in previous reviews are driven by early bilinguals and misrepresent the state of affairs for late bilinguals. We therefore divided our sample into early and late bilinguals to examine the observed difference between L1 and L2 separately for the two types of bilinguals. Furthermore, we examined whether additional bilingual characteristics, namely, specific AoA, frequency of language use, premorbid language proficiency, and linguistic similarity moderate the difference between L1 and L2.

### Age of Language Acquisition

When we examined AoA as a binary categorical variable, our results demonstrated significant differences between early and late bilinguals. Specifically, late bilinguals, who acquired their other language after the age of seven, showed significantly better overall performance in L1 than in the later-learned language. In contrast, the early bilinguals who acquired their languages before the age of seven showed comparable performance in both languages. This result is consistent with previous findings from reports of balanced bilingual speakers who showed comparable levels of impairment (e.g., Fabbro, 2001; Kiran and Roberts, 2010). This difference between the two subgroups was significant despite the fact that the majority of individuals in both subgroups had equal pre-stroke proficiency in both their languages (81% and 58% in the early and late bilingual subgroups, respectively). Our finding of an effect of language status (i.e., significant difference between L1 and L2 performance) post stroke for late bilinguals challenges the assumptions of the shared bilingual neural substrate (SBNS) and the convergence hypothesis (Green, 2003). It is also at odds with the conclusions of Tschirren et al. (2011). It is possible that the differences in syntactic processing reported by Tschirren et al. (2011), together with generally comparable impairment, are the sort of outcomes that have contributed to the differential findings our meta-analysis revealed.

We found that, in the whole sample, AoA as a continuous variable moderated overall performance, oral production, but not auditory comprehension. This is consistent with findings that in bilinguals who are not highly proficient, language production is typically more difficult than language comprehension (e.g., Swain, 1985). It is possible that the substantial variance of performance among the late bilinguals (but not in the early bilinguals) included here allowed for the effect of AoA to emerge. Future studies could further examine the AoA at which the patterns of results change. Of interest, we found an effect of age, with older individuals showing the greater magnitude of L1 advantage compared to younger ones; the interaction of age and AoA could be further examined in future studies.

Thus, AoA moderated performance differences between L1 and L2 when early and late bilinguals were compared, which may suggest that a language that is acquired early enjoys a unique status and could potentially be differentially processed in the brain (e.g., Giussani et al., 2007). In contrast, the finding that AoA as a continuous variable significantly moderated only oral production in the late AoA subgroup only suggests that the exact AoA matters less. This is consistent with some views of the role of AoA in bilingualism (Birdsong and Molis, 2001). We note that we divided the participants into the early and late subgroups based on a theoretically motivated rationale. We found that in our sample, age 7 year was a natural breakpoint, considering that individuals started schooling in L2 at this age. A similar cut-off point (6 year) for early and later AoA was also used in the meta-analytic review on the bilingual advantage by Lehtonen et al. (2018).

Our findings of better overall performance in L1 than in L2 have implications for the cognitive neuropsychology of bilingual aphasia as well as for clinical aphasiology. Nevertheless, as expected, this finding was qualified by several variables identified in the literature as potential moderators: premorbid language proficiency, language use, and linguistic similarity (e.g., Goral et al., 2006; Ansaldo et al., 2008; Lorenzen and Murray, 2008). It can be argued that language proficiency and language use are typically correlated. As a rule, speakers who use a language with frequency and regularity are more likely to be highly proficient in that language (e.g., Gollan et al., 2015; Peñaloza et al., 2017). However, there are also instances in which people report greater use than proficiency, especially in L2. For the individuals included in the analysis in the current review, there was a significant association between these two variables (*n* = 85 *p* < 0.01, Cramer's V = 0.34); in our analyses, we examined the effects of language proficiency and language use separately.

### Premorbid Language Proficiency

We tested whether premorbid language proficiency moderated the magnitude of the difference in performance between L1 and L2. One could assume that a premorbidly more proficient language would be better preserved after a stroke. Our results partially supported this hypothesis. Individuals with higher L1 proficiency and those with equal proficiency in their two languages showed the pattern observed for the sample as a whole, namely, better overall performance in L1 when compared to L2. There were only four individuals in the sample who reported higher premorbid L2 proficiency than L1 proficiency and they appeared to perform better in L2 compared to L1. No statistically significant differences were found between the higher premorbid L1 proficiency group compared with the equal proficiency group in overall performance and auditory comprehension scores, however the magnitude of L1 advantage in oral production scores was significantly greater for the group with higher L1 proficiency. These results overall suggest that L2 proficiency plays a role in the degree of impairment only when it surpasses the proficiency in L1. Given that the higher L2 proficiency group was very small in the present review, this assumption requires further investigation.

We also examined how proficiency moderated the effect of language status in the early bilingual and late bilingual subgroups separately. We observed that in the early bilingual group, individuals with higher L1 and with equal proficiency showed the pattern observed for the subgroup as a whole, namely, comparable performance in both languages. The four individuals who reported higher premorbid L2 proficiency were all early bilinguals and, as mentioned above, performed better in L2. There were no effects of proficiency in the late bilingual subgroup except for oral production, for which the magnitude of L1 advantage was significantly bigger for those individuals who reported higher L1 proficiency than those who reported to be equally proficient in both languages.

Thus, language proficiency appears to have a relatively small role in the results of overall differences between L1 and L2, except for those cases where L2 achieved higher proficiency than L1. This finding does not support the view that language proficiency has a greater role in determining language representation and processing in bilinguals than AoA (e.g., Perani et al., 1998; Abutalebi et al., 2001). We also found that the more years of education individuals had, the smaller was the magnitude of L1 advantage. This suggests that education in L2 could be used as an additional source of information for determining premorbid L2 proficiency.

It is of interest to note how language proficiency was measured in the reviewed studies. There was great variability in the measures and tools used (e.g., section A of the BAT; the Language Use Questionnaire, Muñoz et al., 1999), but generally, most studies included subjective self-ratings of the participants of their language abilities prior to the stroke. These self-ratings ranged in terms of the size of the scale and whether each ability was rated separately. In a few cases, family members' ratings were included as well. In none of the studies, formal measures of premorbid language abilities (e.g., language proficiency test, language placement test) were available.

### Language Use

Language use has been discussed in recent publications on bilingual language performance (Linck et al., 2009), as a determining variable in degree of impairment as well as degree of recovery from aphasia (Goral et al., 2012, 2013; Knoph et al., 2017). This is particularly true for individuals who live in a monolingual L2 environment following immigration for example.

We examined whether the magnitude of the difference between L1 and L2 was influenced by language use. One could hypothesize that the more used language would be better preserved (Pitres, 1895/1983). Our results partially supported this hypothesis. In the whole group, those with more frequent use of L1 showed significantly better performance in L1 compared to L2 in all of the three outcomes, whereas those who rated their L2 use as more frequent than their L1 performed comparably in both languages in all three outcomes. For the early AoA subgroup, those who used L2 more often showed better performance in L2 based on overall performance scores, whereas those who used L1 more frequently and those who used both languages equally showed comparable performance in both languages in all three outcomes. Better performance in L2 compared to L1 was not found in the late bilinguals, whereas better performance in L1 and comparable performance were the typical patterns.

Similar to the findings for language proficiency in oral production performance, we found evidence of significantly greater magnitude of L1 advantage in the group with more frequent L1 use compared with the group where L2 was more frequently used, but not with the group where both languages were equally used. These findings suggest that language use affected the magnitude of L1 advantage when L2 became the most frequently used language. Thus, like premorbid language proficiency, language use has a moderating role on the findings, which does not seem to be independent of AoA.

### Linguistic Similarity

There has been discussion in the literature regarding the degree to which language similarity influences the comparability of impairment in bilingual aphasia (Lorenzen and Murray, 2008). Whereas, on the one hand, one might predict that more similar languages would look similarly impaired following a stroke, there is little evidence to support this prediction and there is controversy in the literature regarding the role of language similarity on the neuronal organization of the languages of a bilingual (Kumar, 2014; Wong et al., 2016). On the other hand, one could assume that because linguistically similar languages share a significant portion of lexico-semantic representation (e.g., cognates), more cognitive control may be required to overcome cross-language interference. Our analyses revealed no effect of linguistic similarity. This finding is consistent with recent studies that attributed greater importance of language proficiency and use than of linguistic similarity (e.g., Muñoz and Marquardt, 2003; Ansaldo and Saidi, 2014; Kastenbaum et al., 2019). The finding is also consistent with neuroimaging studies that have demonstrated overlap in processing and representation among languages of bilinguals even for those who use languages that are very different from each other (e.g., Abutalebi et al., 2001; Wong et al., 2016).

We note, however, that quite a few studies have reported an effect of cognates, which is one aspect of language similarity that has been studied in aphasia (Kohnert, 2004; Kurland and Falcon, 2011; Kendall et al., 2015). Our finding of no role of linguistic similarity could be considered in opposition to such studies. It is possible that linguistic similarity affects the manifestation of specific linguistic aspects, consistent with findings that reported interference between languages that are similar (Fabbro, 2001; Goral et al., 2006), but that the degree of language similarity does not affect overall relative levels of impairment. Thus, it may be that effects of linguistic similarity on performance will be evident in tasks that require syntactic processing for languages that share or differ in specific morpho-syntactic aspects (e.g., Nilipour and Paradis, 1995; Yiu and Worrall, 1996; Goral et al., 2010) and in those that demand lexical-semantic processing for languages that share more or fewer cognates (Kohnert, 2004; Kurland and Falcon, 2011). We also note that dissociations in performance for bilingual patients with reading and writing disorders suggest that language type can constrain patterns of bilingual aphasia (see Weekes, 2012; Goral, 2019).

## Limitations

The number of significant effect sizes we found points to the robustness of our findings, although the greater L2 proficiency results were based on a small number of cases and should be interpreted with caution. Furthermore, there was great variability among the studies included in the review, both in terms of the participants' characteristics and the language performance measures (see Table 1). Indeed, the variability of measures used is a limitation of the present data as well as of the field in general. Our data highlight the importance of greater uniformity of assessment in bilingual aphasia, which was one rationale for the development of the BAT (Paradis and Libben, 1987), although, other tools are clearly needed to assess specific languages and linguistic aspects.

We are mindful of drawing conclusions from the data beyond the domain of bilingual aphasia. Our results confirm the view that individual differences in the unique language background characteristics of a bilingual speaker are very likely to impact on the presentation of aphasia in more than one language. Additionally, an open question to date is to what degree differences in performance between L1 and L2 in late bilinguals are due to differential impairment levels or to differential pre-stroke mastery levels. Another conclusion which should be viewed with caution is the one regarding AoA. Although the transformation of the AoA into the binary classification (early/late) based on 7 year of age was motivated theoretically, other cut-off points can be considered in the future research. Furthermore, a lack of effects of specific AoA within the AoA subgroups could partially be a result of relatively low inter-individual variability in this variable.

Finally, the analyses we conducted did not allow us to consider in-depth language impairment patterns of multilingual individuals with aphasia, such as for instance, uncontrollable language blending and antagonistic recovery (Paradis, 1977, 2001), which are of great importance for understanding cognitive mechanisms of language. Moreover, given the cross-sectional nature of the present study, it does not inform us about the dynamics of language performance, which was an important aspect in the classification of recovery patterns in multilingual aphasia (Paradis, 1977, 2001).

## Conclusion

To conclude, the current systematic review and meta-analysis revealed a better performance in L1 compared to L2 in bilingual speakers with aphasia. It also demonstrated that the magnitude of this difference was moderated by whether the bilinguals learned their two languages early in childhood or later. The better performance in L1 was a robust finding, which was moderated by premorbid language proficiency and frequency of use. Finally, linguistic similarity did not appear to interact with the magnitude of the difference in performance between L1 and L2.

The results we report here from a meta-analysis reflect the patterns observed in case studies, case-series, and group studies of multilingual individuals with aphasia. Cognitive neuropsychology has been a dominant theoretical movement in the study of aphasia for nearly 50 years. One defining feature of cognitive neuropsychology is the study of the single case and its bedrock assumption is that group studies are not meaningful because they average data across participants and consequently mask individual differences (Caramazza, 1986; though see Caplan, 1988; Grodzinsky et al., 1999). In the past decade, cognitive neuropsychologists have evolved toward advocating a case-series approach which retains the individual differences in single cases while accommodating the general patterns of performance in clinical groups (Schwartz and Dell, 2010; Rapp, 2011). For this reason, the problems of averaging that are debated extensively in the cognitive neuropsychological literature (e.g., McCloskey and Caramazza, 1988) do not apply to case-series designs. We contend that the type of meta-analysis conducted here also retains the individual patterns of performance.

Our findings reinforce the calls for (1) assessing all languages and collecting language background information (e.g., language use, premorbid language proficiency) of multilingual speakers with aphasia to obtain the most accurate assessment of their language abilities and (2) reporting performance in a way allowing researchers to compare the records among different studies, i.e., disclosing names of the assessment tools and scales used.

Growing understanding of the roles of such variables as premorbid language proficiency, language use and exposure, AoA, and structural similarities between one's languages will improve assessment practices and management options for multilingual speakers with aphasia. At the very least, multilingual speakers with aphasia should be assessed and treated with the understanding that it could be their earlier-acquired language that may be the key to greater success in restoring communication abilities.

## Data Availability

The dataset and R script used for the analysis are provided in Supplementary Material.

## Author Contributions

EK contributed to this study by designing the study concept, searching the databases, screening the data, coding the studies, planning and conducting the data analysis, interpreting the results, and writing the manuscript. MG contributed by designing the study concept, coding the studies, interpreting the results, writing the manuscript, and providing the external assistance for preparing the manuscript. MN contributed to the study concept and coded the studies. BW contributed by inspiring the very idea of the study, designing the study concept, interpreting the results, and writing the manuscript.

### Conflict of Interest Statement

The authors declare that the research was conducted in the absence of any commercial or financial relationships that could be construed as a potential conflict of interest.
