# Supplementary material for: What Influences Language Impairment in Bilingual Aphasia? A Meta-Analytic Review
Source: Front Psychol. 2019 Apr 4;10:445. doi: 10.3389/fpsyg.2019.00445 (PMC6460996; doi:10.3389/fpsyg.2019.00445)
Supplement: Supplementary file 2 [file Data_Sheet_2.docx]

Supplementary Material

**What influences language impairment in bilingual aphasia?**

**A meta-analytic review**

**Ekaterina Kuzmina*, Mira Goral, Monica Norvik, and Brendan S. Weekes**

*** Correspondence:** Ekaterina Kuzmina, [ekaterina.kuzmina@iln.uio.no](mailto:ekaterina.kuzmina@iln.uio.no)

***Database search strings***

1. PubMed: (aphasi* OR "language disorder" OR "language impairment" OR anomia OR stroke* OR vascular OR hemorrhage) AND (multiling* OR biling* OR triling* OR quadriling* OR polyglot) AND English[lang] AND "2001"[Date - Publication] : "2018"[Date - Publication] NOT child*[Title] (N = 260)
2. Science Direct: pub-date > 2000 and TITLE-ABSTR-KEY(aphasi* OR “language disorder” OR "language impairment" OR anomia OR stroke* OR vascular OR hemorrhage) and TITLE-ABSTR-KEY(multiling* OR biling* OR triling* OR quadriling* OR polyglot) and not TITLE-ABSTR-KEY(child*) (N = 67)
3. PsycINFO: (aphasi* or language disorder or language impairment or anom* or stroke* or vascular or hemorrhage).ab. or (aphasi* or language disorder or language impairment or anom* or stroke* or vascular or hemorrhage).ti. or (aphasi* or language disorder or language impairment or anom* or stroke* or vascular or hemorrhage).id. AND (multiling* or biling* or triling* or quadriling* or polyglot).ab. or (multiling* or biling* or triling* or quadriling* or polyglot).ti. or (multiling* or biling* or triling* or quadriling* or polyglot).id AND (bilingual not bilingual children).ab. AND (bilingual not bilingual children).ti. AND limit 5 to yr="2001 -Current” AND limit 6 to english language (N = 134)
4. CINAHL: (aphasi* OR language disorder OR language impairment OR anomia OR stroke OR vascular OR hemorrhage) AND (multiling* OR biling* OR triling* OR quadriling* OR polyglot) NOT child*  Limiters - Published Date: 20000101-20181231; Peer Reviewed; Language: English (N = 144)
5. TAYLOR & FRANCIS Online: [All: bilingual aphasia] AND [Publication Date: (01/01/2000 TO 12/31/2018) (N = 564)
