# Supplementary material for: What Influences Language Impairment in Bilingual Aphasia? A Meta-Analytic Review
Source: Front Psychol. 2019 Apr 4;10:445. doi: 10.3389/fpsyg.2019.00445 (PMC6460996; doi:10.3389/fpsyg.2019.00445)
Supplement: Supplementary file 3 [file Data_Sheet_3.pdf]

What influences language impairment in bilingual aphasia? A meta-analytic review

Ekaterina Kuzmina

19/03/2019

Setup

```r
#loading packages
library(ggplot2)
library(bitops)
library(metafor)
library(devtools)
library(Hmisc)

#loading data
rm(list=ls())
data<-read.csv("bilingual_aphasia_data.csv",header=TRUE)

#changing types of several variables
data$study_rq     <-as.factor(data$study_rq)
data$proficiency  <-as.factor(data$proficiency)
data$language_use <-as.factor(data$language_use)
data$case_mpo     <-as.numeric(data$case_mpo)
data$aoa_adj      <-as.numeric(data$aoa_adj)

#making all measure variables numeric
cols <- colnames(data[,40:153])
data[cols] <- sapply(data[cols],as.numeric)

#creating the 2-level linguistic distance variable
data$ling_similar_2levels<-as.factor(ifelse(data$ling_similarity>0,"similar","different"))

#creating the 3-level linguistic distance variable
data$ling_similar_3levels<-as.factor(ifelse(data$ling_similarity==0,"different",
                                     ifelse(data$ling_similarity==1,"close","very close")))
```

Correlational Analysis

L1 - Auditory comprehension total and Auditory comprehension testing paradigms

```
rcorr(as.matrix(data[,c("ac_commands_l1_cor_per","ac_l1_cor_per")]),type="spearman")
```

```
##                        ac_commands_l1_cor_per ac_l1_cor_per
## ac_commands_l1_cor_per                   1.00          0.86
## ac_l1_cor_per                            0.86          1.00
##
## n
##                        ac_commands_l1_cor_per ac_l1_cor_per
## ac_commands_l1_cor_per                     48            48
## ac_l1_cor_per                              48           100
##
## P
##                        ac_commands_l1_cor_per ac_l1_cor_per
## ac_commands_l1_cor_per                         0
## ac_l1_cor_per           0
```

```
rcorr(as.matrix(data[,c("ac_story_l1_cor_per","ac_l1_cor_per")]),type="spearman")
```

```
##                     ac_story_l1_cor_per ac_l1_cor_per
## ac_story_l1_cor_per                1.00          0.77
## ac_l1_cor_per                      0.77          1.00
##
## n
##                     ac_story_l1_cor_per ac_l1_cor_per
## ac_story_l1_cor_per                  23            23
## ac_l1_cor_per                        23           100
##
## P
##                     ac_story_l1_cor_per ac_l1_cor_per
## ac_story_l1_cor_per                      0
## ac_l1_cor_per        0
```

```
rcorr(as.matrix(data[,c("ac_picture_matching_l1_cor_per","ac_l1_cor_per")]),type="spearman")
```

```
##                                ac_picture_matching_l1_cor_per ac_l1_cor_per
## ac_picture_matching_l1_cor_per                           1.00          0.83
## ac_l1_cor_per                                            0.83          1.00
##
## n
##                                ac_picture_matching_l1_cor_per ac_l1_cor_per
## ac_picture_matching_l1_cor_per                             62            62
## ac_l1_cor_per                                              62           100
##
## P
##                                ac_picture_matching_l1_cor_per ac_l1_cor_per
## ac_picture_matching_l1_cor_per                                 0
## ac_l1_cor_per                   0
```

```r
rcorr(as.matrix(data[,c("ac_gram_judg_l1_cor_per","ac_l1_cor_per")]),type="spearman")
```

```
##                        ac_gram_judg_l1_cor_per ac_l1_cor_per
## ac_gram_judg_l1_cor_per                    1.00          0.85
## ac_l1_cor_per                              0.85          1.00
##
## n
##                        ac_gram_judg_l1_cor_per ac_l1_cor_per
## ac_gram_judg_l1_cor_per                      36            36
## ac_l1_cor_per                                36           100
##
## P
##                        ac_gram_judg_l1_cor_per ac_l1_cor_per
## ac_gram_judg_l1_cor_per                          0
## ac_l1_cor_per           0
```

```r
rcorr(as.matrix(data[,c("ac_lex_dec_l1_cor_per","ac_l1_cor_per")]),type="spearman")
```

```
##                      ac_lex_dec_l1_cor_per ac_l1_cor_per
## ac_lex_dec_l1_cor_per                  1.00          0.81
## ac_l1_cor_per                          0.81          1.00
##
## n
##                      ac_lex_dec_l1_cor_per ac_l1_cor_per
## ac_lex_dec_l1_cor_per                    38            38
## ac_l1_cor_per                            38           100
##
## P
##                      ac_lex_dec_l1_cor_per ac_l1_cor_per
## ac_lex_dec_l1_cor_per                        0
## ac_l1_cor_per         0
```

```r
rcorr(as.matrix(data[,c("ac_sem_rel_judg_l1_cor_per","ac_l1_cor_per")]),type="spearman")
```

```
##                           ac_sem_rel_judg_l1_cor_per ac_l1_cor_per
## ac_sem_rel_judg_l1_cor_per                       1.00          0.84
## ac_l1_cor_per                                    0.84          1.00
##
## n
##                           ac_sem_rel_judg_l1_cor_per ac_l1_cor_per
## ac_sem_rel_judg_l1_cor_per                         31            31
## ac_l1_cor_per                                      31           100
##
## P
##                           ac_sem_rel_judg_l1_cor_per ac_l1_cor_per
## ac_sem_rel_judg_l1_cor_per                            0
## ac_l1_cor_per              0
```

```r
rcorr(as.matrix(data[,c("ac_other_l1_cor_per","ac_l1_cor_per")]),type="spearman")
```

```
##                    ac_other_l1_cor_per ac_l1_cor_per
```

```
## ac_other_l1_cor_per                1.00         0.95
## ac_l1_cor_per                      0.95         1.00
##
## n
##                     ac_other_l1_cor_per ac_l1_cor_per
## ac_other_l1_cor_per                  21            21
## ac_l1_cor_per                        21           100
##
## P
##                     ac_other_l1_cor_per ac_l1_cor_per
## ac_other_l1_cor_per                     0
## ac_l1_cor_per        0
```

L1 - Oral production total and Auditory comprehension testing paradigms

```
rcorr(as.matrix(data[,c("ac_commands_l1_cor_per","op_l1_cor_per")]),type="spearman")
```

```
##                        ac_commands_l1_cor_per op_l1_cor_per
## ac_commands_l1_cor_per                   1.00          0.59
## op_l1_cor_per                            0.59          1.00
##
## n
##                        ac_commands_l1_cor_per op_l1_cor_per
## ac_commands_l1_cor_per                     48            48
## op_l1_cor_per                              48           113
##
## P
##                        ac_commands_l1_cor_per op_l1_cor_per
## ac_commands_l1_cor_per                         0
## op_l1_cor_per           0
```

```
rcorr(as.matrix(data[,c("ac_story_l1_cor_per","op_l1_cor_per")]),type="spearman")
```

```
##                     ac_story_l1_cor_per op_l1_cor_per
## ac_story_l1_cor_per                1.00          0.61
## op_l1_cor_per                      0.61          1.00
##
## n
##                     ac_story_l1_cor_per op_l1_cor_per
## ac_story_l1_cor_per                  23            23
## op_l1_cor_per                        23           113
##
## P
##                     ac_story_l1_cor_per op_l1_cor_per
## ac_story_l1_cor_per                     0.0021
## op_l1_cor_per       0.0021
```

```
rcorr(as.matrix(data[,c("ac_picture_matching_l1_cor_per","op_l1_cor_per")]),type="spearman")
```

```
##                                ac_picture_matching_l1_cor_per op_l1_cor_per
```

```
## ac_picture_matching_l1_cor_per                         1.00          0.55
## op_l1_cor_per                                           0.55          1.00
##
## n
##                                 ac_picture_matching_l1_cor_per op_l1_cor_per
## ac_picture_matching_l1_cor_per                             62            54
## op_l1_cor_per                                              54           113
##
## P
##                                 ac_picture_matching_l1_cor_per op_l1_cor_per
## ac_picture_matching_l1_cor_per                                  0
## op_l1_cor_per                    0
```

```
rcorr(as.matrix(data[,c("ac_gram_judg_l1_cor_per","op_l1_cor_per")]),type="spearman")
```

```
##                          ac_gram_judg_l1_cor_per op_l1_cor_per
## ac_gram_judg_l1_cor_per                    1.00          0.56
## op_l1_cor_per                              0.56          1.00
##
## n
##                          ac_gram_judg_l1_cor_per op_l1_cor_per
## ac_gram_judg_l1_cor_per                      36            27
## op_l1_cor_per                                27           113
##
## P
##                          ac_gram_judg_l1_cor_per op_l1_cor_per
## ac_gram_judg_l1_cor_per                          0.0022
## op_l1_cor_per            0.0022
```

```
rcorr(as.matrix(data[,c("ac_lex_dec_l1_cor_per","op_l1_cor_per")]),type="spearman")
```

```
##                        ac_lex_dec_l1_cor_per op_l1_cor_per
## ac_lex_dec_l1_cor_per                  1.00          0.67
## op_l1_cor_per                          0.67          1.00
##
## n
##                        ac_lex_dec_l1_cor_per op_l1_cor_per
## ac_lex_dec_l1_cor_per                    38            30
## op_l1_cor_per                            30           113
##
## P
##                        ac_lex_dec_l1_cor_per op_l1_cor_per
## ac_lex_dec_l1_cor_per                         0
## op_l1_cor_per           0
```

```
rcorr(as.matrix(data[,c("ac_sem_rel_judg_l1_cor_per","op_l1_cor_per")]),type="spearman")
```

```
##                             ac_sem_rel_judg_l1_cor_per op_l1_cor_per
## ac_sem_rel_judg_l1_cor_per                         1.0           0.7
## op_l1_cor_per                                      0.7           1.0
##
## n
```

```
##                            ac_sem_rel_judg_l1_cor_per op_l1_cor_per
## ac_sem_rel_judg_l1_cor_per                         31            31
## op_l1_cor_per                                      31           113
##
## P
##                            ac_sem_rel_judg_l1_cor_per op_l1_cor_per
## ac_sem_rel_judg_l1_cor_per                             0
## op_l1_cor_per               0
```

```
rcorr(as.matrix(data[,c("ac_other_l1_cor_per","op_l1_cor_per")]),type="spearman")
```

```
##                     ac_other_l1_cor_per op_l1_cor_per
## ac_other_l1_cor_per                1.00          0.71
## op_l1_cor_per                      0.71          1.00
##
## n
##                     ac_other_l1_cor_per op_l1_cor_per
## ac_other_l1_cor_per                  21            21
## op_l1_cor_per                        21           113
##
## P
##                     ac_other_l1_cor_per op_l1_cor_per
## ac_other_l1_cor_per                     3e-04
## op_l1_cor_per       3e-04
```

```
rcorr(as.matrix(data[,c("ac_l1_cor_per","op_l1_cor_per")]),type="spearman")
```

```
##               ac_l1_cor_per op_l1_cor_per
## ac_l1_cor_per          1.00          0.57
## op_l1_cor_per          0.57          1.00
##
## n
##               ac_l1_cor_per op_l1_cor_per
## ac_l1_cor_per           100            83
## op_l1_cor_per            83           113
##
## P
##               ac_l1_cor_per op_l1_cor_per
## ac_l1_cor_per                0
## op_l1_cor_per  0
```

L1 - Overall performance and Auditory comprehension testing paradigms

```
rcorr(as.matrix(data[,c("ac_commands_l1_cor_per","total_l1_cor_per")]),type="spearman")
```

```
##                        ac_commands_l1_cor_per total_l1_cor_per
## ac_commands_l1_cor_per                   1.00             0.72
## total_l1_cor_per                         0.72             1.00
##
## n
```

```
##                         ac_commands_l1_cor_per total_l1_cor_per
## ac_commands_l1_cor_per                     48               48
## total_l1_cor_per                           48              130
##
## P
##                         ac_commands_l1_cor_per total_l1_cor_per
## ac_commands_l1_cor_per                         0
## total_l1_cor_per        0
```

```
rcorr(as.matrix(data[,c("ac_story_l1_cor_per","total_l1_cor_per")]),type="spearman")
```

```
##                      ac_story_l1_cor_per total_l1_cor_per
## ac_story_l1_cor_per                 1.00             0.68
## total_l1_cor_per                    0.68             1.00
##
## n
##                      ac_story_l1_cor_per total_l1_cor_per
## ac_story_l1_cor_per                   23               23
## total_l1_cor_per                      23              130
##
## P
##                      ac_story_l1_cor_per total_l1_cor_per
## ac_story_l1_cor_per                      3e-04
## total_l1_cor_per     3e-04
```

```
rcorr(as.matrix(data[,c("ac_picture_matching_l1_cor_per","total_l1_cor_per")]),type="spearman")
```

```
##                                 ac_picture_matching_l1_cor_per total_l1_cor_per
## ac_picture_matching_l1_cor_per                            1.00             0.74
## total_l1_cor_per                                          0.74             1.00
##
## n
##                                 ac_picture_matching_l1_cor_per total_l1_cor_per
## ac_picture_matching_l1_cor_per                              62               62
## total_l1_cor_per                                            62              130
##
## P
##                                 ac_picture_matching_l1_cor_per total_l1_cor_per
## ac_picture_matching_l1_cor_per                                 0
## total_l1_cor_per                 0
```

```
rcorr(as.matrix(data[,c("ac_gram_judg_l1_cor_per","total_l1_cor_per")]),type="spearman")
```

```
##                          ac_gram_judg_l1_cor_per total_l1_cor_per
## ac_gram_judg_l1_cor_per                     1.00             0.74
## total_l1_cor_per                            0.74             1.00
##
## n
##                          ac_gram_judg_l1_cor_per total_l1_cor_per
## ac_gram_judg_l1_cor_per                       36               36
## total_l1_cor_per                              36              130
##
```

```
## P
##                          ac_gram_judg_l1_cor_per total_l1_cor_per
## ac_gram_judg_l1_cor_per                          0
## total_l1_cor_per        0
```

```r
rcorr(as.matrix(data[,c("ac_lex_dec_l1_cor_per","total_l1_cor_per")]),type="spearman")
```

```
##                        ac_lex_dec_l1_cor_per total_l1_cor_per
## ac_lex_dec_l1_cor_per                   1.00             0.78
## total_l1_cor_per                        0.78             1.00
##
## n
##                        ac_lex_dec_l1_cor_per total_l1_cor_per
## ac_lex_dec_l1_cor_per                     38               38
## total_l1_cor_per                          38              130
##
## P
##                        ac_lex_dec_l1_cor_per total_l1_cor_per
## ac_lex_dec_l1_cor_per                        0
## total_l1_cor_per       0
```

```r
rcorr(as.matrix(data[,c("ac_sem_rel_judg_l1_cor_per","total_l1_cor_per")]),type="spearman")
```

```
##                             ac_sem_rel_judg_l1_cor_per total_l1_cor_per
## ac_sem_rel_judg_l1_cor_per                        1.00             0.78
## total_l1_cor_per                                  0.78             1.00
##
## n
##                             ac_sem_rel_judg_l1_cor_per total_l1_cor_per
## ac_sem_rel_judg_l1_cor_per                          31               31
## total_l1_cor_per                                    31              130
##
## P
##                             ac_sem_rel_judg_l1_cor_per total_l1_cor_per
## ac_sem_rel_judg_l1_cor_per                             0
## total_l1_cor_per            0
```

```r
rcorr(as.matrix(data[,c("ac_other_l1_cor_per","total_l1_cor_per")]),type="spearman")
```

```
##                      ac_other_l1_cor_per total_l1_cor_per
## ac_other_l1_cor_per                 1.00             0.83
## total_l1_cor_per                    0.83             1.00
##
## n
##                      ac_other_l1_cor_per total_l1_cor_per
## ac_other_l1_cor_per                   21               21
## total_l1_cor_per                      21              130
##
## P
##                      ac_other_l1_cor_per total_l1_cor_per
## ac_other_l1_cor_per                      0
## total_l1_cor_per     0
```

```r
rcorr(as.matrix(data[,c("ac_l1_cor_per","total_l1_cor_per")]),type="spearman")
```

```
##                  ac_l1_cor_per total_l1_cor_per
## ac_l1_cor_per              1.0              0.8
## total_l1_cor_per           0.8              1.0
##
## n
##                  ac_l1_cor_per total_l1_cor_per
## ac_l1_cor_per              100              100
## total_l1_cor_per           100              130
##
## P
##                  ac_l1_cor_per total_l1_cor_per
## ac_l1_cor_per                   0
## total_l1_cor_per  0
```

L2 - Auditory comprehension total and Auditory comprehension testing paradigms

```r
rcorr(as.matrix(data[,c("ac_commands_l2_cor_per","ac_l2_cor_per")]), type="spearman")
```

```
##                        ac_commands_l2_cor_per ac_l2_cor_per
## ac_commands_l2_cor_per                   1.00          0.88
## ac_l2_cor_per                            0.88          1.00
##
## n
##                        ac_commands_l2_cor_per ac_l2_cor_per
## ac_commands_l2_cor_per                     48            48
## ac_l2_cor_per                              48           100
##
## P
##                        ac_commands_l2_cor_per ac_l2_cor_per
## ac_commands_l2_cor_per                         0
## ac_l2_cor_per           0
```

```r
rcorr(as.matrix(data[,c("ac_story_l2_cor_per","ac_l2_cor_per")]), type="spearman")
```

```
##                     ac_story_l2_cor_per ac_l2_cor_per
## ac_story_l2_cor_per                1.00          0.82
## ac_l2_cor_per                      0.82          1.00
##
## n
##                     ac_story_l2_cor_per ac_l2_cor_per
## ac_story_l2_cor_per                  23            23
## ac_l2_cor_per                        23           100
##
## P
##                     ac_story_l2_cor_per ac_l2_cor_per
## ac_story_l2_cor_per                      0
## ac_l2_cor_per        0
```

```r
rcorr(as.matrix(data[,c("ac_picture_matching_l2_cor_per","ac_l2_cor_per")]),type="spearman")
```

```
##                                 ac_picture_matching_l2_cor_per ac_l2_cor_per
## ac_picture_matching_l2_cor_per                           1.00          0.84
## ac_l2_cor_per                                            0.84          1.00
##
## n
##                                 ac_picture_matching_l2_cor_per ac_l2_cor_per
## ac_picture_matching_l2_cor_per                             62            62
## ac_l2_cor_per                                              62           100
##
## P
##                                 ac_picture_matching_l2_cor_per ac_l2_cor_per
## ac_picture_matching_l2_cor_per                                  0
## ac_l2_cor_per                    0
```

```r
rcorr(as.matrix(data[,c("ac_gram_judg_l2_cor_per","ac_l2_cor_per")]),type="spearman")
```

```
##                          ac_gram_judg_l2_cor_per ac_l2_cor_per
## ac_gram_judg_l2_cor_per                     1.00          0.86
## ac_l2_cor_per                               0.86          1.00
##
## n
##                          ac_gram_judg_l2_cor_per ac_l2_cor_per
## ac_gram_judg_l2_cor_per                       36            36
## ac_l2_cor_per                                 36           100
##
## P
##                          ac_gram_judg_l2_cor_per ac_l2_cor_per
## ac_gram_judg_l2_cor_per                          0
## ac_l2_cor_per             0
```

```r
rcorr(as.matrix(data[,c("ac_lex_dec_l2_cor_per","ac_l2_cor_per")]),type="spearman")
```

```
##                        ac_lex_dec_l2_cor_per ac_l2_cor_per
## ac_lex_dec_l2_cor_per                   1.00          0.85
## ac_l2_cor_per                           0.85          1.00
##
## n
##                        ac_lex_dec_l2_cor_per ac_l2_cor_per
## ac_lex_dec_l2_cor_per                     38            38
## ac_l2_cor_per                             38           100
##
## P
##                        ac_lex_dec_l2_cor_per ac_l2_cor_per
## ac_lex_dec_l2_cor_per                        0
## ac_l2_cor_per           0
```

```r
rcorr(as.matrix(data[,c("ac_sem_rel_judg_l2_cor_per","ac_l2_cor_per")]),type="spearman")
```

```
##                              ac_sem_rel_judg_l2_cor_per ac_l2_cor_per
```

```
## ac_sem_rel_judg_l2_cor_per                        1.00         0.87
## ac_l2_cor_per                                     0.87         1.00
##
## n
##                             ac_sem_rel_judg_l2_cor_per ac_l2_cor_per
## ac_sem_rel_judg_l2_cor_per                          31            31
## ac_l2_cor_per                                       31           100
##
## P
##                             ac_sem_rel_judg_l2_cor_per ac_l2_cor_per
## ac_sem_rel_judg_l2_cor_per                              0
## ac_l2_cor_per              0
```

```r
rcorr(as.matrix(data[,c("ac_other_l2_cor_per","ac_l2_cor_per")]),type="spearman")
```

```
##                      ac_other_l2_cor_per ac_l2_cor_per
## ac_other_l2_cor_per                 1.0           0.9
## ac_l2_cor_per                       0.9           1.0
##
## n
##                      ac_other_l2_cor_per ac_l2_cor_per
## ac_other_l2_cor_per                  21            21
## ac_l2_cor_per                        21           100
##
## P
##                      ac_other_l2_cor_per ac_l2_cor_per
## ac_other_l2_cor_per                       0
## ac_l2_cor_per        0
```

L2 - Oral production total and Auditory comprehension testing paradigms

```r
rcorr(as.matrix(data[,c("ac_commands_l2_cor_per","op_l2_cor_per")]),type="spearman")
```

```
##                         ac_commands_l2_cor_per op_l2_cor_per
## ac_commands_l2_cor_per                    1.0           0.6
## op_l2_cor_per                             0.6           1.0
##
## n
##                         ac_commands_l2_cor_per op_l2_cor_per
## ac_commands_l2_cor_per                     48            48
## op_l2_cor_per                              48           113
##
## P
##                         ac_commands_l2_cor_per op_l2_cor_per
## ac_commands_l2_cor_per                          0
## op_l2_cor_per           0
```

```r
rcorr(as.matrix(data[,c("ac_story_l2_cor_per","op_l2_cor_per")]),type="spearman")
```

```
##                      ac_story_l2_cor_per op_l2_cor_per
```

```
## ac_story_l2_cor_per                 1.00         0.75
## op_l2_cor_per                       0.75         1.00
##
## n
##                     ac_story_l2_cor_per op_l2_cor_per
## ac_story_l2_cor_per                  23            23
## op_l2_cor_per                        23           113
##
## P
##                     ac_story_l2_cor_per op_l2_cor_per
## ac_story_l2_cor_per                      0
## op_l2_cor_per        0
```

```
rcorr(as.matrix(data[,c("ac_picture_matching_l2_cor_per","op_l2_cor_per")]),type="spearman")
```

```
##                                ac_picture_matching_l2_cor_per op_l2_cor_per
## ac_picture_matching_l2_cor_per                           1.00          0.62
## op_l2_cor_per                                            0.62          1.00
##
## n
##                                ac_picture_matching_l2_cor_per op_l2_cor_per
## ac_picture_matching_l2_cor_per                             62            54
## op_l2_cor_per                                              54           113
##
## P
##                                ac_picture_matching_l2_cor_per op_l2_cor_per
## ac_picture_matching_l2_cor_per                                 0
## op_l2_cor_per                   0
```

```
rcorr(as.matrix(data[,c("ac_gram_judg_l2_cor_per","op_l2_cor_per")]),type="spearman")
```

```
##                         ac_gram_judg_l2_cor_per op_l2_cor_per
## ac_gram_judg_l2_cor_per                    1.00          0.72
## op_l2_cor_per                              0.72          1.00
##
## n
##                         ac_gram_judg_l2_cor_per op_l2_cor_per
## ac_gram_judg_l2_cor_per                      36            27
## op_l2_cor_per                                27           113
##
## P
##                         ac_gram_judg_l2_cor_per op_l2_cor_per
## ac_gram_judg_l2_cor_per                          0
## op_l2_cor_per            0
```

```
rcorr(as.matrix(data[,c("ac_lex_dec_l2_cor_per","op_l2_cor_per")]),type="spearman")
```

```
##                       ac_lex_dec_l2_cor_per op_l2_cor_per
## ac_lex_dec_l2_cor_per                  1.00          0.65
## op_l2_cor_per                          0.65          1.00
##
## n
```

```
##                         ac_lex_dec_l2_cor_per op_l2_cor_per
## ac_lex_dec_l2_cor_per                       38            30
## op_l2_cor_per                               30           113
##
## P
##                         ac_lex_dec_l2_cor_per op_l2_cor_per
## ac_lex_dec_l2_cor_per                         1e-04
## op_l2_cor_per         1e-04
```

```
rcorr(as.matrix(data[,c("ac_sem_rel_judg_l2_cor_per","op_l2_cor_per")]),type="spearman")
```

```
##                              ac_sem_rel_judg_l2_cor_per op_l2_cor_per
## ac_sem_rel_judg_l2_cor_per                         1.00          0.68
## op_l2_cor_per                                      0.68          1.00
##
## n
##                              ac_sem_rel_judg_l2_cor_per op_l2_cor_per
## ac_sem_rel_judg_l2_cor_per                           31            31
## op_l2_cor_per                                        31           113
##
## P
##                              ac_sem_rel_judg_l2_cor_per op_l2_cor_per
## ac_sem_rel_judg_l2_cor_per                                0
## op_l2_cor_per                 0
```

```
rcorr(as.matrix(data[,c("ac_other_l2_cor_per","op_l2_cor_per")]),type="spearman")
```

```
##                       ac_other_l2_cor_per op_l2_cor_per
## ac_other_l2_cor_per                  1.00          0.78
## op_l2_cor_per                        0.78          1.00
##
## n
##                       ac_other_l2_cor_per op_l2_cor_per
## ac_other_l2_cor_per                    21            21
## op_l2_cor_per                          21           113
##
## P
##                       ac_other_l2_cor_per op_l2_cor_per
## ac_other_l2_cor_per                         0
## op_l2_cor_per          0
```

```
rcorr(as.matrix(data[,c("ac_l2_cor_per","op_l2_cor_per")]),type="spearman")
```

```
##               ac_l2_cor_per op_l2_cor_per
## ac_l2_cor_per          1.00          0.63
## op_l2_cor_per          0.63          1.00
##
## n
##               ac_l2_cor_per op_l2_cor_per
## ac_l2_cor_per           100            83
## op_l2_cor_per            83           113
##
```

```
## P
##               ac_l2_cor_per op_l2_cor_per
## ac_l2_cor_per               0
## op_l2_cor_per  0
```

L2 - Overall performance and Auditory comprehension testing paradigms

```r
rcorr(as.matrix(data[,c("ac_commands_l2_cor_per","total_l2_cor_per")]),type="spearman")
```

```
##                        ac_commands_l2_cor_per total_l2_cor_per
## ac_commands_l2_cor_per                   1.00             0.75
## total_l2_cor_per                         0.75             1.00
##
## n
##                        ac_commands_l2_cor_per total_l2_cor_per
## ac_commands_l2_cor_per                     48               48
## total_l2_cor_per                           48              130
##
## P
##                        ac_commands_l2_cor_per total_l2_cor_per
## ac_commands_l2_cor_per                         0
## total_l2_cor_per        0
```

```r
rcorr(as.matrix(data[,c("ac_story_l2_cor_per","total_l2_cor_per")]),type="spearman")
```

```
##                     ac_story_l2_cor_per total_l2_cor_per
## ac_story_l2_cor_per                1.00             0.83
## total_l2_cor_per                   0.83             1.00
##
## n
##                     ac_story_l2_cor_per total_l2_cor_per
## ac_story_l2_cor_per                  23               23
## total_l2_cor_per                     23              130
##
## P
##                     ac_story_l2_cor_per total_l2_cor_per
## ac_story_l2_cor_per                      0
## total_l2_cor_per     0
```

```r
rcorr(as.matrix(data[,c("ac_picture_matching_l2_cor_per","total_l2_cor_per")]),type="spearman")
```

```
##                                ac_picture_matching_l2_cor_per total_l2_cor_per
## ac_picture_matching_l2_cor_per                           1.00             0.74
## total_l2_cor_per                                         0.74             1.00
##
## n
##                                ac_picture_matching_l2_cor_per total_l2_cor_per
## ac_picture_matching_l2_cor_per                             62               62
## total_l2_cor_per                                           62              130
##
```

```
## P
##                                  ac_picture_matching_l2_cor_per total_l2_cor_per
## ac_picture_matching_l2_cor_per                                  0
## total_l2_cor_per                 0
```

```
rcorr(as.matrix(data[,c("ac_gram_judg_l2_cor_per","total_l2_cor_per")]),type="spearman")
```

```
##                         ac_gram_judg_l2_cor_per total_l2_cor_per
## ac_gram_judg_l2_cor_per                     1.0              0.8
## total_l2_cor_per                            0.8              1.0
##
## n
##                         ac_gram_judg_l2_cor_per total_l2_cor_per
## ac_gram_judg_l2_cor_per                      36               36
## total_l2_cor_per                             36              130
##
## P
##                         ac_gram_judg_l2_cor_per total_l2_cor_per
## ac_gram_judg_l2_cor_per                          0
## total_l2_cor_per         0
```

```
rcorr(as.matrix(data[,c("ac_lex_dec_l2_cor_per","total_l2_cor_per")]),type="spearman")
```

```
##                       ac_lex_dec_l2_cor_per total_l2_cor_per
## ac_lex_dec_l2_cor_per                  1.00             0.79
## total_l2_cor_per                       0.79             1.00
##
## n
##                       ac_lex_dec_l2_cor_per total_l2_cor_per
## ac_lex_dec_l2_cor_per                    38               38
## total_l2_cor_per                         38              130
##
## P
##                       ac_lex_dec_l2_cor_per total_l2_cor_per
## ac_lex_dec_l2_cor_per                        0
## total_l2_cor_per       0
```

```
rcorr(as.matrix(data[,c("ac_sem_rel_judg_l2_cor_per","total_l2_cor_per")]),type="spearman")
```

```
##                            ac_sem_rel_judg_l2_cor_per total_l2_cor_per
## ac_sem_rel_judg_l2_cor_per                       1.00             0.81
## total_l2_cor_per                                 0.81             1.00
##
## n
##                            ac_sem_rel_judg_l2_cor_per total_l2_cor_per
## ac_sem_rel_judg_l2_cor_per                         31               31
## total_l2_cor_per                                   31              130
##
## P
##                            ac_sem_rel_judg_l2_cor_per total_l2_cor_per
## ac_sem_rel_judg_l2_cor_per                             0
## total_l2_cor_per            0
```

```r
rcorr(as.matrix(data[,c("ac_other_l2_cor_per","total_l2_cor_per")]),type="spearman")
```

```
##                     ac_other_l2_cor_per total_l2_cor_per
## ac_other_l2_cor_per                1.00             0.84
## total_l2_cor_per                   0.84             1.00
##
## n
##                     ac_other_l2_cor_per total_l2_cor_per
## ac_other_l2_cor_per                  21               21
## total_l2_cor_per                     21              130
##
## P
##                     ac_other_l2_cor_per total_l2_cor_per
## ac_other_l2_cor_per                      0
## total_l2_cor_per     0
```

```r
rcorr(as.matrix(data[,c("ac_l2_cor_per","total_l2_cor_per")]),type="spearman")
```

```
##                  ac_l2_cor_per total_l2_cor_per
## ac_l2_cor_per              1.0              0.8
## total_l2_cor_per           0.8              1.0
##
## n
##                  ac_l2_cor_per total_l2_cor_per
## ac_l2_cor_per              100              100
## total_l2_cor_per           100              130
##
## P
##                  ac_l2_cor_per total_l2_cor_per
## ac_l2_cor_per                  0
## total_l2_cor_per  0
```

L1 - Auditory comprehension total and Oral production testing paradigms

```r
rcorr(as.matrix(data[,c("op_naming_l1_cor_per","ac_l1_cor_per")]), type="spearman")
```

```
##                      op_naming_l1_cor_per ac_l1_cor_per
## op_naming_l1_cor_per                 1.00          0.49
## ac_l1_cor_per                        0.49          1.00
##
## n
##                      op_naming_l1_cor_per ac_l1_cor_per
## op_naming_l1_cor_per                  106            79
## ac_l1_cor_per                          79           100
##
## P
##                      op_naming_l1_cor_per ac_l1_cor_per
## op_naming_l1_cor_per                       0
## ac_l1_cor_per         0
```

```r
rcorr(as.matrix(data[,c("op_repetition_l1_cor_per","ac_l1_cor_per")]), type="spearman")
```

```
##                          op_repetition_l1_cor_per ac_l1_cor_per
## op_repetition_l1_cor_per                     1.00          0.47
## ac_l1_cor_per                                0.47          1.00
##
## n
##                          op_repetition_l1_cor_per ac_l1_cor_per
## op_repetition_l1_cor_per                       64            63
## ac_l1_cor_per                                  63           100
##
## P
##                          op_repetition_l1_cor_per ac_l1_cor_per
## op_repetition_l1_cor_per                          1e-04
## ac_l1_cor_per            1e-04
```

```r
rcorr(as.matrix(data[,c("op_ans_quest_sent_compl_l1_cor_per","ac_l1_cor_per")]),type="spearman")
```

```
##                                    op_ans_quest_sent_compl_l1_cor_per ac_l1_cor_per
## op_ans_quest_sent_compl_l1_cor_per                                1.0           0.3
## ac_l1_cor_per                                                     0.3           1.0
##
## n
##                                    op_ans_quest_sent_compl_l1_cor_per ac_l1_cor_per
## op_ans_quest_sent_compl_l1_cor_per                                 10             9
## ac_l1_cor_per                                                       9           100
##
## P
##                                    op_ans_quest_sent_compl_l1_cor_per ac_l1_cor_per
## op_ans_quest_sent_compl_l1_cor_per                                    0.4288
## ac_l1_cor_per                      0.4288
```

```r
rcorr(as.matrix(data[,c("op_sent_constr_l1_cor_per","ac_l1_cor_per")]),type="spearman")
```

```
##                           op_sent_constr_l1_cor_per ac_l1_cor_per
## op_sent_constr_l1_cor_per                      1.00          0.79
## ac_l1_cor_per                                  0.79          1.00
##
## n
##                           op_sent_constr_l1_cor_per ac_l1_cor_per
## op_sent_constr_l1_cor_per                        24            23
## ac_l1_cor_per                                    23           100
##
## P
##                           op_sent_constr_l1_cor_per ac_l1_cor_per
## op_sent_constr_l1_cor_per                            0
## ac_l1_cor_per              0
```

```r
rcorr(as.matrix(data[,c("op_semantic_opposites_l1_cor_per","ac_l1_cor_per")]),type="spearman")
```

```
##                                  op_semantic_opposites_l1_cor_per ac_l1_cor_per
```

```
## op_semantic_opposites_l1_cor_per                              1.00          0.82
## ac_l1_cor_per                                                  0.82          1.00
##
## n
##                                  op_semantic_opposites_l1_cor_per ac_l1_cor_per
## op_semantic_opposites_l1_cor_per                               25            23
## ac_l1_cor_per                                                  23           100
##
## P
##                                  op_semantic_opposites_l1_cor_per ac_l1_cor_per
## op_semantic_opposites_l1_cor_per                                   0
## ac_l1_cor_per                     0
```

```
rcorr(as.matrix(data[,c("op_morph_derivates_l1_cor_per","ac_l1_cor_per")]),type="spearman")
```

```
##                               op_morph_derivates_l1_cor_per ac_l1_cor_per
## op_morph_derivates_l1_cor_per                          1.00          0.87
## ac_l1_cor_per                                          0.87          1.00
##
## n
##                               op_morph_derivates_l1_cor_per ac_l1_cor_per
## op_morph_derivates_l1_cor_per                            15            15
## ac_l1_cor_per                                            15           100
##
## P
##                               op_morph_derivates_l1_cor_per ac_l1_cor_per
## op_morph_derivates_l1_cor_per                                0
## ac_l1_cor_per                  0
```

```
rcorr(as.matrix(data[,c("op_spont_semispont_l1_cor_per","ac_l1_cor_per")]),type="spearman")
```

```
##                               op_spont_semispont_l1_cor_per ac_l1_cor_per
## op_spont_semispont_l1_cor_per                          1.00          0.55
## ac_l1_cor_per                                          0.55          1.00
##
## n
##                               op_spont_semispont_l1_cor_per ac_l1_cor_per
## op_spont_semispont_l1_cor_per                            22            17
## ac_l1_cor_per                                            17           100
##
## P
##                               op_spont_semispont_l1_cor_per ac_l1_cor_per
## op_spont_semispont_l1_cor_per                               0.0221
## ac_l1_cor_per                 0.0221
```

L1 - Oral production total and Oral production testing paradigms

```
rcorr(as.matrix(data[,c("op_naming_l1_cor_per","op_l1_cor_per")]), type="spearman")
```

```
##                        op_naming_l1_cor_per op_l1_cor_per
```

```
## op_naming_l1_cor_per                  1.00         0.89
## op_l1_cor_per                         0.89         1.00
##
## n
##                       op_naming_l1_cor_per op_l1_cor_per
## op_naming_l1_cor_per                   106           106
## op_l1_cor_per                          106           113
##
## P
##                       op_naming_l1_cor_per op_l1_cor_per
## op_naming_l1_cor_per                       0
## op_l1_cor_per         0
```

```
rcorr(as.matrix(data[,c("op_repetition_l1_cor_per","op_l1_cor_per")]), type="spearman")
```

```
##                           op_repetition_l1_cor_per op_l1_cor_per
## op_repetition_l1_cor_per                      1.00          0.65
## op_l1_cor_per                                 0.65          1.00
##
## n
##                           op_repetition_l1_cor_per op_l1_cor_per
## op_repetition_l1_cor_per                        64            64
## op_l1_cor_per                                   64           113
##
## P
##                           op_repetition_l1_cor_per op_l1_cor_per
## op_repetition_l1_cor_per                               0
## op_l1_cor_per             0
```

```
rcorr(as.matrix(data[,c("op_ans_quest_sent_compl_l1_cor_per","op_l1_cor_per")]),type="spearman")
```

```
##                                     op_ans_quest_sent_compl_l1_cor_per op_l1_cor_per
## op_ans_quest_sent_compl_l1_cor_per                                1.00          0.45
## op_l1_cor_per                                                     0.45          1.00
##
## n
##                                     op_ans_quest_sent_compl_l1_cor_per op_l1_cor_per
## op_ans_quest_sent_compl_l1_cor_per                                  10            10
## op_l1_cor_per                                                       10           113
##
## P
##                                     op_ans_quest_sent_compl_l1_cor_per op_l1_cor_per
## op_ans_quest_sent_compl_l1_cor_per                                    0.1974
## op_l1_cor_per                       0.1974
```

```
rcorr(as.matrix(data[,c("op_sent_constr_l1_cor_per","op_l1_cor_per")]),type="spearman")
```

```
##                            op_sent_constr_l1_cor_per op_l1_cor_per
## op_sent_constr_l1_cor_per                       1.00          0.85
## op_l1_cor_per                                   0.85          1.00
##
## n
```

```
##                            op_sent_constr_l1_cor_per op_l1_cor_per
## op_sent_constr_l1_cor_per                        24            24
## op_l1_cor_per                                    24           113
##
## P
##                            op_sent_constr_l1_cor_per op_l1_cor_per
## op_sent_constr_l1_cor_per                                0
## op_l1_cor_per              0
```

```
rcorr(as.matrix(data[,c("op_semantic_opposites_l1_cor_per","op_l1_cor_per")]),type="spearman")
```

```
##                                   op_semantic_opposites_l1_cor_per op_l1_cor_per
## op_semantic_opposites_l1_cor_per                              1.00          0.88
## op_l1_cor_per                                                 0.88          1.00
##
## n
##                                   op_semantic_opposites_l1_cor_per op_l1_cor_per
## op_semantic_opposites_l1_cor_per                                25            25
## op_l1_cor_per                                                   25           113
##
## P
##                                   op_semantic_opposites_l1_cor_per op_l1_cor_per
## op_semantic_opposites_l1_cor_per                                    0
## op_l1_cor_per                     0
```

```
rcorr(as.matrix(data[,c("op_morph_derivates_l1_cor_per","op_l1_cor_per")]),type="spearman")
```

```
##                                op_morph_derivates_l1_cor_per op_l1_cor_per
## op_morph_derivates_l1_cor_per                           1.00          0.77
## op_l1_cor_per                                           0.77          1.00
##
## n
##                                op_morph_derivates_l1_cor_per op_l1_cor_per
## op_morph_derivates_l1_cor_per                             15            15
## op_l1_cor_per                                             15           113
##
## P
##                                op_morph_derivates_l1_cor_per op_l1_cor_per
## op_morph_derivates_l1_cor_per                               7e-04
## op_l1_cor_per                  7e-04
```

```
rcorr(as.matrix(data[,c("op_spont_semispont_l1_cor_per","op_l1_cor_per")]),type="spearman")
```

```
##                                op_spont_semispont_l1_cor_per op_l1_cor_per
## op_spont_semispont_l1_cor_per                           1.00          0.73
## op_l1_cor_per                                           0.73          1.00
##
## n
##                                op_spont_semispont_l1_cor_per op_l1_cor_per
## op_spont_semispont_l1_cor_per                             22            22
## op_l1_cor_per                                             22           113
##
```

```
## P
##                                op_spont_semispont_l1_cor_per op_l1_cor_per
## op_spont_semispont_l1_cor_per                               1e-04
## op_l1_cor_per                  1e-04
```

L1 - Overall performance and Oral production testing paradigms

```r
rcorr(as.matrix(data[,c("op_naming_l1_cor_per","total_l1_cor_per")]),type="spearman")
```

```
##                       op_naming_l1_cor_per total_l1_cor_per
## op_naming_l1_cor_per                  1.00             0.82
## total_l1_cor_per                      0.82             1.00
##
## n
##                       op_naming_l1_cor_per total_l1_cor_per
## op_naming_l1_cor_per                   106              106
## total_l1_cor_per                       106              130
##
## P
##                       op_naming_l1_cor_per total_l1_cor_per
## op_naming_l1_cor_per                        0
## total_l1_cor_per        0
```

```r
rcorr(as.matrix(data[,c("op_repetition_l1_cor_per","total_l1_cor_per")]),type="spearman")
```

```
##                           op_repetition_l1_cor_per total_l1_cor_per
## op_repetition_l1_cor_per                      1.00             0.61
## total_l1_cor_per                              0.61             1.00
##
## n
##                           op_repetition_l1_cor_per total_l1_cor_per
## op_repetition_l1_cor_per                        64               64
## total_l1_cor_per                                64              130
##
## P
##                           op_repetition_l1_cor_per total_l1_cor_per
## op_repetition_l1_cor_per                            0
## total_l1_cor_per            0
```

```r
rcorr(as.matrix(data[,c("op_ans_quest_sent_compl_l1_cor_per","total_l1_cor_per")]),type="spearman")
```

```
##                                     op_ans_quest_sent_compl_l1_cor_per total_l1_cor_per
## op_ans_quest_sent_compl_l1_cor_per                                1.00             0.48
## total_l1_cor_per                                                  0.48             1.00
##
## n
##                                     op_ans_quest_sent_compl_l1_cor_per total_l1_cor_per
## op_ans_quest_sent_compl_l1_cor_per                                  10               10
## total_l1_cor_per                                                    10              130
##
```

```
## P
##                                    op_ans_quest_sent_compl_l1_cor_per total_l1_cor_per
## op_ans_quest_sent_compl_l1_cor_per                                    0.1572
## total_l1_cor_per                   0.1572
```

```
rcorr(as.matrix(data[,c("op_sent_constr_l1_cor_per","total_l1_cor_per")]),type="spearman")
```

```
##                           op_sent_constr_l1_cor_per total_l1_cor_per
## op_sent_constr_l1_cor_per                       1.0              0.9
## total_l1_cor_per                                0.9              1.0
##
## n
##                           op_sent_constr_l1_cor_per total_l1_cor_per
## op_sent_constr_l1_cor_per                        24               24
## total_l1_cor_per                                 24              130
##
## P
##                           op_sent_constr_l1_cor_per total_l1_cor_per
## op_sent_constr_l1_cor_per                            0
## total_l1_cor_per           0
```

```
rcorr(as.matrix(data[,c("op_semantic_opposites_l1_cor_per","total_l1_cor_per")]),type="spearman")
```

```
##                                  op_semantic_opposites_l1_cor_per total_l1_cor_per
## op_semantic_opposites_l1_cor_per                             1.00             0.89
## total_l1_cor_per                                             0.89             1.00
##
## n
##                                  op_semantic_opposites_l1_cor_per total_l1_cor_per
## op_semantic_opposites_l1_cor_per                               25               25
## total_l1_cor_per                                               25              130
##
## P
##                                  op_semantic_opposites_l1_cor_per total_l1_cor_per
## op_semantic_opposites_l1_cor_per                                   0
## total_l1_cor_per                  0
```

```
rcorr(as.matrix(data[,c("op_morph_derivates_l1_cor_per","total_l1_cor_per")]),type="spearman")
```

```
##                               op_morph_derivates_l1_cor_per total_l1_cor_per
## op_morph_derivates_l1_cor_per                           1.0              0.8
## total_l1_cor_per                                        0.8              1.0
##
## n
##                               op_morph_derivates_l1_cor_per total_l1_cor_per
## op_morph_derivates_l1_cor_per                            15               15
## total_l1_cor_per                                         15              130
##
## P
##                               op_morph_derivates_l1_cor_per total_l1_cor_per
## op_morph_derivates_l1_cor_per                               3e-04
## total_l1_cor_per              3e-04
```

```r
rcorr(as.matrix(data[,c("op_spont_semispont_l1_cor_per","total_l1_cor_per")]),type="spearman")
```

```
##                              op_spont_semispont_l1_cor_per total_l1_cor_per
## op_spont_semispont_l1_cor_per                          1.0              0.7
## total_l1_cor_per                                       0.7              1.0
##
## n
##                              op_spont_semispont_l1_cor_per total_l1_cor_per
## op_spont_semispont_l1_cor_per                           22               22
## total_l1_cor_per                                        22              130
##
## P
##                              op_spont_semispont_l1_cor_per total_l1_cor_per
## op_spont_semispont_l1_cor_per                               3e-04
## total_l1_cor_per              3e-04
```

```r
rcorr(as.matrix(data[,c("op_l1_cor_per","total_l1_cor_per")]),type="spearman")
```

```
##                  op_l1_cor_per total_l1_cor_per
## op_l1_cor_per             1.00             0.93
## total_l1_cor_per          0.93             1.00
##
## n
##                  op_l1_cor_per total_l1_cor_per
## op_l1_cor_per              113              113
## total_l1_cor_per           113              130
##
## P
##                  op_l1_cor_per total_l1_cor_per
## op_l1_cor_per                   0
## total_l1_cor_per  0
```

L2 - Auditory comprehension total and Oral production testing paradigms

```r
rcorr(as.matrix(data[,c("op_naming_l2_cor_per","ac_l2_cor_per")]),type="spearman")
```

```
##                      op_naming_l2_cor_per ac_l2_cor_per
## op_naming_l2_cor_per                 1.00          0.59
## ac_l2_cor_per                        0.59          1.00
##
## n
##                      op_naming_l2_cor_per ac_l2_cor_per
## op_naming_l2_cor_per                  106            79
## ac_l2_cor_per                          79           100
##
## P
##                      op_naming_l2_cor_per ac_l2_cor_per
## op_naming_l2_cor_per                      0
## ac_l2_cor_per         0
```

```r
rcorr(as.matrix(data[,c("op_repetition_l2_cor_per","ac_l2_cor_per")]),type="spearman")
```

```
##                         op_repetition_l2_cor_per ac_l2_cor_per
## op_repetition_l2_cor_per                     1.00          0.59
## ac_l2_cor_per                                0.59          1.00
##
## n
##                         op_repetition_l2_cor_per ac_l2_cor_per
## op_repetition_l2_cor_per                       64            63
## ac_l2_cor_per                                  63           100
##
## P
##                         op_repetition_l2_cor_per ac_l2_cor_per
## op_repetition_l2_cor_per                          0
## ac_l2_cor_per            0
```

```r
rcorr(as.matrix(data[,c("op_ans_quest_sent_compl_l2_cor_per","ac_l2_cor_per")]),type="spearman")
```

```
##                                   op_ans_quest_sent_compl_l2_cor_per ac_l2_cor_per
## op_ans_quest_sent_compl_l2_cor_per                               1.00          0.46
## ac_l2_cor_per                                                    0.46          1.00
##
## n
##                                   op_ans_quest_sent_compl_l2_cor_per ac_l2_cor_per
## op_ans_quest_sent_compl_l2_cor_per                                 10             9
## ac_l2_cor_per                                                       9           100
##
## P
##                                   op_ans_quest_sent_compl_l2_cor_per ac_l2_cor_per
## op_ans_quest_sent_compl_l2_cor_per                                    0.2125
## ac_l2_cor_per                     0.2125
```

```r
rcorr(as.matrix(data[,c("op_sent_constr_l2_cor_per","ac_l2_cor_per")]),type="spearman")
```

```
##                          op_sent_constr_l2_cor_per ac_l2_cor_per
## op_sent_constr_l2_cor_per                      1.00          0.72
## ac_l2_cor_per                                  0.72          1.00
##
## n
##                          op_sent_constr_l2_cor_per ac_l2_cor_per
## op_sent_constr_l2_cor_per                        24            23
## ac_l2_cor_per                                    23           100
##
## P
##                          op_sent_constr_l2_cor_per ac_l2_cor_per
## op_sent_constr_l2_cor_per                           0
## ac_l2_cor_per             0
```

```r
rcorr(as.matrix(data[,c("op_semantic_opposites_l2_cor_per","ac_l2_cor_per")]),type="spearman")
```

```
##                                 op_semantic_opposites_l2_cor_per ac_l2_cor_per
```

```
## op_semantic_opposites_l2_cor_per                                1.00          0.81
## ac_l2_cor_per                                                   0.81          1.00
##
## n
##                                    op_semantic_opposites_l2_cor_per ac_l2_cor_per
## op_semantic_opposites_l2_cor_per                                  25            23
## ac_l2_cor_per                                                     23           100
##
## P
##                                    op_semantic_opposites_l2_cor_per ac_l2_cor_per
## op_semantic_opposites_l2_cor_per                                    0
## ac_l2_cor_per                       0
```

```
rcorr(as.matrix(data[,c("op_morph_derivates_l2_cor_per","ac_l2_cor_per")]),type="spearman")
```

```
##                                 op_morph_derivates_l2_cor_per ac_l2_cor_per
## op_morph_derivates_l2_cor_per                            1.00          0.88
## ac_l2_cor_per                                            0.88          1.00
##
## n
##                                 op_morph_derivates_l2_cor_per ac_l2_cor_per
## op_morph_derivates_l2_cor_per                              15            15
## ac_l2_cor_per                                              15           100
##
## P
##                                 op_morph_derivates_l2_cor_per ac_l2_cor_per
## op_morph_derivates_l2_cor_per                                 0
## ac_l2_cor_per                    0
```

```
rcorr(as.matrix(data[,c("op_spont_semispont_l2_cor_per","ac_l2_cor_per")]),type="spearman")
```

```
##                                 op_spont_semispont_l2_cor_per ac_l2_cor_per
## op_spont_semispont_l2_cor_per                            1.00          0.48
## ac_l2_cor_per                                            0.48          1.00
##
## n
##                                 op_spont_semispont_l2_cor_per ac_l2_cor_per
## op_spont_semispont_l2_cor_per                              22            17
## ac_l2_cor_per                                              17           100
##
## P
##                                 op_spont_semispont_l2_cor_per ac_l2_cor_per
## op_spont_semispont_l2_cor_per                               0.0503
## ac_l2_cor_per                   0.0503
```

L2 - Oral production total and Oral production testing paradigms

```
rcorr(as.matrix(data[,c("op_naming_l2_cor_per","op_l2_cor_per")]),type="spearman")
```

```
##                        op_naming_l2_cor_per op_l2_cor_per
```

```
## op_naming_l2_cor_per                    1.0          0.9
## op_l2_cor_per                           0.9          1.0
##
## n
##                       op_naming_l2_cor_per op_l2_cor_per
## op_naming_l2_cor_per                   106           106
## op_l2_cor_per                          106           113
##
## P
##                       op_naming_l2_cor_per op_l2_cor_per
## op_naming_l2_cor_per                       0
## op_l2_cor_per          0
```

```
rcorr(as.matrix(data[,c("op_repetition_l2_cor_per","op_l2_cor_per")]),type="spearman")
```

```
##                           op_repetition_l2_cor_per op_l2_cor_per
## op_repetition_l2_cor_per                     1.00          0.72
## op_l2_cor_per                                0.72          1.00
##
## n
##                           op_repetition_l2_cor_per op_l2_cor_per
## op_repetition_l2_cor_per                       64            64
## op_l2_cor_per                                  64           113
##
## P
##                           op_repetition_l2_cor_per op_l2_cor_per
## op_repetition_l2_cor_per                               0
## op_l2_cor_per              0
```

```
rcorr(as.matrix(data[,c("op_ans_quest_sent_compl_l2_cor_per","op_l2_cor_per")]),type="spearman")
```

```
##                                     op_ans_quest_sent_compl_l2_cor_per op_l2_cor_per
## op_ans_quest_sent_compl_l2_cor_per                                1.00          0.46
## op_l2_cor_per                                                     0.46          1.00
##
## n
##                                     op_ans_quest_sent_compl_l2_cor_per op_l2_cor_per
## op_ans_quest_sent_compl_l2_cor_per                                  10            10
## op_l2_cor_per                                                       10           113
##
## P
##                                     op_ans_quest_sent_compl_l2_cor_per op_l2_cor_per
## op_ans_quest_sent_compl_l2_cor_per                                    0.1789
## op_l2_cor_per                       0.1789
```

```
rcorr(as.matrix(data[,c("op_sent_constr_l2_cor_per","op_l2_cor_per")]),type="spearman")
```

```
##                            op_sent_constr_l2_cor_per op_l2_cor_per
## op_sent_constr_l2_cor_per                       1.00          0.86
## op_l2_cor_per                                   0.86          1.00
##
## n
```

```
##                            op_sent_constr_l2_cor_per op_l2_cor_per
## op_sent_constr_l2_cor_per                        24            24
## op_l2_cor_per                                    24           113
##
## P
##                            op_sent_constr_l2_cor_per op_l2_cor_per
## op_sent_constr_l2_cor_per                              0
## op_l2_cor_per              0
```

```
rcorr(as.matrix(data[,c("op_semantic_opposites_l2_cor_per","op_l2_cor_per")]),type="spearman")
```

```
##                                   op_semantic_opposites_l2_cor_per op_l2_cor_per
## op_semantic_opposites_l2_cor_per                             1.00          0.85
## op_l2_cor_per                                                0.85          1.00
##
## n
##                                   op_semantic_opposites_l2_cor_per op_l2_cor_per
## op_semantic_opposites_l2_cor_per                               25            25
## op_l2_cor_per                                                  25           113
##
## P
##                                   op_semantic_opposites_l2_cor_per op_l2_cor_per
## op_semantic_opposites_l2_cor_per                                     0
## op_l2_cor_per                     0
```

```
rcorr(as.matrix(data[,c("op_morph_derivates_l2_cor_per","op_l2_cor_per")]),type="spearman")
```

```
##                                op_morph_derivates_l2_cor_per op_l2_cor_per
## op_morph_derivates_l2_cor_per                          1.00          0.85
## op_l2_cor_per                                          0.85          1.00
##
## n
##                                op_morph_derivates_l2_cor_per op_l2_cor_per
## op_morph_derivates_l2_cor_per                            15            15
## op_l2_cor_per                                            15           113
##
## P
##                                op_morph_derivates_l2_cor_per op_l2_cor_per
## op_morph_derivates_l2_cor_per                                  0
## op_l2_cor_per                   0
```

```
rcorr(as.matrix(data[,c("op_spont_semispont_l2_cor_per","op_l2_cor_per")]),type="spearman")
```

```
##                                op_spont_semispont_l2_cor_per op_l2_cor_per
## op_spont_semispont_l2_cor_per                          1.00          0.74
## op_l2_cor_per                                          0.74          1.00
##
## n
##                                op_spont_semispont_l2_cor_per op_l2_cor_per
## op_spont_semispont_l2_cor_per                            22            22
## op_l2_cor_per                                            22           113
##
```

```
## P
##                               op_spont_semispont_l2_cor_per op_l2_cor_per
## op_spont_semispont_l2_cor_per                                0
## op_l2_cor_per                  0
```

L2 - Overall performance and Oral production testing paradigms

```
rcorr(as.matrix(data[,c("op_naming_l2_cor_per","total_l2_cor_per")]),type="spearman")
```

```
##                      op_naming_l2_cor_per total_l2_cor_per
## op_naming_l2_cor_per                 1.00             0.84
## total_l2_cor_per                     0.84             1.00
##
## n
##                      op_naming_l2_cor_per total_l2_cor_per
## op_naming_l2_cor_per                  106              106
## total_l2_cor_per                      106              130
##
## P
##                      op_naming_l2_cor_per total_l2_cor_per
## op_naming_l2_cor_per                       0
## total_l2_cor_per      0
```

```
rcorr(as.matrix(data[,c("op_repetition_l2_cor_per","total_l2_cor_per")]),type="spearman")
```

```
##                          op_repetition_l2_cor_per total_l2_cor_per
## op_repetition_l2_cor_per                     1.00             0.69
## total_l2_cor_per                             0.69             1.00
##
## n
##                          op_repetition_l2_cor_per total_l2_cor_per
## op_repetition_l2_cor_per                       64               64
## total_l2_cor_per                               64              130
##
## P
##                          op_repetition_l2_cor_per total_l2_cor_per
## op_repetition_l2_cor_per                           0
## total_l2_cor_per          0
```

```
rcorr(as.matrix(data[,c("op_ans_quest_sent_compl_l2_cor_per","total_l2_cor_per")]),type="spearman")
```

```
##                                    op_ans_quest_sent_compl_l2_cor_per total_l2_cor_per
## op_ans_quest_sent_compl_l2_cor_per                               1.00             0.39
## total_l2_cor_per                                                 0.39             1.00
##
## n
##                                    op_ans_quest_sent_compl_l2_cor_per total_l2_cor_per
## op_ans_quest_sent_compl_l2_cor_per                                 10               10
## total_l2_cor_per                                                   10              130
##
```

```
## P
##                                    op_ans_quest_sent_compl_l2_cor_per total_l2_cor_per
## op_ans_quest_sent_compl_l2_cor_per                                    0.2665
## total_l2_cor_per                   0.2665
```

```
rcorr(as.matrix(data[,c("op_sent_constr_l2_cor_per","total_l2_cor_per")]),type="spearman")
```

```
##                           op_sent_constr_l2_cor_per total_l2_cor_per
## op_sent_constr_l2_cor_per                      1.00             0.91
## total_l2_cor_per                               0.91             1.00
##
## n
##                           op_sent_constr_l2_cor_per total_l2_cor_per
## op_sent_constr_l2_cor_per                        24               24
## total_l2_cor_per                                 24              130
##
## P
##                           op_sent_constr_l2_cor_per total_l2_cor_per
## op_sent_constr_l2_cor_per                            0
## total_l2_cor_per           0
```

```
rcorr(as.matrix(data[,c("op_semantic_opposites_l2_cor_per","total_l2_cor_per")]),type="spearman")
```

```
##                                  op_semantic_opposites_l2_cor_per total_l2_cor_per
## op_semantic_opposites_l2_cor_per                              1.0              0.9
## total_l2_cor_per                                              0.9              1.0
##
## n
##                                  op_semantic_opposites_l2_cor_per total_l2_cor_per
## op_semantic_opposites_l2_cor_per                               25               25
## total_l2_cor_per                                               25              130
##
## P
##                                  op_semantic_opposites_l2_cor_per total_l2_cor_per
## op_semantic_opposites_l2_cor_per                                   0
## total_l2_cor_per                  0
```

```
rcorr(as.matrix(data[,c("op_morph_derivates_l2_cor_per","total_l2_cor_per")]),type="spearman")
```

```
##                               op_morph_derivates_l2_cor_per total_l2_cor_per
## op_morph_derivates_l2_cor_per                          1.00             0.88
## total_l2_cor_per                                       0.88             1.00
##
## n
##                               op_morph_derivates_l2_cor_per total_l2_cor_per
## op_morph_derivates_l2_cor_per                            15               15
## total_l2_cor_per                                         15              130
##
## P
##                               op_morph_derivates_l2_cor_per total_l2_cor_per
## op_morph_derivates_l2_cor_per                                0
## total_l2_cor_per               0
```

```r
rcorr(as.matrix(data[,c("op_spont_semispont_l2_cor_per","total_l2_cor_per")]),type="spearman")
```

```
##                              op_spont_semispont_l2_cor_per total_l2_cor_per
## op_spont_semispont_l2_cor_per                          1.00             0.69
## total_l2_cor_per                                       0.69             1.00
##
## n
##                              op_spont_semispont_l2_cor_per total_l2_cor_per
## op_spont_semispont_l2_cor_per                            22               22
## total_l2_cor_per                                         22              130
##
## P
##                              op_spont_semispont_l2_cor_per total_l2_cor_per
## op_spont_semispont_l2_cor_per                               4e-04
## total_l2_cor_per             4e-04
```

```r
rcorr(as.matrix(data[,c("op_l2_cor_per","total_l2_cor_per")]),type="spearman")
```

```
##                  op_l2_cor_per total_l2_cor_per
## op_l2_cor_per             1.00             0.94
## total_l2_cor_per          0.94             1.00
##
## n
##                  op_l2_cor_per total_l2_cor_per
## op_l2_cor_per              113              113
## total_l2_cor_per           113              130
##
## P
##                  op_l2_cor_per total_l2_cor_per
## op_l2_cor_per                   0
## total_l2_cor_per  0
```

L1 - Auditory comprehension total and Other paradigms and Other tests

```r
rcorr(as.matrix(data[,c("ra_reading_aloud_l1_cor_per","ac_l1_cor_per")]),type="spearman")
```

```
##                             ra_reading_aloud_l1_cor_per ac_l1_cor_per
## ra_reading_aloud_l1_cor_per                         1.0           0.4
## ac_l1_cor_per                                       0.4           1.0
##
## n
##                             ra_reading_aloud_l1_cor_per ac_l1_cor_per
## ra_reading_aloud_l1_cor_per                          41            41
## ac_l1_cor_per                                        41           100
##
## P
##                             ra_reading_aloud_l1_cor_per ac_l1_cor_per
## ra_reading_aloud_l1_cor_per                             0.0091
## ac_l1_cor_per               0.0091
```

```r
rcorr(as.matrix(data[,c("wc_l1_cor_per","ac_l1_cor_per")]),type="spearman")
```

```
##                wc_l1_cor_per ac_l1_cor_per
## wc_l1_cor_per           1.00          0.83
## ac_l1_cor_per           0.83          1.00
##
## n
##                wc_l1_cor_per ac_l1_cor_per
## wc_l1_cor_per             28            28
## ac_l1_cor_per             28           100
##
## P
##                wc_l1_cor_per ac_l1_cor_per
## wc_l1_cor_per                 0
## ac_l1_cor_per  0
```

```r
rcorr(as.matrix(data[,c("wp_l1_cor_per","ac_l1_cor_per")]),type="spearman")
```

```
##                wp_l1_cor_per ac_l1_cor_per
## wp_l1_cor_per           1.00          0.38
## ac_l1_cor_per           0.38          1.00
##
## n
##                wp_l1_cor_per ac_l1_cor_per
## wp_l1_cor_per             24            23
## ac_l1_cor_per             23           100
##
## P
##                wp_l1_cor_per ac_l1_cor_per
## wp_l1_cor_per               0.0747
## ac_l1_cor_per 0.0747
```

```r
rcorr(as.matrix(data[,c("other_l1_cor_per","ac_l1_cor_per")]),type="spearman")
```

```
##                   other_l1_cor_per ac_l1_cor_per
## other_l1_cor_per              1.00          0.43
## ac_l1_cor_per                 0.43          1.00
##
## n
##                   other_l1_cor_per ac_l1_cor_per
## other_l1_cor_per                27            27
## ac_l1_cor_per                   27           100
##
## P
##                   other_l1_cor_per ac_l1_cor_per
## other_l1_cor_per                  0.0235
## ac_l1_cor_per     0.0235
```

L1 - Oral production total and Other paradigms and Other tests

```r
rcorr(as.matrix(data[,c("ra_reading_aloud_l1_cor_per","op_l1_cor_per")]),type="spearman")
```

```
##                              ra_reading_aloud_l1_cor_per op_l1_cor_per
## ra_reading_aloud_l1_cor_per                         1.00          0.52
## op_l1_cor_per                                       0.52          1.00
##
## n
##                              ra_reading_aloud_l1_cor_per op_l1_cor_per
## ra_reading_aloud_l1_cor_per                           41            33
## op_l1_cor_per                                         33           113
##
## P
##                              ra_reading_aloud_l1_cor_per op_l1_cor_per
## ra_reading_aloud_l1_cor_per                              0.0019
## op_l1_cor_per                0.0019
```

```r
rcorr(as.matrix(data[,c("wc_l1_cor_per","op_l1_cor_per")]),type="spearman")
```

```
##               wc_l1_cor_per op_l1_cor_per
## wc_l1_cor_per          1.00          0.35
## op_l1_cor_per          0.35          1.00
##
## n
##               wc_l1_cor_per op_l1_cor_per
## wc_l1_cor_per            28            20
## op_l1_cor_per            20           113
##
## P
##               wc_l1_cor_per op_l1_cor_per
## wc_l1_cor_per               0.134
## op_l1_cor_per 0.134
```

```r
rcorr(as.matrix(data[,c("wp_l1_cor_per","op_l1_cor_per")]),type="spearman")
```

```
##               wp_l1_cor_per op_l1_cor_per
## wp_l1_cor_per          1.00          0.53
## op_l1_cor_per          0.53          1.00
##
## n
##               wp_l1_cor_per op_l1_cor_per
## wp_l1_cor_per            24            24
## op_l1_cor_per            24           113
##
## P
##               wp_l1_cor_per op_l1_cor_per
## wp_l1_cor_per               0.0081
## op_l1_cor_per 0.0081
```

```r
rcorr(as.matrix(data[,c("other_l1_cor_per","op_l1_cor_per")]),type="spearman")
```

```
##                   other_l1_cor_per op_l1_cor_per
## other_l1_cor_per             1.00          0.75
## op_l1_cor_per                0.75          1.00
##
## n
##                   other_l1_cor_per op_l1_cor_per
## other_l1_cor_per               27            19
## op_l1_cor_per                  19           113
##
## P
##                   other_l1_cor_per op_l1_cor_per
## other_l1_cor_per                   2e-04
## op_l1_cor_per     2e-04
```

L1 - Overall performance and Other paradigms and Other tests

```
rcorr(as.matrix(data[,c("ra_reading_aloud_l1_cor_per","total_l1_cor_per")]),type="spearman")
```

```
##                              ra_reading_aloud_l1_cor_per total_l1_cor_per
## ra_reading_aloud_l1_cor_per                         1.00             0.65
## total_l1_cor_per                                    0.65             1.00
##
## n
##                              ra_reading_aloud_l1_cor_per total_l1_cor_per
## ra_reading_aloud_l1_cor_per                           41               41
## total_l1_cor_per                                      41              130
##
## P
##                              ra_reading_aloud_l1_cor_per total_l1_cor_per
## ra_reading_aloud_l1_cor_per                                    0
## total_l1_cor_per              0
```

```
rcorr(as.matrix(data[,c("wc_l1_cor_per","total_l1_cor_per")]),type="spearman")
```

```
##                   wc_l1_cor_per total_l1_cor_per
## wc_l1_cor_per              1.00             0.78
## total_l1_cor_per           0.78             1.00
##
## n
##                   wc_l1_cor_per total_l1_cor_per
## wc_l1_cor_per                28               28
## total_l1_cor_per             28              130
##
## P
##                   wc_l1_cor_per total_l1_cor_per
## wc_l1_cor_per                    0
## total_l1_cor_per  0
```

```
rcorr(as.matrix(data[,c("wp_l1_cor_per","total_l1_cor_per")]),type="spearman")
```

```
##                  wp_l1_cor_per total_l1_cor_per
## wp_l1_cor_per             1.00             0.73
## total_l1_cor_per          0.73             1.00
##
## n
##                  wp_l1_cor_per total_l1_cor_per
## wp_l1_cor_per               24               24
## total_l1_cor_per            24              130
##
## P
##                  wp_l1_cor_per total_l1_cor_per
## wp_l1_cor_per                   0
## total_l1_cor_per  0
```

```
rcorr(as.matrix(data[,c("other_l1_cor_per","total_l1_cor_per")]),type="spearman")
```

```
##                  other_l1_cor_per total_l1_cor_per
## other_l1_cor_per             1.00             0.88
## total_l1_cor_per             0.88             1.00
##
## n
##                  other_l1_cor_per total_l1_cor_per
## other_l1_cor_per               27               27
## total_l1_cor_per               27              130
##
## P
##                  other_l1_cor_per total_l1_cor_per
## other_l1_cor_per                   0
## total_l1_cor_per  0
```

L2 - Auditory comprehension total and Other paradigms and Other tests

```
rcorr(as.matrix(data[,c("ra_reading_aloud_l2_cor_per","ac_l2_cor_per")]),type="spearman")
```

```
##                             ra_reading_aloud_l2_cor_per ac_l2_cor_per
## ra_reading_aloud_l2_cor_per                        1.00          0.35
## ac_l2_cor_per                                      0.35          1.00
##
## n
##                             ra_reading_aloud_l2_cor_per ac_l2_cor_per
## ra_reading_aloud_l2_cor_per                          41            41
## ac_l2_cor_per                                        41           100
##
## P
##                             ra_reading_aloud_l2_cor_per ac_l2_cor_per
## ra_reading_aloud_l2_cor_per                             0.0248
## ac_l2_cor_per               0.0248
```

```
rcorr(as.matrix(data[,c("wc_l2_cor_per","ac_l2_cor_per")]),type="spearman")
```

```
##                wc_l2_cor_per ac_l2_cor_per
## wc_l2_cor_per          1.00          0.61
## ac_l2_cor_per          0.61          1.00
##
## n
##                wc_l2_cor_per ac_l2_cor_per
## wc_l2_cor_per            28            28
## ac_l2_cor_per            28           100
##
## P
##                wc_l2_cor_per ac_l2_cor_per
## wc_l2_cor_per               6e-04
## ac_l2_cor_per 6e-04
```

```
rcorr(as.matrix(data[,c("wp_l2_cor_per","ac_l2_cor_per")]),type="spearman")
```

```
##                wp_l2_cor_per ac_l2_cor_per
## wp_l2_cor_per           1.0           0.5
## ac_l2_cor_per           0.5           1.0
##
## n
##                wp_l2_cor_per ac_l2_cor_per
## wp_l2_cor_per            24            23
## ac_l2_cor_per            23           100
##
## P
##                wp_l2_cor_per ac_l2_cor_per
## wp_l2_cor_per               0.0161
## ac_l2_cor_per 0.0161
```

```
rcorr(as.matrix(data[,c("other_l2_cor_per","ac_l2_cor_per")]),type="spearman")
```

```
##                   other_l2_cor_per ac_l2_cor_per
## other_l2_cor_per              1.0           0.3
## ac_l2_cor_per                 0.3           1.0
##
## n
##                   other_l2_cor_per ac_l2_cor_per
## other_l2_cor_per               27            27
## ac_l2_cor_per                  27           100
##
## P
##                   other_l2_cor_per ac_l2_cor_per
## other_l2_cor_per                  0.1251
## ac_l2_cor_per     0.1251
```

L2 - Oral production total and Other paradigms and Other tests

```
rcorr(as.matrix(data[,c("ra_reading_aloud_l2_cor_per","op_l2_cor_per")]),type="spearman")
```

```
##                             ra_reading_aloud_l2_cor_per op_l2_cor_per
## ra_reading_aloud_l2_cor_per                         1.0           0.3
## op_l2_cor_per                                       0.3           1.0
##
## n
##                             ra_reading_aloud_l2_cor_per op_l2_cor_per
## ra_reading_aloud_l2_cor_per                          41            33
## op_l2_cor_per                                        33           113
##
## P
##                             ra_reading_aloud_l2_cor_per op_l2_cor_per
## ra_reading_aloud_l2_cor_per                             0.0927
## op_l2_cor_per               0.0927
```

```
rcorr(as.matrix(data[,c("wc_l2_cor_per","op_l2_cor_per")]),type="spearman")
```

```
##               wc_l2_cor_per op_l2_cor_per
## wc_l2_cor_per          1.00          0.32
## op_l2_cor_per          0.32          1.00
##
## n
##               wc_l2_cor_per op_l2_cor_per
## wc_l2_cor_per            28            20
## op_l2_cor_per            20           113
##
## P
##               wc_l2_cor_per op_l2_cor_per
## wc_l2_cor_per               0.1646
## op_l2_cor_per 0.1646
```

```
rcorr(as.matrix(data[,c("wp_l2_cor_per","op_l2_cor_per")]),type="spearman")
```

```
##               wp_l2_cor_per op_l2_cor_per
## wp_l2_cor_per          1.00          0.56
## op_l2_cor_per          0.56          1.00
##
## n
##               wp_l2_cor_per op_l2_cor_per
## wp_l2_cor_per            24            24
## op_l2_cor_per            24           113
##
## P
##               wp_l2_cor_per op_l2_cor_per
## wp_l2_cor_per               0.0044
## op_l2_cor_per 0.0044
```

```
rcorr(as.matrix(data[,c("other_l2_cor_per","op_l2_cor_per")]),type="spearman")
```

```
##                  other_l2_cor_per op_l2_cor_per
## other_l2_cor_per             1.00          0.52
## op_l2_cor_per                0.52          1.00
##
```

```
## n
##                  other_l2_cor_per op_l2_cor_per
## other_l2_cor_per               27            19
## op_l2_cor_per                  19           113
##
## P
##                  other_l2_cor_per op_l2_cor_per
## other_l2_cor_per                  0.0235
## op_l2_cor_per    0.0235
```

L2 - Overall performance and Other paradigms and Other tests

```r
rcorr(as.matrix(data[,c("ra_reading_aloud_l2_cor_per","total_l2_cor_per")]),type="spearman")
```

```
##                              ra_reading_aloud_l2_cor_per total_l2_cor_per
## ra_reading_aloud_l2_cor_per                         1.00             0.55
## total_l2_cor_per                                    0.55             1.00
##
## n
##                              ra_reading_aloud_l2_cor_per total_l2_cor_per
## ra_reading_aloud_l2_cor_per                           41               41
## total_l2_cor_per                                      41              130
##
## P
##                              ra_reading_aloud_l2_cor_per total_l2_cor_per
## ra_reading_aloud_l2_cor_per                              2e-04
## total_l2_cor_per             2e-04
```

```r
rcorr(as.matrix(data[,c("wc_l2_cor_per","total_l2_cor_per")]),type="spearman")
```

```
##                  wc_l2_cor_per total_l2_cor_per
## wc_l2_cor_per             1.00             0.71
## total_l2_cor_per          0.71             1.00
##
## n
##                  wc_l2_cor_per total_l2_cor_per
## wc_l2_cor_per               28               28
## total_l2_cor_per            28              130
##
## P
##                  wc_l2_cor_per total_l2_cor_per
## wc_l2_cor_per                   0
## total_l2_cor_per  0
```

```r
rcorr(as.matrix(data[,c("wp_l2_cor_per","total_l2_cor_per")]),type="spearman")
```

```
##                  wp_l2_cor_per total_l2_cor_per
## wp_l2_cor_per             1.00             0.69
## total_l2_cor_per          0.69             1.00
##
```

```
## n
##                   wp_l2_cor_per total_l2_cor_per
## wp_l2_cor_per                24               24
## total_l2_cor_per             24              130
##
## P
##                   wp_l2_cor_per total_l2_cor_per
## wp_l2_cor_per                   2e-04
## total_l2_cor_per 2e-04
```

```r
rcorr(as.matrix(data[,c("other_l2_cor_per","total_l2_cor_per")]),type="spearman")
```

```
##                   other_l2_cor_per total_l2_cor_per
## other_l2_cor_per              1.00             0.72
## total_l2_cor_per              0.72             1.00
##
## n
##                   other_l2_cor_per total_l2_cor_per
## other_l2_cor_per                27               27
## total_l2_cor_per                27              130
##
## P
##                   other_l2_cor_per total_l2_cor_per
## other_l2_cor_per                    0
## total_l2_cor_per  0
```

Data Screening

```
#removing simultaneous bilinguals from the dataset
data<-data[!(data$early_or_late == "simult"),]
```

Overall Performance

```
data<-escalc(measure="RR",
             ai=total_l1_cor,bi=total_l1_wrong,ci=total_l2_cor,di=total_l2_wrong,
             data=data,append=TRUE)

data<-data[!is.na(data$yi),]

overall<-rma(yi,vi,data=data)

summary(data$yi)
```

```
##     Min.  1st Qu.   Median     Mean  3rd Qu.     Max.
## -1.30174 -0.02269  0.10480  0.11684  0.23937  2.19722
```

```
sum(!is.na(data$yi))
```

```
## [1] 124
```

Auditory Comprehension

```
data_ac<-escalc(measure="RR",
                ai=ac_l1_cor,bi=ac_l1_wrong,ci=ac_l2_cor,di=ac_l2_wrong,
                data=data,append=TRUE)

data_ac<-data_ac[!is.na(data_ac$yi),]

ac<-rma(yi,vi,data=data_ac)

summary(data_ac$yi)
```

```
##     Min.  1st Qu.   Median     Mean  3rd Qu.     Max.
## -0.49248 -0.03175  0.04779  0.08785  0.20096  0.82869
```

```
sum(!is.na(data_ac$yi))
```

```
## [1] 96
```

Oral Production

```r
data_op<-escalc(measure="RR",
                  ai=op_l1_cor,bi=op_l1_wrong,ci=op_l2_cor,di=op_l2_wrong,
                  data=data,append=TRUE)

data_op<-data_op[!is.na(data_op$yi),]

op<-rma(yi,vi,data=data_op)

summary(data_op$yi)
```

```
##     Min.  1st Qu.   Median     Mean  3rd Qu.     Max.
## -2.06369 -0.05882  0.12260  0.09107  0.31920  2.19722
```

```r
sum(!is.na(data_op$yi))
```

```
## [1] 107
```

```r
#creating funnel plots
par(mfrow=c(1,3))
funnel_overall<-funnel(overall,
                      refline=0,level=c(90,95,99),shade=c("white","red","orange"),
                      ylab="Overall Performance",xlab="Risk Ratio (log)",
                      ylim=c(1.2,0),xlim=c(-2.5,2.5),steps=13,digits=c(1,1),
                      pch=1,cex.axis=0.5,cex.lab=0.8,cex.sub=0.8)
funnel_ac<-funnel(ac,
                      refline=0,level=c(90,95,99),shade=c("white","red","orange"),
                      ylab="Auditory Comprehension",xlab="Risk Ratio (log)",
                      ylim=c(1.2,0),xlim=c(-2.5,2.5),steps=13,digits=c(1,1),
                      pch=6,cex.axis=0.5,cex.lab=0.8,cex.sub=0.8)
funnel_op<-funnel(op,
                      refline=0,level=c(90,95,99),shade=c("white","red","orange"),
                      ylab="Oral Production",xlab="Risk Ratio (log)",
                      ylim=c(1.2,0),xlim=c(-2.5,2.5),steps=13,digits=c(1,1),
                      pch=5,cex.axis=0.5,cex.lab=0.8,cex.sub=0.8)
```

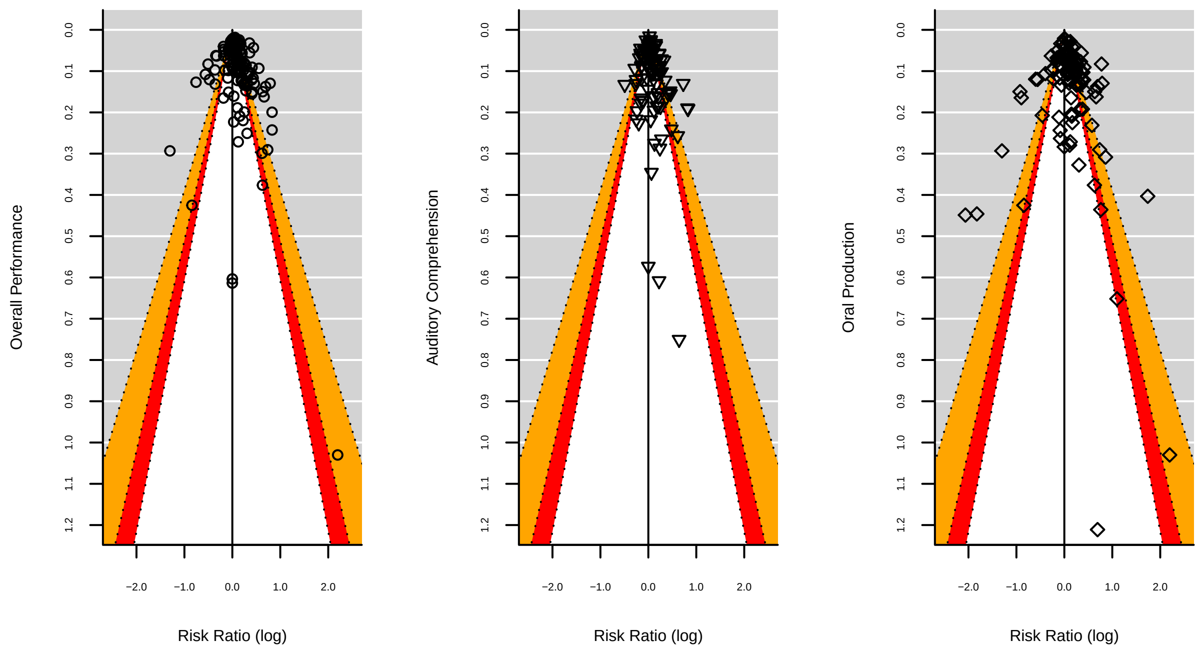

```
par(mfrow=c(3,1))
hist(data$yi,breaks=seq(-2.5,2.5,by=0.10),main=NULL)
hist(data_ac$yi,breaks=seq(-2.5,2.5,by=0.10),main=NULL)
hist(data_op$yi,breaks=seq(-2.5,2.5,by=0.10),main=NULL)
```

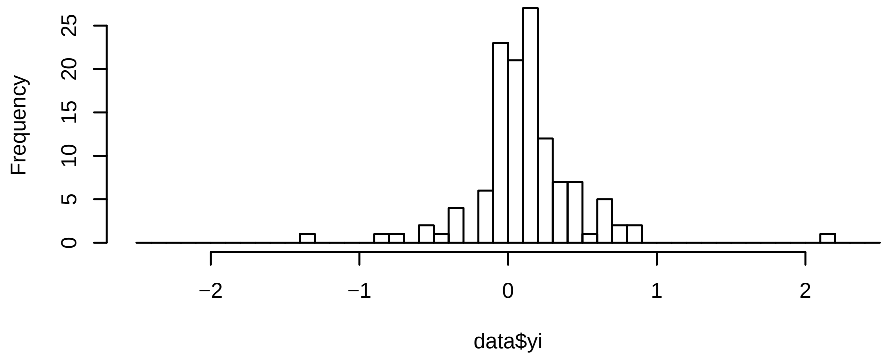

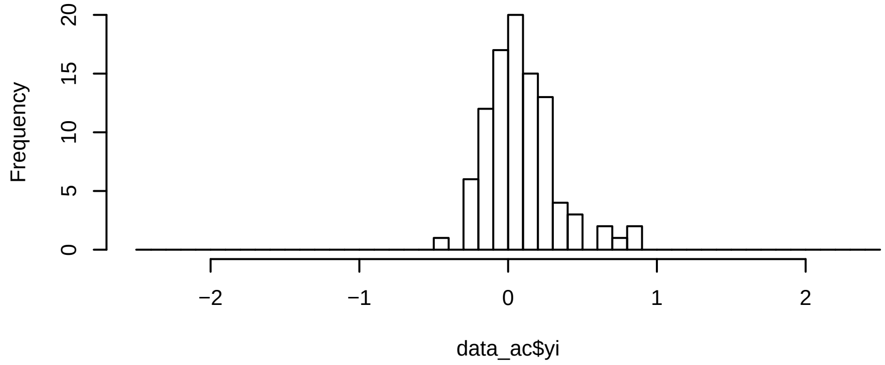

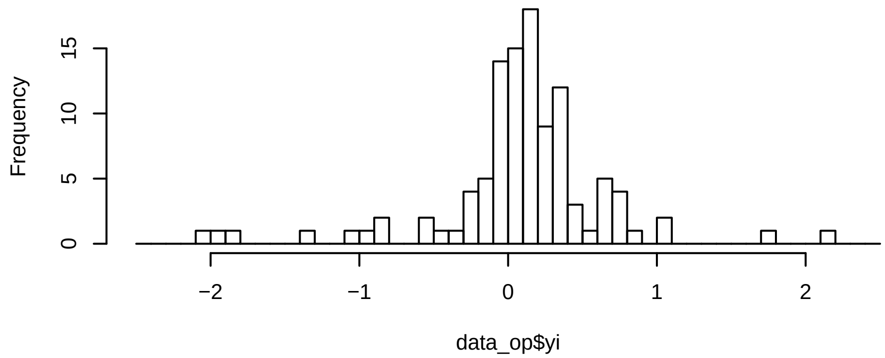

Data Trimming (deleting cases with SE > 0.3)

Overall Performance

```r
data=data[data$vi <= 0.09,]


summary(data$yi)
```

```
##     Min.  1st Qu.   Median     Mean  3rd Qu.     Max.
## -1.30174 -0.02316  0.10536  0.10509  0.23571  0.82883
```

```r
sum(!is.na(data$yi))
```

```
## [1] 119
```

Auditory Comprehension

```r
data_ac=data_ac[data_ac$vi <= 0.09,]

summary(data_ac$yi)
```

```
##     Min.  1st Qu.   Median     Mean  3rd Qu.     Max.
## -0.49248 -0.04139  0.04679  0.08246  0.19150  0.82869
```

```r
sum(!is.na(data_ac$yi))
```

```
## [1] 91
```

Oral Production

```r
data_op=data_op[data_op$vi <= 0.09,]

summary(data_op$yi)
```

```
##     Min.  1st Qu.   Median     Mean  3rd Qu.     Max.
## -1.30174 -0.05101  0.11653  0.08941  0.26400  0.78856
```

```r
sum(!is.na(data_op$yi))
```

```
## [1] 91
```

```r
par(mfrow=c(3,1))
hist(data$yi,breaks=seq(-2.5,2.5,by=0.10),main=NULL)
hist(data_ac$yi,breaks=seq(-2.5,2.5,by=0.10),main=NULL)
hist(data_op$yi,breaks=seq(-2.5,2.5,by=0.10),main=NULL)
```

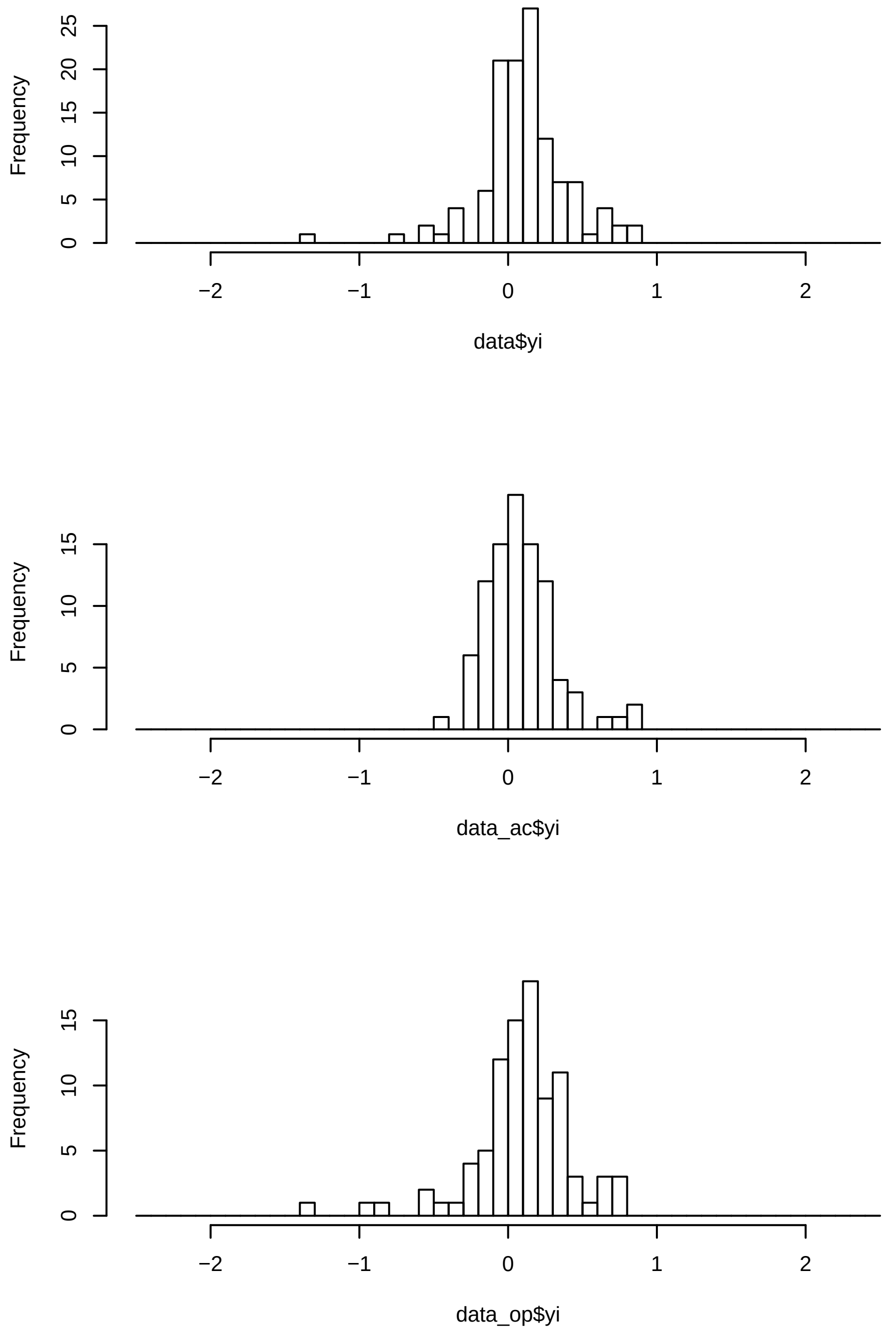

Language status: Difference between L1 and L2

Overall Performance

```
data<-data[order(data=data$early_or_late, data=data$yi),]
print(m1<-rma(yi,vi,data=data))
```

```
##
## Random-Effects Model (k = 119; tau^2 estimator: REML)
##
## tau^2 (estimated amount of total heterogeneity): 0.0517 (SE = 0.0079)
## tau (square root of estimated tau^2 value):      0.2275
## I^2 (total heterogeneity / total variability):   94.91%
## H^2 (total variability / sampling variability):  19.64
##
## Test for Heterogeneity:
## Q(df = 118) = 1025.1447, p-val < .0001
##
## Model Results:
##
## estimate      se    zval    pval   ci.lb   ci.ub
##   0.0929  0.0228  4.0839  <.0001  0.0483  0.1376  ***
##
## ---
## Signif. codes:  0 '***' 0.001 '**' 0.01 '*' 0.05 '.' 0.1 ' ' 1
```

```
#RR estimate,lower and upper boundaries of the 95% CI
round(exp(c(m1$b,m1$ci.lb,m1$ci.ub)),digits=2)
```

```
## [1] 1.10 1.05 1.15
```

Forestplot for Overall Performance for the whole group

```
forest(m1,showweights=TRUE,
       slab = paste(data$study_forestplot),
       xlim = c(-6, 2.5), cex = 1,font = 1,
       at = log(c(0.15, 0.2, 0.3, 0.5, 1, 1.5, 2, 2.5, 3, 3.5, 4)),
       atransf = exp,
       ilab =  data.frame(as.character(data$early_or_late),
                          as.character(data$proficiency),
                          as.character(data$language_use),
                          as.character(data$ling_similar_2levels)),
       ilab.xpos = c(-4.5,-3.75,-3,-2.25))

op <- par(cex=1.2,font=2)
text(c(-4.5,-3.75,-3,-2.25),121,c("AoA subgroup",
                                   "Proficiency",
                                   "Language use",
                                   "Linguistic similarity"))
text(-6, 121,"Cases",pos=4)
text(2.5, 121,"RR [95% CI]", pos=2)
text(2, 121,"Weight", pos=2)
```

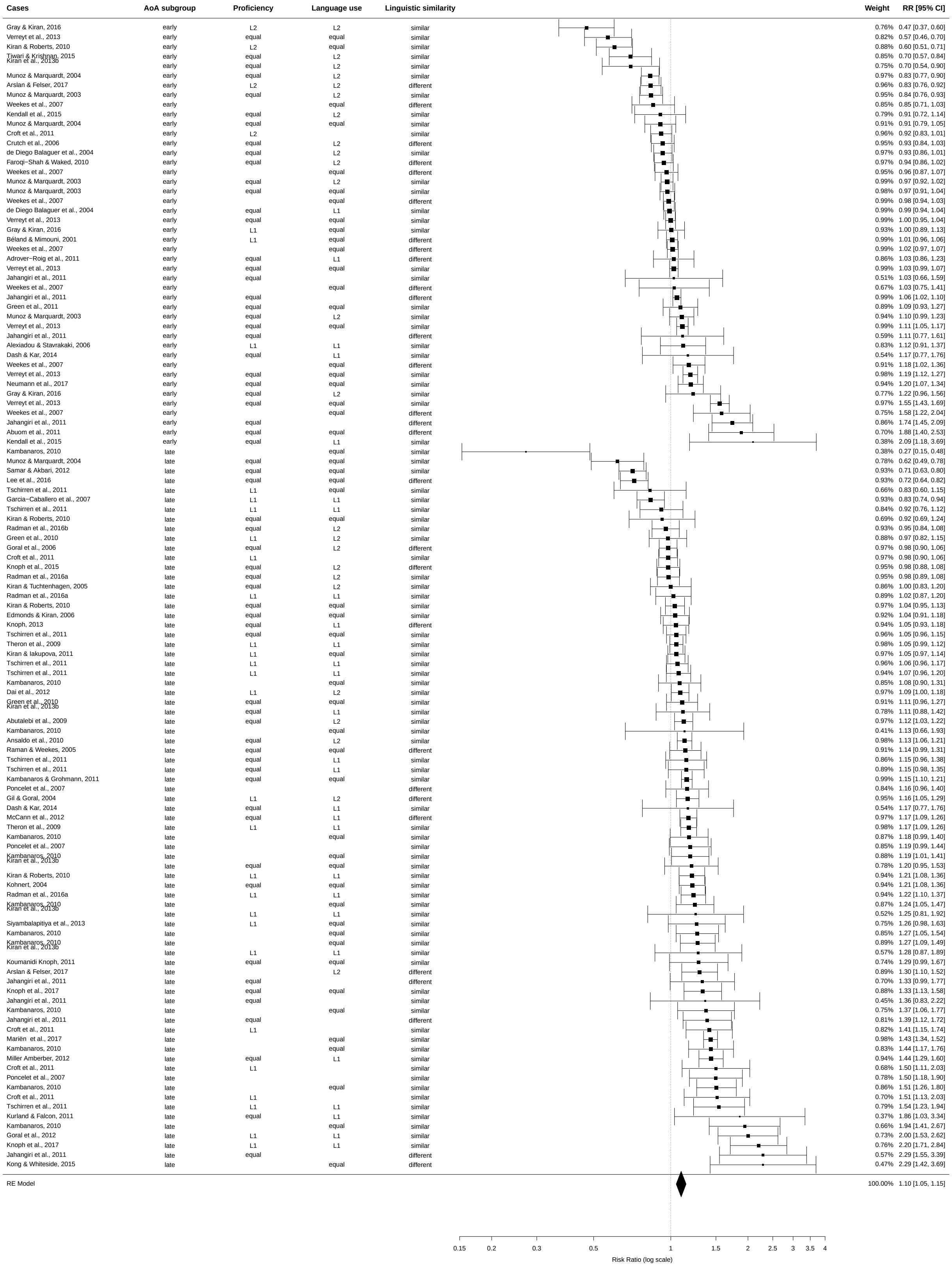
Cases AoA subgroup Proficiency Language use Linguistic similarity Weight RR [95% CI]
Gray & Kiran, 2016 early L2 L2 similar 0.76% 0.47 [0.37, 0.60]
Verreyt et al., 2013 early equal equal similar 0.82% 0.57 [0.46, 0.70]
Kiran & Roberts, 2010 early L2 equal similar 0.88% 0.60 [0.51, 0.71]
Tiwari & Krishnan, 2015 early equal L2 similar 0.85% 0.70 [0.57, 0.84]
Kiran et al., 2013b early equal L2 similar 0.75% 0.70 [0.54, 0.90]
Munoz & Marquardt, 2004 early equal L2 similar 0.97% 0.83 [0.77, 0.90]
Arslan & Felser, 2017 early L2 L2 different 0.96% 0.83 [0.76, 0.92]
Munoz & Marquardt, 2003 early equal L2 similar 0.95% 0.84 [0.76, 0.93]
Weekes et al., 2007 early equal different 0.85% 0.85 [0.71, 1.03]
Kendall et al., 2015 early equal L2 similar 0.79% 0.91 [0.72, 1.14]
Munoz & Marquardt, 2004 early equal equal similar 0.91% 0.91 [0.79, 1.05]
Croft et al., 2011 early L2 similar 0.96% 0.92 [0.83, 1.01]
Crutch et al., 2006 early equal L2 different 0.95% 0.93 [0.84, 1.03]
de Diego Balaguer et al., 2004 early equal L2 similar 0.97% 0.93 [0.86, 1.01]
Faroqi−Shah & Waked, 2010 early equal L2 different 0.97% 0.94 [0.86, 1.02]
Weekes et al., 2007 early equal different 0.95% 0.96 [0.87, 1.07]
Munoz & Marquardt, 2003 early equal L2 similar 0.99% 0.97 [0.92, 1.02]
Munoz & Marquardt, 2003 early equal equal similar 0.98% 0.97 [0.91, 1.04]
Weekes et al., 2007 early equal different 0.99% 0.98 [0.94, 1.03]
de Diego Balaguer et al., 2004 early equal L1 similar 0.99% 0.99 [0.94, 1.04]
Verreyt et al., 2013 early equal equal similar 0.99% 1.00 [0.95, 1.04]
Gray & Kiran, 2016 early L1 equal similar 0.93% 1.00 [0.89, 1.13]
Béland & Mimouni, 2001 early L1 equal different 0.99% 1.01 [0.96, 1.06]
Weekes et al., 2007 early equal different 0.99% 1.02 [0.97, 1.07]
Adrover−Roig et al., 2011 early equal L1 different 0.86% 1.03 [0.86, 1.23]
Verreyt et al., 2013 early equal equal similar 0.99% 1.03 [0.99, 1.07]
Jahangiri et al., 2011 early equal similar 0.51% 1.03 [0.66, 1.59]
Weekes et al., 2007 early equal different 0.67% 1.03 [0.75, 1.41]
Jahangiri et al., 2011 early equal different 0.99% 1.06 [1.02, 1.10]
Green et al., 2011 early equal equal similar 0.89% 1.09 [0.93, 1.27]
Munoz & Marquardt, 2003 early equal L2 similar 0.94% 1.10 [0.99, 1.23]
Verreyt et al., 2013 early equal equal similar 0.99% 1.11 [1.05, 1.17]
Jahangiri et al., 2011 early equal different 0.59% 1.11 [0.77, 1.61]
Alexiadou & Stavrakaki, 2006 early L1 L1 similar 0.83% 1.12 [0.91, 1.37]
Dash & Kar, 2014 early equal L1 similar 0.54% 1.17 [0.77, 1.76]
Weekes et al., 2007 early equal different 0.91% 1.18 [1.02, 1.36]
Verreyt et al., 2013 early equal equal similar 0.98% 1.19 [1.12, 1.27]
Neumann et al., 2017 early equal equal similar 0.94% 1.20 [1.07, 1.34]
Gray & Kiran, 2016 early equal L2 similar 0.77% 1.22 [0.96, 1.56]
Verreyt et al., 2013 early equal equal similar 0.97% 1.55 [1.43, 1.69]
Weekes et al., 2007 early equal different 0.75% 1.58 [1.22, 2.04]
Jahangiri et al., 2011 early equal different 0.86% 1.74 [1.45, 2.09]
Abuom et al., 2011 early equal equal different 0.70% 1.88 [1.40, 2.53]
Kendall et al., 2015 early equal L1 similar 0.38% 2.09 [1.18, 3.69]
Kambanaros, 2010 late equal similar 0.38% 0.27 [0.15, 0.48]
Munoz & Marquardt, 2004 late equal equal similar 0.78% 0.62 [0.49, 0.78]
Samar & Akbari, 2012 late equal equal similar 0.93% 0.71 [0.63, 0.80]
Lee et al., 2016 late equal equal different 0.93% 0.72 [0.64, 0.82]
Tschirren et al., 2011 late L1 equal similar 0.66% 0.83 [0.60, 1.15]
Garcia−Caballero et al., 2007 late L1 L1 similar 0.93% 0.83 [0.74, 0.94]
Tschirren et al., 2011 late L1 L1 similar 0.84% 0.92 [0.76, 1.12]
Kiran & Roberts, 2010 late equal equal similar 0.69% 0.92 [0.69, 1.24]
Radman et al., 2016b late equal L2 similar 0.93% 0.95 [0.84, 1.08]
Green et al., 2010 late L1 L2 similar 0.88% 0.97 [0.82, 1.15]
Goral et al., 2006 late equal L2 different 0.97% 0.98 [0.90, 1.06]
Croft et al., 2011 late L1 similar 0.97% 0.98 [0.90, 1.06]
Knoph et al., 2015 late equal L2 different 0.95% 0.98 [0.88, 1.08]
Radman et al., 2016a late equal L2 similar 0.95% 0.98 [0.89, 1.08]
Kiran & Tuchtenhagen, 2005 late equal L2 similar 0.86% 1.00 [0.83, 1.20]
Radman et al., 2016a late L1 L1 similar 0.89% 1.02 [0.87, 1.20]
Kiran & Roberts, 2010 late equal equal similar 0.97% 1.04 [0.95, 1.13]
Edmonds & Kiran, 2006 late equal equal similar 0.92% 1.04 [0.91, 1.18]
Knoph, 2013 late equal L1 different 0.94% 1.05 [0.93, 1.18]
Tschirren et al., 2011 late equal equal similar 0.96% 1.05 [0.96, 1.15]
Theron et al., 2009 late L1 L1 similar 0.98% 1.05 [0.99, 1.12]
Kiran & Iakupova, 2011 late L1 equal similar 0.97% 1.05 [0.97, 1.14]
Tschirren et al., 2011 late L1 L1 similar 0.96% 1.06 [0.96, 1.17]
Tschirren et al., 2011 late L1 L1 similar 0.94% 1.07 [0.96, 1.20]
Kambanaros, 2010 late equal similar 0.85% 1.08 [0.90, 1.31]
Dai et al., 2012 late L1 L2 similar 0.97% 1.09 [1.00, 1.18]
Green et al., 2010 late equal equal similar 0.91% 1.11 [0.96, 1.27]
Kiran et al., 2013b late equal L1 similar 0.78% 1.11 [0.88, 1.42]
Abutalebi et al., 2009 late equal L2 similar 0.97% 1.12 [1.03, 1.22]
Kambanaros, 2010 late equal similar 0.41% 1.13 [0.66, 1.93]
Ansaldo et al., 2010 late equal L2 similar 0.98% 1.13 [1.06, 1.21]
Raman & Weekes, 2005 late equal equal different 0.91% 1.14 [0.99, 1.31]
Tschirren et al., 2011 late equal L1 similar 0.86% 1.15 [0.96, 1.38]
Tschirren et al., 2011 late equal L1 similar 0.89% 1.15 [0.98, 1.35]
Kambanaros & Grohmann, 2011 late equal equal similar 0.99% 1.15 [1.10, 1.21]
Poncelet et al., 2007 late different 0.84% 1.16 [0.96, 1.40]
Gil & Goral, 2004 late L1 L2 different 0.95% 1.16 [1.05, 1.29]
Dash & Kar, 2014 late equal L1 similar 0.54% 1.17 [0.77, 1.76]
McCann et al., 2012 late equal L1 different 0.97% 1.17 [1.09, 1.26]
Theron et al., 2009 late L1 L1 similar 0.98% 1.17 [1.09, 1.26]
Kambanaros, 2010 late equal similar 0.87% 1.18 [0.99, 1.40]
Poncelet et al., 2007 late similar 0.85% 1.19 [0.99, 1.44]
Kambanaros, 2010 late equal similar 0.88% 1.19 [1.01, 1.41]
Kiran et al., 2013b late equal equal similar 0.78% 1.20 [0.95, 1.53]
Kiran & Roberts, 2010 late L1 L1 similar 0.94% 1.21 [1.08, 1.36]
Kohnert, 2004 late equal equal similar 0.94% 1.21 [1.08, 1.36]
Radman et al., 2016a late L1 L1 similar 0.94% 1.22 [1.10, 1.37]
Kambanaros, 2010 late equal similar 0.87% 1.24 [1.05, 1.47]
Kiran et al., 2013b late L1 L1 similar 0.52% 1.25 [0.81, 1.92]
Siyambalapitiya et al., 2013 late L1 equal similar 0.75% 1.26 [0.98, 1.63]
Kambanaros, 2010 late equal similar 0.85% 1.27 [1.05, 1.54]
Kambanaros, 2010 late equal similar 0.89% 1.27 [1.09, 1.49]
Kiran et al., 2013b late L1 L1 similar 0.57% 1.28 [0.87, 1.89]
Koumanidi Knoph, 2011 late equal equal similar 0.74% 1.29 [0.99, 1.67]
Arslan & Felser, 2017 late L2 different 0.89% 1.30 [1.10, 1.52]
Jahangiri et al., 2011 late equal different 0.70% 1.33 [0.99, 1.77]
Knoph et al., 2017 late equal equal similar 0.88% 1.33 [1.13, 1.58]
Jahangiri et al., 2011 late equal similar 0.45% 1.36 [0.83, 2.22]
Kambanaros, 2010 late equal similar 0.75% 1.37 [1.06, 1.77]
Jahangiri et al., 2011 late equal different 0.81% 1.39 [1.12, 1.72]
Croft et al., 2011 late L1 similar 0.82% 1.41 [1.15, 1.74]
Mariën et al., 2017 late equal similar 0.98% 1.43 [1.34, 1.52]
Kambanaros, 2010 late equal similar 0.83% 1.44 [1.17, 1.76]
Miller Amberber, 2012 late equal L1 similar 0.94% 1.44 [1.29, 1.60]
Croft et al., 2011 late L1 similar 0.68% 1.50 [1.11, 2.03]
Poncelet et al., 2007 late similar 0.78% 1.50 [1.18, 1.90]
Kambanaros, 2010 late equal similar 0.86% 1.51 [1.26, 1.80]
Croft et al., 2011 late L1 similar 0.70% 1.51 [1.13, 2.03]
Tschirren et al., 2011 late L1 L1 similar 0.79% 1.54 [1.23, 1.94]
Kurland & Falcon, 2011 late equal L1 similar 0.37% 1.86 [1.03, 3.34]
Kambanaros, 2010 late equal similar 0.66% 1.94 [1.41, 2.67]
Goral et al., 2012 late L1 L1 similar 0.73% 2.00 [1.53, 2.62]
Knoph et al., 2017 late L1 L1 similar 0.76% 2.20 [1.71, 2.84]
Jahangiri et al., 2011 late equal different 0.57% 2.29 [1.55, 3.39]
Kong & Whiteside, 2015 late equal different 0.47% 2.29 [1.42, 3.69]
RE Model 100.00% 1.10 [1.05, 1.15]
0.15 0.2 0.3 0.5 1 1.5 2 2.5 3 3.5 4
Risk Ratio (log scale)

Auditorry Comprehension

```r
print(m2<-rma(yi,vi,data=data_ac))
```

```
##
## Random-Effects Model (k = 91; tau^2 estimator: REML)
##
## tau^2 (estimated amount of total heterogeneity): 0.0225 (SE = 0.0047)
## tau (square root of estimated tau^2 value):      0.1500
## I^2 (total heterogeneity / total variability):   86.98%
## H^2 (total variability / sampling variability):  7.68
##
## Test for Heterogeneity:
## Q(df = 90) = 363.4057, p-val < .0001
##
## Model Results:
##
## estimate      se    zval    pval   ci.lb   ci.ub
##   0.0609  0.0190  3.2066  0.0013  0.0237  0.0982  **
##
## ---
## Signif. codes:  0 '***' 0.001 '**' 0.01 '*' 0.05 '.' 0.1 ' ' 1
```

```r
#RR estimate,lower and upper boundaries of the 95% CI
round(exp(c(m2$b,m2$ci.lb,m2$ci.ub)),digits=2)
```

```
## [1] 1.06 1.02 1.10
```

Oral Production

```r
print(m3<-rma(yi,vi,data=data_op))
```

```
##
## Random-Effects Model (k = 91; tau^2 estimator: REML)
##
## tau^2 (estimated amount of total heterogeneity): 0.0768 (SE = 0.0135)
## tau (square root of estimated tau^2 value):      0.2770
## I^2 (total heterogeneity / total variability):   93.89%
## H^2 (total variability / sampling variability):  16.37
##
## Test for Heterogeneity:
## Q(df = 90) = 686.2548, p-val < .0001
##
## Model Results:
##
## estimate      se    zval    pval   ci.lb   ci.ub
##   0.0921  0.0317  2.9003  0.0037  0.0299  0.1543  **
##
## ---
## Signif. codes:  0 '***' 0.001 '**' 0.01 '*' 0.05 '.' 0.1 ' ' 1
```

```r
#RR estimate,lower and upper boundaries of the 95% CI
round(exp(c(m3$b,m3$ci.lb,m3$ci.ub)),digits=2)
```

```
## [1] 1.10 1.03 1.17
```

Moderator Analysis - Part1

Age of Language Aquisition (AoA)

Does continuous AoA moderate the difference betwen L1 and L2?

Overall Performance

```
summary(data$aoa_adj)
```

```
##    Min. 1st Qu.  Median    Mean 3rd Qu.    Max.    NA's
##    2.50    5.00   10.00   12.15   19.25   40.00       3
```

```
print(m4<-rma(yi,vi,mods=aoa_adj,data=data))
```

```
##
## Mixed-Effects Model (k = 116; tau^2 estimator: REML)
##
## tau^2 (estimated amount of residual heterogeneity):     0.0474 (SE = 0.0074)
## tau (square root of estimated tau^2 value):             0.2177
## I^2 (residual heterogeneity / unaccounted variability): 94.51%
## H^2 (unaccounted variability / sampling variability):   18.21
## R^2 (amount of heterogeneity accounted for):            9.88%
##
## Test for Residual Heterogeneity:
## QE(df = 114) = 897.5573, p-val < .0001
##
## Test of Moderators (coefficient(s) 2):
## QM(df = 1) = 8.8427, p-val = 0.0029
##
## Model Results:
##
##          estimate      se     zval    pval    ci.lb   ci.ub
## intrcpt   -0.0037  0.0381  -0.0967  0.9229  -0.0784  0.0710
## mods       0.0077  0.0026   2.9737  0.0029   0.0026  0.0127  **
##
## ---
## Signif. codes:  0 '***' 0.001 '**' 0.01 '*' 0.05 '.' 0.1 ' ' 1
```

```
#RR estimate,lower and upper boundaries of the 95% CI
round(exp(c(m4$b,m4$ci.lb,m4$ci.ub)),digits=2)
```

```
## [1] 1.00 1.01 0.92 1.00 1.07 1.01
```

Auditory Comprehension

```r
summary(data_ac$aoa_adj)
```

```
##    Min. 1st Qu.  Median    Mean 3rd Qu.    Max.
##    2.50    5.00    9.00   11.18   16.00   40.00
```

```r
print(m5<-rma(yi,vi,mods=aoa_adj,data=data_ac))
```

```
##
## Mixed-Effects Model (k = 91; tau^2 estimator: REML)
##
## tau^2 (estimated amount of residual heterogeneity):     0.0221 (SE = 0.0046)
## tau (square root of estimated tau^2 value):             0.1487
## I^2 (residual heterogeneity / unaccounted variability): 86.50%
## H^2 (unaccounted variability / sampling variability):   7.41
## R^2 (amount of heterogeneity accounted for):            1.80%
##
## Test for Residual Heterogeneity:
## QE(df = 89) = 353.0053, p-val < .0001
##
## Test of Moderators (coefficient(s) 2):
## QM(df = 1) = 1.6511, p-val = 0.1988
##
## Model Results:
##
##          estimate      se    zval    pval    ci.lb   ci.ub
## intrcpt    0.0298  0.0306  0.9759  0.3291  -0.0301  0.0898
## mods       0.0028  0.0022  1.2849  0.1988  -0.0015  0.0071
##
## ---
## Signif. codes:  0 '***' 0.001 '**' 0.01 '*' 0.05 '.' 0.1 ' ' 1
```

```r
#RR estimate,lower and upper boundaries of the 95% CI
round(exp(c(m5$b,m5$ci.lb,m5$ci.ub)),digits=2)
```

```
## [1] 1.03 1.00 0.97 1.00 1.09 1.01
```

Oral Production

```r
summary(data_op$aoa_adj)
```

```
##    Min. 1st Qu.  Median    Mean 3rd Qu.    Max.    NA's 
##    2.50    5.00   10.00   12.82   20.00   40.00       3
```

```r
print(m6<-rma(yi,vi,mods=aoa_adj,data=data_op))
```

```
## 
## Mixed-Effects Model (k = 88; tau^2 estimator: REML)
## 
## tau^2 (estimated amount of residual heterogeneity):     0.0638 (SE = 0.0118)
## tau (square root of estimated tau^2 value):             0.2526
## I^2 (residual heterogeneity / unaccounted variability): 92.78%
## H^2 (unaccounted variability / sampling variability):   13.86
## R^2 (amount of heterogeneity accounted for):            19.29%
## 
## Test for Residual Heterogeneity:
## QE(df = 86) = 565.4312, p-val < .0001
## 
## Test of Moderators (coefficient(s) 2):
## QM(df = 1) = 13.6097, p-val = 0.0002
## 
## Model Results:
## 
##          estimate      se     zval    pval    ci.lb   ci.ub 
## intrcpt   -0.0708  0.0521  -1.3572  0.1747  -0.1730  0.0314 
## mods       0.0126  0.0034   3.6891  0.0002   0.0059  0.0193  *** 
## 
## ---
## Signif. codes:  0 '***' 0.001 '**' 0.01 '*' 0.05 '.' 0.1 ' ' 1
```

```r
#RR estimate,lower and upper boundaries of the 95% CI
round(exp(c(m6$b,m6$ci.lb,m6$ci.ub)),digits=2)
```

```
## [1] 0.93 1.01 0.84 1.01 1.03 1.02
```

Does the early-late bilingual status based on 7 years cut-off moderate the difference betwen L1 and L2?

Plot based on 7 years as a cut-off for Overall Performance

```
p <- ggplot(data, aes(x=aoa_adj,
                      y=yi ,
                      color=early_or_late))+
  scale_color_manual(name = "AoA subgroup",
                     values = c("deeppink1", "darkviolet")) +
  geom_point(size = 1.5)+
  geom_hline(yintercept=0, linetype="dashed", color = "black")+
  ylim(-2.5, 2.5)+
  xlim(2, 40)+
  labs(y="Risk Ratio (log)", x = "AoA, year")
p
```

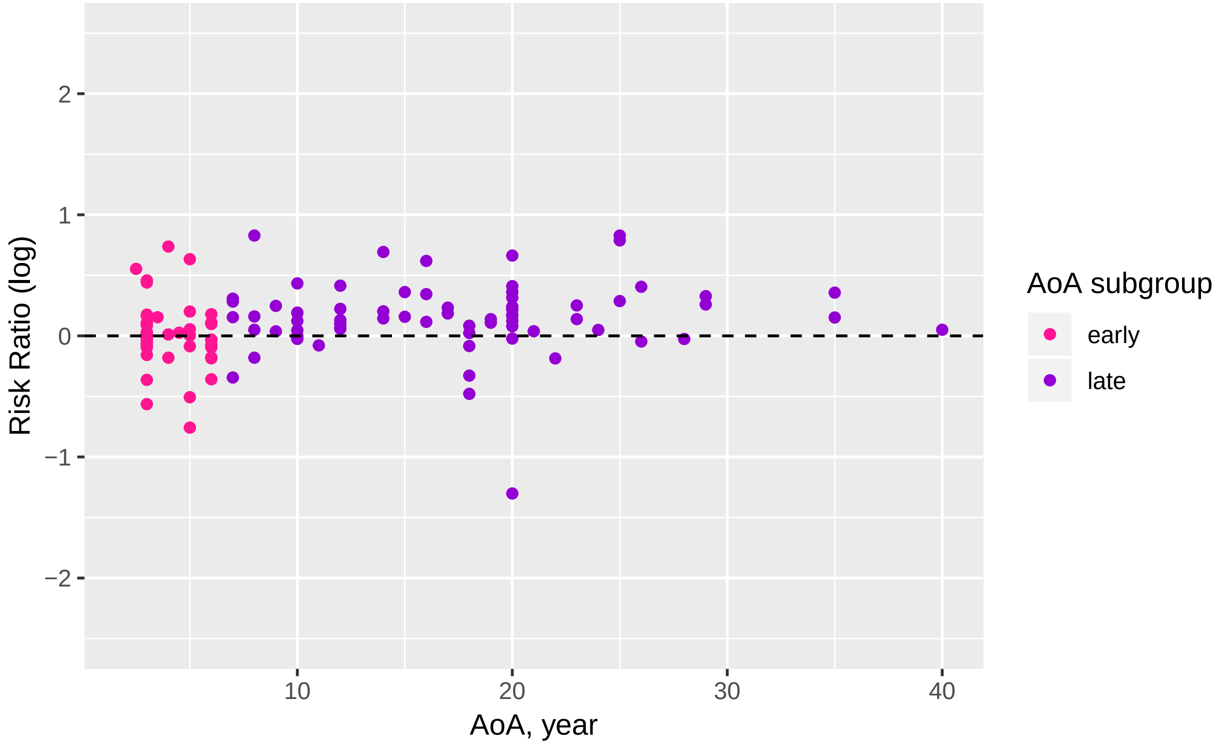

Overall Performance

```
data$late <-ifelse(data$early_or_late=="late",1,0)
data$early<-ifelse(data$early_or_late=="early",1,0)
```

```
print(c(sum(data$late=="1"),sum(data$early=="1")))
```

```
## [1] 75 44
```

```
print(m7<-rma(yi,vi,mods=cbind(late,early),intercept=F,data=data))
```

```
##
## Mixed-Effects Model (k = 119; tau^2 estimator: REML)
##
## tau^2 (estimated amount of residual heterogeneity):     0.0458 (SE = 0.0071)
## tau (square root of estimated tau^2 value):             0.2141
## I^2 (residual heterogeneity / unaccounted variability): 94.21%
## H^2 (unaccounted variability / sampling variability):   17.29
##
## Test for Residual Heterogeneity:
## QE(df = 117) = 918.7905, p-val < .0001
##
## Test of Moderators (coefficient(s) 1:2):
## QM(df = 2) = 29.4702, p-val < .0001
##
## Model Results:
##
##        estimate      se     zval    pval    ci.lb   ci.ub
## late     0.1493  0.0275   5.4286  <.0001   0.0954  0.2032  ***
## early   -0.0005  0.0349  -0.0151  0.9880  -0.0689  0.0679
##
## ---
## Signif. codes:  0 '***' 0.001 '**' 0.01 '*' 0.05 '.' 0.1 ' ' 1
```

```
#RR estimate,lower and upper boundaries of the 95% CI
round(exp(c(m7$b,m7$ci.lb,m7$ci.ub)),digits=2)
```

```
## [1] 1.16 1.00 1.10 0.93 1.23 1.07
```

Checking whether the eraly-late bilingual status is a significant moderator.

```
capture.output(rma(yi,vi,mods=cbind(late,early),data=data))[c(8,10,11,13,14)]
```

```
## [1] "R^2 (amount of heterogeneity accounted for):            11.38%"
## [2] "Test for Residual Heterogeneity: "
## [3] "QE(df = 117) = 918.7905, p-val < .0001"
## [4] "Test of Moderators (coefficient(s) 2): "
## [5] "QM(df = 1) = 11.3704, p-val = 0.0007"
```

Auditory Comprehension

```
data_ac$late <-ifelse(data_ac$early_or_late=="late",1,0)
data_ac$early<-ifelse(data_ac$early_or_late=="early",1,0)
```

```
summary(as.factor(data_ac$late))
```

```
##  0  1
## 38 53
```

```
print(m8<-rma(yi,vi,mods=cbind(late,early),intercept=F,data=data_ac))
```

```
##
## Mixed-Effects Model (k = 91; tau^2 estimator: REML)
##
## tau^2 (estimated amount of residual heterogeneity):     0.0217 (SE = 0.0046)
## tau (square root of estimated tau^2 value):             0.1473
## I^2 (residual heterogeneity / unaccounted variability): 86.12%
## H^2 (unaccounted variability / sampling variability):   7.20
##
## Test for Residual Heterogeneity:
## QE(df = 89) = 341.2154, p-val < .0001
##
## Test of Moderators (coefficient(s) 1:2):
## QM(df = 2) = 13.0365, p-val = 0.0015
##
## Model Results:
##
##        estimate      se    zval    pval    ci.lb   ci.ub
## late     0.0875  0.0251  3.4832  0.0005   0.0382  0.1367  ***
## early    0.0268  0.0281  0.9508  0.3417  -0.0284  0.0819
##
## ---
## Signif. codes:  0 '***' 0.001 '**' 0.01 '*' 0.05 '.' 0.1 ' ' 1
```

```
#RR estimate,lower and upper boundaries of the 95% CI
round(exp(c(m8$b,m8$ci.lb,m8$ci.ub)),digits=2)
```

```
## [1] 1.09 1.03 1.04 0.97 1.15 1.09
```

Checking whether the eraly-late bilingual status is a significant moderator.

```
capture.output(rma(yi,vi,mods=cbind(late,early),data=data_ac))[c(8,10,11,13,14)]
```

```
## [1] "R^2 (amount of heterogeneity accounted for):            3.69%"
## [2] "Test for Residual Heterogeneity: "
## [3] "QE(df = 89) = 341.2154, p-val < .0001"
## [4] "Test of Moderators (coefficient(s) 2): "
## [5] "QM(df = 1) = 2.5893, p-val = 0.1076"
```

Oral Production

```
data_op$late<-ifelse(data_op$early_or_late=="late",1,0)
data_op$early<-ifelse(data_op$early_or_late=="early",1,0)
```

```
summary(as.factor(data_op$late))
```

```
##  0  1
## 32 59
```

```
print(m9<-rma(yi,vi,mods=cbind(late,early),intercept=F,data=data_op))
```

```
##
## Mixed-Effects Model (k = 91; tau^2 estimator: REML)
##
## tau^2 (estimated amount of residual heterogeneity):     0.0672 (SE = 0.0121)
## tau (square root of estimated tau^2 value):             0.2593
## I^2 (residual heterogeneity / unaccounted variability): 92.97%
## H^2 (unaccounted variability / sampling variability):   14.22
##
## Test for Residual Heterogeneity:
## QE(df = 89) = 611.8257, p-val < .0001
##
## Test of Moderators (coefficient(s) 1:2):
## QM(df = 2) = 18.2783, p-val = 0.0001
##
## Model Results:
##
##        estimate      se     zval    pval    ci.lb   ci.ub
## late     0.1597  0.0376   4.2439  <.0001   0.0860  0.2335  ***
## early   -0.0257  0.0497  -0.5177  0.6047  -0.1231  0.0717
##
## ---
## Signif. codes:  0 '***' 0.001 '**' 0.01 '*' 0.05 '.' 0.1 ' ' 1
```

```
#RR estimate,lower and upper boundaries of the 95% CI
round(exp(c(m9$b,m9$ci.lb,m9$ci.ub)),digits=2)
```

```
## [1] 1.17 0.97 1.09 0.88 1.26 1.07
```

Checking whether the eraly-late bilingual status is a significant moderator.

```
capture.output(rma(yi,vi,mods=cbind(late,early),data=data_op))[c(8,10,11,13,14)]
```

```
## [1] "R^2 (amount of heterogeneity accounted for):            12.40%"
## [2] "Test for Residual Heterogeneity: "
## [3] "QE(df = 89) = 611.8257, p-val < .0001"
## [4] "Test of Moderators (coefficient(s) 2): "
## [5] "QM(df = 1) = 8.8499, p-val = 0.0029"
```

Creating subsets for the early and late subgroups

```r
#for overall performance
data_e_overall<-data[data$early=="1",]
data_l_overall<-data[data$late=="1",]

#for auditory comprehension
data_e_ac<-data_ac[data_ac$early=="1",]
data_l_ac<-data_ac[data_ac$late=="1",]

#for oral production
data_e_op<-data_op[data_op$early=="1",]
data_l_op<-data_op[data_op$late=="1",]
```

Demographic and clinical details

```
cols<-c("case_age","case_scholarity_years","case_gender","case_mpo","aoa_adj","case_lesion_side",
        "proficiency","language_use","ling_similar_2levels","ling_similar_3levels")
```

Whole group

```
sapply(data[cols],summary)
```

```
## $case_age
##    Min. 1st Qu.  Median    Mean 3rd Qu.    Max. 
##    17.0    47.7    59.0    58.5    69.0    91.0 
## 
## $case_scholarity_years
##    Min. 1st Qu.  Median    Mean 3rd Qu.    Max.    NA's 
##    1.00    9.50   12.00   12.24   16.00   22.00      48 
## 
## $case_gender
##  f  m 
## 57 62 
## 
## $case_mpo
##    Min. 1st Qu.  Median    Mean 3rd Qu.    Max.    NA's 
##    1.00   15.00   29.00   28.33   43.00   53.00      13 
## 
## $aoa_adj
##    Min. 1st Qu.  Median    Mean 3rd Qu.    Max.    NA's 
##    2.50    5.00   10.00   12.15   19.25   40.00       3 
## 
## $case_lesion_side
## both    l    r NA's 
##    1  100    5   13 
## 
## $proficiency
## equal    L1    L2  NA's 
##    63    27     4    25 
## 
## $language_use
## equal    L1    L2  NA's 
##    52    27    24    16 
## 
## $ling_similar_2levels
## different   similar 
##        29        90 
## 
## $ling_similar_3levels
##      close  different very close 
##         69         29         21
```

Early subgroup

```r
sapply(data_e_overall[cols],summary)
```

```
## $case_age
##    Min. 1st Qu.  Median    Mean 3rd Qu.    Max. 
##   17.00   43.00   53.50   52.87   62.25   84.00 
## 
## $case_scholarity_years
##    Min. 1st Qu.  Median    Mean 3rd Qu.    Max.    NA's 
##    8.00   11.25   12.00   13.19   16.00   22.00      18 
## 
## $case_gender
##  f  m 
## 21 23 
## 
## $case_mpo
##    Min. 1st Qu.  Median    Mean 3rd Qu.    Max.    NA's 
##    2.00   14.75   29.50   28.34   39.50   53.00      12 
## 
## $aoa_adj
##    Min. 1st Qu.  Median    Mean 3rd Qu.    Max. 
##   2.500   3.000   3.250   4.102   5.000   6.000 
## 
## $case_lesion_side
## both    l    r NA's 
##    1   39    3    1 
## 
## $proficiency
## equal    L1    L2  NA's 
##    30     3     4     7 
## 
## $language_use
## equal    L1    L2  NA's 
##    21     5    13     5 
## 
## $ling_similar_2levels
## different   similar 
##        16        28 
## 
## $ling_similar_3levels
##      close  different very close 
##         21         16          7
```

Late subgroup

```
sapply(data_l_overall[cols],summary)
```

```
## $case_age
##    Min. 1st Qu.  Median    Mean 3rd Qu.    Max. 
##    33.0    52.0    63.0    61.8    72.0    91.0 
## 
## $case_scholarity_years
##    Min. 1st Qu.  Median    Mean 3rd Qu.    Max.    NA's 
##    1.00    6.00   12.00   11.69   16.00   22.00      30 
## 
## $case_gender
##  f  m 
## 36 39 
## 
## $case_mpo
##    Min. 1st Qu.  Median    Mean 3rd Qu.    Max.    NA's 
##    1.00   15.00   29.00   28.32   43.00   52.00       1 
## 
## $aoa_adj
##    Min. 1st Qu.  Median    Mean 3rd Qu.    Max.    NA's 
##    7.00   10.00   17.50   17.07   20.00   40.00       3 
## 
## $case_lesion_side
## both    l    r NA's 
##    0   61    2   12 
## 
## $proficiency
## equal    L1    L2  NA's 
##    33    24     0    18 
## 
## $language_use
## equal    L1    L2  NA's 
##    31    22    11    11 
## 
## $ling_similar_2levels
## different   similar 
##        13        62 
## 
## $ling_similar_3levels
##      close  different very close 
##         48         13         14
```

Does continuous AoA moderate the difference between L1 and L2 in the early and late subgroups separately?

Early subgroup - Overall Performance

```
summary(data_e_overall$aoa_adj)
```

```
##    Min. 1st Qu.  Median    Mean 3rd Qu.    Max.
##   2.500   3.000   3.250   4.102   5.000   6.000
```

```
print(m10<-rma(yi,vi,mods=aoa_adj,data=data_e_overall))
```

```
##
## Mixed-Effects Model (k = 44; tau^2 estimator: REML)
##
## tau^2 (estimated amount of residual heterogeneity):     0.0536 (SE = 0.0133)
## tau (square root of estimated tau^2 value):             0.2314
## I^2 (residual heterogeneity / unaccounted variability): 96.62%
## H^2 (unaccounted variability / sampling variability):   29.63
## R^2 (amount of heterogeneity accounted for):            2.02%
##
## Test for Residual Heterogeneity:
## QE(df = 42) = 412.4821, p-val < .0001
##
## Test of Moderators (coefficient(s) 2):
## QM(df = 1) = 1.7191, p-val = 0.1898
##
## Model Results:
##
##          estimate      se     zval    pval    ci.lb   ci.ub
## intrcpt    0.1580  0.1260   1.2537  0.2099  -0.0890  0.4049
## mods      -0.0382  0.0292  -1.3112  0.1898  -0.0954  0.0189
##
## ---
## Signif. codes:  0 '***' 0.001 '**' 0.01 '*' 0.05 '.' 0.1 ' ' 1
```

```
#RR estimate,lower and upper boundaries of the 95% CI
round(exp(c(m10$b,m10$ci.lb,m10$ci.ub)),digits=2)
```

```
## [1] 1.17 0.96 0.91 0.91 1.50 1.02
```

Early subgroup - Auditory Comprehension

```r
summary(data_e_ac$aoa_adj)
```

```
##    Min. 1st Qu.  Median    Mean 3rd Qu.    Max.
##   2.500   3.000   3.500   4.184   5.750   6.000
```

```r
print(m11<-rma(yi,vi,mods=aoa_adj,data=data_e_ac))
```

```
##
## Mixed-Effects Model (k = 38; tau^2 estimator: REML)
##
## tau^2 (estimated amount of residual heterogeneity):     0.0207 (SE = 0.0066)
## tau (square root of estimated tau^2 value):             0.1440
## I^2 (residual heterogeneity / unaccounted variability): 86.53%
## H^2 (unaccounted variability / sampling variability):   7.43
## R^2 (amount of heterogeneity accounted for):            2.38%
##
## Test for Residual Heterogeneity:
## QE(df = 36) = 136.5423, p-val < .0001
##
## Test of Moderators (coefficient(s) 2):
## QM(df = 1) = 2.8282, p-val = 0.0926
##
## Model Results:
##
##          estimate      se     zval    pval    ci.lb   ci.ub
## intrcpt    0.1784  0.0946   1.8868  0.0592  -0.0069  0.3638  .
## mods      -0.0353  0.0210  -1.6817  0.0926  -0.0765  0.0058  .
##
## ---
## Signif. codes:  0 '***' 0.001 '**' 0.01 '*' 0.05 '.' 0.1 ' ' 1
```

```r
#RR estimate,lower and upper boundaries of the 95% CI
round(exp(c(m11$b,m11$ci.lb,m11$ci.ub)),digits=2)
```

```
## [1] 1.20 0.97 0.99 0.93 1.44 1.01
```

Early subgroup - Oral Production

```r
summary(data_e_op$aoa_adj)
```

```
##    Min. 1st Qu.  Median    Mean 3rd Qu.    Max.
##   2.500   3.000   4.000   4.203   5.250   6.000
```

```r
print(m12<-rma(yi,vi,mods=aoa_adj,data=data_e_op))
```

```
##
## Mixed-Effects Model (k = 32; tau^2 estimator: REML)
##
## tau^2 (estimated amount of residual heterogeneity):     0.0835 (SE = 0.0245)
## tau (square root of estimated tau^2 value):             0.2890
## I^2 (residual heterogeneity / unaccounted variability): 96.53%
## H^2 (unaccounted variability / sampling variability):   28.78
## R^2 (amount of heterogeneity accounted for):            0.00%
##
## Test for Residual Heterogeneity:
## QE(df = 30) = 262.1162, p-val < .0001
##
## Test of Moderators (coefficient(s) 2):
## QM(df = 1) = 0.0615, p-val = 0.8041
##
## Model Results:
##
##          estimate      se     zval    pval    ci.lb   ci.ub
## intrcpt    0.0195  0.1884   0.1034  0.9176  -0.3498  0.3888
## mods      -0.0106  0.0426  -0.2481  0.8041  -0.0941  0.0729
##
## ---
## Signif. codes:  0 '***' 0.001 '**' 0.01 '*' 0.05 '.' 0.1 ' ' 1
```

```r
#RR estimate,lower and upper boundaries of the 95% CI
round(exp(c(m12$b,m12$ci.lb,m12$ci.ub)),digits=2)
```

```
## [1] 1.02 0.99 0.70 0.91 1.48 1.08
```

Late subgroup - Overall Performance

```r
summary(data_l_overall$aoa_adj)
```

```
##    Min. 1st Qu.  Median    Mean 3rd Qu.    Max.    NA's
##    7.00   10.00   17.50   17.07   20.00   40.00       3
```

```r
print(m13<-rma(yi,vi,mods=aoa_adj,data=data_l_overall))
```

```
##
## Mixed-Effects Model (k = 72; tau^2 estimator: REML)
##
## tau^2 (estimated amount of residual heterogeneity):     0.0405 (SE = 0.0085)
## tau (square root of estimated tau^2 value):             0.2013
## I^2 (residual heterogeneity / unaccounted variability): 90.76%
## H^2 (unaccounted variability / sampling variability):   10.82
## R^2 (amount of heterogeneity accounted for):            2.04%
##
## Test for Residual Heterogeneity:
## QE(df = 70) = 441.6979, p-val < .0001
##
## Test of Moderators (coefficient(s) 2):
## QM(df = 1) = 1.4068, p-val = 0.2356
##
## Model Results:
##
##          estimate      se    zval    pval    ci.lb   ci.ub
## intrcpt    0.0713  0.0673  1.0593  0.2895  -0.0606  0.2031
## mods       0.0043  0.0036  1.1861  0.2356  -0.0028  0.0113
##
## ---
## Signif. codes:  0 '***' 0.001 '**' 0.01 '*' 0.05 '.' 0.1 ' ' 1
```

```r
#RR estimate,lower and upper boundaries of the 95% CI
round(exp(c(m13$b,m13$ci.lb,m13$ci.ub)),digits=2)
```

```
## [1] 1.07 1.00 0.94 1.00 1.23 1.01
```

Late subgroup - Auditory Comprehension

```
summary(data_l_ac$aoa_adj)
```

```
##    Min. 1st Qu.  Median    Mean 3rd Qu.    Max. 
##    7.00   10.00   15.00   16.19   19.00   40.00
```

```
print(m14<-rma(yi,vi,mods=aoa_adj,data=data_l_ac))
```

```
## 
## Mixed-Effects Model (k = 53; tau^2 estimator: REML)
## 
## tau^2 (estimated amount of residual heterogeneity):     0.0227 (SE = 0.0065)
## tau (square root of estimated tau^2 value):             0.1508
## I^2 (residual heterogeneity / unaccounted variability): 84.62%
## H^2 (unaccounted variability / sampling variability):   6.50
## R^2 (amount of heterogeneity accounted for):            0.00%
## 
## Test for Residual Heterogeneity:
## QE(df = 51) = 199.1030, p-val < .0001
## 
## Test of Moderators (coefficient(s) 2):
## QM(df = 1) = 0.2316, p-val = 0.6304
## 
## Model Results:
## 
##          estimate      se    zval    pval    ci.lb   ci.ub 
## intrcpt    0.0636  0.0566  1.1234  0.2613  -0.0474  0.1746    
## mods       0.0015  0.0031  0.4812  0.6304  -0.0046  0.0076    
## 
## ---
## Signif. codes:  0 '***' 0.001 '**' 0.01 '*' 0.05 '.' 0.1 ' ' 1
```

```
#RR estimate,lower and upper boundaries of the 95% CI
round(exp(c(m14$b,m14$ci.lb,m14$ci.ub)),digits=2)
```

```
## [1] 1.07 1.00 0.95 1.00 1.19 1.01
```

Late subgroup - Oral Production

```r
summary(data_l_op$aoa_adj)
```

```
##    Min. 1st Qu.  Median    Mean 3rd Qu.    Max.    NA's 
##    7.00   11.50   18.00   17.75   20.00   40.00       3
```

```r
print(m15<-rma(yi,vi,mods=aoa_adj,data=data_l_op))
```

```
## 
## Mixed-Effects Model (k = 56; tau^2 estimator: REML)
## 
## tau^2 (estimated amount of residual heterogeneity):     0.0547 (SE = 0.0134)
## tau (square root of estimated tau^2 value):             0.2338
## I^2 (residual heterogeneity / unaccounted variability): 86.33%
## H^2 (unaccounted variability / sampling variability):   7.32
## R^2 (amount of heterogeneity accounted for):            14.76%
## 
## Test for Residual Heterogeneity:
## QE(df = 54) = 265.7505, p-val < .0001
## 
## Test of Moderators (coefficient(s) 2):
## QM(df = 1) = 6.1520, p-val = 0.0131
## 
## Model Results:
## 
##          estimate      se     zval    pval    ci.lb   ci.ub   
## intrcpt   -0.0517  0.0910  -0.5683  0.5698  -0.2302  0.1267   
## mods       0.0119  0.0048   2.4803  0.0131   0.0025  0.0213  *
## 
## ---
## Signif. codes:  0 '***' 0.001 '**' 0.01 '*' 0.05 '.' 0.1 ' ' 1
```

```r
#RR estimate,lower and upper boundaries of the 95% CI
round(exp(c(m15$b,m15$ci.lb,m15$ci.ub)),digits=2)
```

```
## [1] 0.95 1.01 0.79 1.00 1.14 1.02
```

Moderator Analysis - Part2

Premorbid Language Proficiency

Does proficiency moderate the difference betwen L1 and L2?

Whole group - Overall Performance

```
summary(data$proficiency)
```

```
## equal    L1    L2  NA's
##    63    27     4    25
```

```
data$equal<-ifelse(data$proficiency=="equal",1,0)
data$l1   <-ifelse(data$proficiency=="L1",1,0)
data$l2   <-ifelse(data$proficiency=="L2",1,0)
```

```
print(m16<-rma(yi,vi,mods=cbind(equal,l1,l2),intercept=F,data=data))
```

```
##
## Mixed-Effects Model (k = 94; tau^2 estimator: REML)
##
## tau^2 (estimated amount of residual heterogeneity):     0.0408 (SE = 0.0072)
## tau (square root of estimated tau^2 value):             0.2020
## I^2 (residual heterogeneity / unaccounted variability): 93.98%
## H^2 (unaccounted variability / sampling variability):   16.60
##
## Test for Residual Heterogeneity:
## QE(df = 91) = 709.5533, p-val < .0001
##
## Test of Moderators (coefficient(s) 1:3):
## QM(df = 3) = 27.0105, p-val < .0001
##
## Model Results:
##
##        estimate      se     zval    pval    ci.lb    ci.ub
## equal    0.0609  0.0280   2.1709  0.0299   0.0059   0.1159    *
## l1       0.1465  0.0430   3.4077  0.0007   0.0622   0.2307  ***
## l2      -0.3546  0.1085  -3.2688  0.0011  -0.5672  -0.1420   **
##
## ---
## Signif. codes:  0 '***' 0.001 '**' 0.01 '*' 0.05 '.' 0.1 ' ' 1
```

```
#RR estimate,lower and upper boundaries of the 95% CI
round(exp(c(m16$b,m16$ci.lb,m16$ci.ub)),digits=2)
```

```
## [1] 1.06 1.16 0.70 1.01 1.06 0.57 1.12 1.26 0.87
```

Checking whether proficiency is a significant moderator.

```r
rma(yi,vi,mods=cbind(equal,l1),data=subset(data,proficiency %in% c("equal","L1")))
```

```
##
## Mixed-Effects Model (k = 90; tau^2 estimator: REML)
##
## tau^2 (estimated amount of residual heterogeneity):     0.0391 (SE = 0.0071)
## tau (square root of estimated tau^2 value):             0.1977
## I^2 (residual heterogeneity / unaccounted variability): 93.80%
## H^2 (unaccounted variability / sampling variability):   16.14
## R^2 (amount of heterogeneity accounted for):            2.53%
##
## Test for Residual Heterogeneity:
## QE(df = 88) = 671.3514, p-val < .0001
##
## Test of Moderators (coefficient(s) 2):
## QM(df = 1) = 2.8724, p-val = 0.0901
##
## Model Results:
##
##          estimate      se     zval    pval    ci.lb   ci.ub
## intrcpt    0.1459  0.0422   3.4559  0.0005   0.0631  0.2286  ***
## equal     -0.0854  0.0504  -1.6948  0.0901  -0.1842  0.0134    .
##
## ---
## Signif. codes:  0 '***' 0.001 '**' 0.01 '*' 0.05 '.' 0.1 ' ' 1
```

Whole group - Auditory Comprehension

```
summary(data_ac$proficiency)
```

```
## equal    L1    L2  NA's
##    53    25     4     9
```

```
data_ac$equal<-ifelse(data_ac$proficiency=="equal",1,0)
data_ac$l1   <-ifelse(data_ac$proficiency=="L1",1,0)
data_ac$l2   <-ifelse(data_ac$proficiency=="L2",1,0)
```

```
print(m17<-rma(yi,vi,mods=cbind(equal,l1,l2),intercept=F,data=data_ac))
```

```
##
## Mixed-Effects Model (k = 82; tau^2 estimator: REML)
##
## tau^2 (estimated amount of residual heterogeneity):     0.0219 (SE = 0.0049)
## tau (square root of estimated tau^2 value):             0.1481
## I^2 (residual heterogeneity / unaccounted variability): 86.42%
## H^2 (unaccounted variability / sampling variability):   7.36
##
## Test for Residual Heterogeneity:
## QE(df = 79) = 314.0803, p-val < .0001
##
## Test of Moderators (coefficient(s) 1:3):
## QM(df = 3) = 14.6269, p-val = 0.0022
##
## Model Results:
##
##        estimate      se     zval    pval    ci.lb   ci.ub
## equal    0.0744  0.0246   3.0251  0.0025   0.0262  0.1225  **
## l1       0.0644  0.0371   1.7346  0.0828  -0.0084  0.1371   .
## l2      -0.1301  0.0828  -1.5707  0.1163  -0.2924  0.0322
##
## ---
## Signif. codes:  0 '***' 0.001 '**' 0.01 '*' 0.05 '.' 0.1 ' ' 1
```

```
#RR estimate,lower and upper boundaries of the 95% CI
round(exp(c(m17$b,m17$ci.lb,m17$ci.ub)),digits=2)
```

```
## [1] 1.08 1.07 0.88 1.03 0.99 0.75 1.13 1.15 1.03
```

Checking whether proficiency is a significant moderator.

```
rma(yi,vi,mods=cbind(equal,l1),data=subset(data_ac,proficiency %in% c("equal","L1")))
```

```
##
## Mixed-Effects Model (k = 78; tau^2 estimator: REML)
##
```

```
## tau^2 (estimated amount of residual heterogeneity):     0.0228 (SE = 0.0052)
## tau (square root of estimated tau^2 value):             0.1508
## I^2 (residual heterogeneity / unaccounted variability): 86.98%
## H^2 (unaccounted variability / sampling variability):   7.68
## R^2 (amount of heterogeneity accounted for):            0.00%
##
## Test for Residual Heterogeneity:
## QE(df = 76) = 303.7470, p-val < .0001
##
## Test of Moderators (coefficient(s) 2):
## QM(df = 1) = 0.0528, p-val = 0.8182
##
## Model Results:
##
##          estimate      se    zval    pval    ci.lb   ci.ub
## intrcpt    0.0646  0.0376  1.7163  0.0861  -0.0092  0.1383  .
## equal      0.0104  0.0451  0.2299  0.8182  -0.0781  0.0988
##
## ---
## Signif. codes:  0 '***' 0.001 '**' 0.01 '*' 0.05 '.' 0.1 ' ' 1
```

Whole group - Oral Production

```
summary(data_op$proficiency)
```

```
## equal    L1    L2  NA's
##    52    19     3    17
```

```
data_op$equal<-ifelse(data_op$proficiency=="equal",1,0)
data_op$l1   <-ifelse(data_op$proficiency=="L1",1,0)
data_op$l2   <-ifelse(data_op$proficiency=="L2",1,0)
```

```
print(m18<-rma(yi,vi,mods=cbind(equal,l1,l2),intercept=F,data=data_op))
```

```
##
## Mixed-Effects Model (k = 74; tau^2 estimator: REML)
##
## tau^2 (estimated amount of residual heterogeneity):     0.0594 (SE = 0.0121)
## tau (square root of estimated tau^2 value):             0.2438
## I^2 (residual heterogeneity / unaccounted variability): 92.90%
## H^2 (unaccounted variability / sampling variability):   14.09
##
## Test for Residual Heterogeneity:
## QE(df = 71) = 485.7300, p-val < .0001
##
## Test of Moderators (coefficient(s) 1:3):
## QM(df = 3) = 15.8913, p-val = 0.0012
##
## Model Results:
##
##        estimate      se     zval    pval    ci.lb    ci.ub
## equal    0.0261  0.0376   0.6949  0.4871  -0.0476   0.0998
## l1       0.2032  0.0623   3.2613  0.0011   0.0811   0.3252  **
## l2      -0.3361  0.1538  -2.1845  0.0289  -0.6376  -0.0346   *
##
## ---
## Signif. codes:  0 '***' 0.001 '**' 0.01 '*' 0.05 '.' 0.1 ' ' 1
```

```
#RR estimate,lower and upper boundaries of the 95% CI
round(exp(c(m18$b,m18$ci.lb,m18$ci.ub)),digits=2)
```

```
## [1] 1.03 1.23 0.71 0.95 1.08 0.53 1.10 1.38 0.97
```

Checking whether proficiency is a significant moderator.

```
rma(yi,vi,mods=cbind(equal,l1),data=subset(data_op,proficiency %in% c("equal","L1")))
```

```
##
## Mixed-Effects Model (k = 71; tau^2 estimator: REML)
##
```

```
## tau^2 (estimated amount of residual heterogeneity):     0.0564 (SE = 0.0118)
## tau (square root of estimated tau^2 value):             0.2376
## I^2 (residual heterogeneity / unaccounted variability): 92.65%
## H^2 (unaccounted variability / sampling variability):   13.60
## R^2 (amount of heterogeneity accounted for):            7.60%
##
## Test for Residual Heterogeneity:
## QE(df = 69) = 465.7583, p-val < .0001
##
## Test of Moderators (coefficient(s) 2):
## QM(df = 1) = 6.1317, p-val = 0.0133
##
## Model Results:
##
##          estimate      se     zval    pval    ci.lb    ci.ub
## intrcpt    0.2024  0.0610   3.3199  0.0009   0.0829   0.3219  ***
## equal     -0.1763  0.0712  -2.4762  0.0133  -0.3159  -0.0368    *
##
## ---
## Signif. codes:  0 '***' 0.001 '**' 0.01 '*' 0.05 '.' 0.1 ' ' 1
```

Early subgroup - Overall Performance

```
summary(data_e_overall$proficiency)
```

```
## equal    L1    L2  NA's
##    30     3     4     7
```

```
data_e_overall$equal<-ifelse(data_e_overall$proficiency=="equal",1,0)
data_e_overall$l1   <-ifelse(data_e_overall$proficiency=="L1",1,0)
data_e_overall$l2   <-ifelse(data_e_overall$proficiency=="L2",1,0)
```

```
print(m19<-rma(yi,vi,mods=cbind(equal,l1,l2),intercept=F,data=data_e_overall))
```

```
##
## Mixed-Effects Model (k = 37; tau^2 estimator: REML)
##
## tau^2 (estimated amount of residual heterogeneity):     0.0495 (SE = 0.0137)
## tau (square root of estimated tau^2 value):             0.2224
## I^2 (residual heterogeneity / unaccounted variability): 96.31%
## H^2 (unaccounted variability / sampling variability):   27.12
##
## Test for Residual Heterogeneity:
## QE(df = 34) = 352.6114, p-val < .0001
##
## Test of Moderators (coefficient(s) 1:3):
## QM(df = 3) = 9.8294, p-val = 0.0201
##
## Model Results:
##
##        estimate      se     zval    pval    ci.lb    ci.ub
## equal    0.0320  0.0439   0.7276  0.4669  -0.0542   0.1181
## l1       0.0385  0.1344   0.2865  0.7745  -0.2249   0.3019
## l2      -0.3587  0.1181  -3.0361  0.0024  -0.5903  -0.1271  **
##
## ---
## Signif. codes:  0 '***' 0.001 '**' 0.01 '*' 0.05 '.' 0.1 ' ' 1
```

```
#RR estimate,lower and upper boundaries of the 95% CI
round(exp(c(m19$b,m19$ci.lb,m19$ci.ub)),digits=2)
```

```
## [1] 1.03 1.04 0.70 0.95 0.80 0.55 1.13 1.35 0.88
```

Early subgroup - Auditory Comprehension

```
summary(data_e_ac$proficiency)
```

```
## equal    L1    L2  NA's
##    24     3     4     7
```

```
data_e_ac$equal<-ifelse(data_e_ac$proficiency=="equal",1,0)
data_e_ac$l1   <-ifelse(data_e_ac$proficiency=="L1",1,0)
data_e_ac$l2   <-ifelse(data_e_ac$proficiency=="L2",1,0)
```

```
print(m20<-rma(yi,vi,mods=cbind(equal,l1,l2),intercept=F,data=data_e_ac))
```

```
##
## Mixed-Effects Model (k = 31; tau^2 estimator: REML)
##
## tau^2 (estimated amount of residual heterogeneity):     0.0248 (SE = 0.0087)
## tau (square root of estimated tau^2 value):             0.1574
## I^2 (residual heterogeneity / unaccounted variability): 88.31%
## H^2 (unaccounted variability / sampling variability):   8.55
##
## Test for Residual Heterogeneity:
## QE(df = 28) = 118.2872, p-val < .0001
##
## Test of Moderators (coefficient(s) 1:3):
## QM(df = 3) = 5.1477, p-val = 0.1613
##
## Model Results:
##
##        estimate      se     zval    pval    ci.lb   ci.ub
## equal    0.0625  0.0380   1.6447  0.1000  -0.0120  0.1371
## l1       0.0422  0.0975   0.4324  0.6655  -0.1489  0.2333
## l2      -0.1307  0.0870  -1.5019  0.1331  -0.3012  0.0398
##
## ---
## Signif. codes:  0 '***' 0.001 '**' 0.01 '*' 0.05 '.' 0.1 ' ' 1
```

```
#RR estimate,lower and upper boundaries of the 95% CI
round(exp(c(m20$b,m20$ci.lb,m20$ci.ub)),digits=2)
```

```
## [1] 1.06 1.04 0.88 0.99 0.86 0.74 1.15 1.26 1.04
```

Early subgroup - Oral Production

```r
summary(data_e_op$proficiency)
```

```
## equal    L1    L2
##    27     2     3
```

```r
data_e_op$equal<-ifelse(data_e_op$proficiency=="equal",1,0)
data_e_op$l1   <-ifelse(data_e_op$proficiency=="L1",1,0)
data_e_op$l2   <-ifelse(data_e_op$proficiency=="L2",1,0)
```

```r
print(m21<-rma(yi,vi,mods=cbind(equal,l1,l2),intercept=F,data=data_e_op))
```

```
##
## Mixed-Effects Model (k = 32; tau^2 estimator: REML)
##
## tau^2 (estimated amount of residual heterogeneity):     0.0767 (SE = 0.0231)
## tau (square root of estimated tau^2 value):             0.2769
## I^2 (residual heterogeneity / unaccounted variability): 96.21%
## H^2 (unaccounted variability / sampling variability):   26.42
##
## Test for Residual Heterogeneity:
## QE(df = 29) = 260.7751, p-val < .0001
##
## Test of Moderators (coefficient(s) 1:3):
## QM(df = 3) = 4.0967, p-val = 0.2512
##
## Model Results:
##
##        estimate      se     zval    pval    ci.lb    ci.ub
## equal    0.0114  0.0574   0.1977  0.8433  -0.1012   0.1239
## l1      -0.0406  0.2065  -0.1966  0.8442  -0.4454   0.3642
## l2      -0.3442  0.1717  -2.0047  0.0450  -0.6808  -0.0077  *
##
## ---
## Signif. codes:  0 '***' 0.001 '**' 0.01 '*' 0.05 '.' 0.1 ' ' 1
```

```r
#RR estimate,lower and upper boundaries of the 95% CI
round(exp(c(m21$b,m21$ci.lb,m21$ci.ub)),digits=2)
```

```
## [1] 1.01 0.96 0.71 0.90 0.64 0.51 1.13 1.44 0.99
```

Late subgroup - Overall Performance

```
summary(data_l_overall$proficiency)
```

```
## equal    L1    L2  NA's
##    33    24     0    18
```

```
data_l_overall$equal<-ifelse(data_l_overall$proficiency=="equal",1,0)
data_l_overall$l1   <-ifelse(data_l_overall$proficiency=="L1",1,0)
```

```
print(m22<-rma(yi,vi,mods=cbind(equal,l1),intercept=F,data=data_l_overall))
```

```
##
## Mixed-Effects Model (k = 57; tau^2 estimator: REML)
##
## tau^2 (estimated amount of residual heterogeneity):     0.0355 (SE = 0.0084)
## tau (square root of estimated tau^2 value):             0.1884
## I^2 (residual heterogeneity / unaccounted variability): 90.50%
## H^2 (unaccounted variability / sampling variability):   10.53
##
## Test for Residual Heterogeneity:
## QE(df = 55) = 331.4890, p-val < .0001
##
## Test of Moderators (coefficient(s) 1:2):
## QM(df = 2) = 19.2285, p-val < .0001
##
## Model Results:
##
##        estimate      se    zval    pval   ci.lb   ci.ub
## equal    0.0876  0.0368  2.3802  0.0173  0.0155  0.1596    *
## l1       0.1595  0.0433  3.6828  0.0002  0.0746  0.2444  ***
##
## ---
## Signif. codes:  0 '***' 0.001 '**' 0.01 '*' 0.05 '.' 0.1 ' ' 1
```

```
#RR estimate,lower and upper boundaries of the 95% CI
round(exp(c(m22$b,m22$ci.lb,m22$ci.ub)),digits=2)
```

```
## [1] 1.09 1.17 1.02 1.08 1.17 1.28
```

Checking whether proficiency is a significant moderator.

```
capture.output(rma(yi,vi,mods=cbind(equal,l1),data=data_l_overall))[c(8,10,11,13,14)]
```

```
## [1] "R^2 (amount of heterogeneity accounted for):            1.13%"
## [2] "Test for Residual Heterogeneity: "
## [3] "QE(df = 55) = 331.4890, p-val < .0001"
## [4] "Test of Moderators (coefficient(s) 2): "
## [5] "QM(df = 1) = 1.6033, p-val = 0.2054"
```

Late subgroup - Auditory Comprehension

```
summary(data_l_ac$proficiency)
```

```
## equal    L1    L2  NA's
##    29    22     0     2
```

```
data_l_ac$equal<-ifelse(data_l_ac$proficiency=="equal",1,0)
data_l_ac$l1   <-ifelse(data_l_ac$proficiency=="L1",1,0)
```

```
print(m23<-rma(yi,vi,mods=cbind(equal,l1),intercept=F,data=data_l_ac))
```

```
##
## Mixed-Effects Model (k = 51; tau^2 estimator: REML)
##
## tau^2 (estimated amount of residual heterogeneity):     0.0221 (SE = 0.0065)
## tau (square root of estimated tau^2 value):             0.1488
## I^2 (residual heterogeneity / unaccounted variability): 84.40%
## H^2 (unaccounted variability / sampling variability):   6.41
##
## Test for Residual Heterogeneity:
## QE(df = 49) = 185.1844, p-val < .0001
##
## Test of Moderators (coefficient(s) 1:2):
## QM(df = 2) = 9.4703, p-val = 0.0088
##
## Model Results:
##
##        estimate      se    zval    pval    ci.lb   ci.ub
## equal    0.0862  0.0335  2.5703  0.0102   0.0205  0.1519  *
## l1       0.0688  0.0406  1.6922  0.0906  -0.0109  0.1485  .
##
## ---
## Signif. codes:  0 '***' 0.001 '**' 0.01 '*' 0.05 '.' 0.1 ' ' 1
```

```
#RR estimate,lower and upper boundaries of the 95% CI
round(exp(c(m23$b,m23$ci.lb,m23$ci.ub)),digits=2)
```

```
## [1] 1.09 1.07 1.02 0.99 1.16 1.16
```

Checking whether proficiency is a significant moderator.

```
capture.output(rma(yi,vi,mods=cbind(equal,l1),data=data_l_ac))[c(8,10,11,13,14)]
```

```
## [1] "R^2 (amount of heterogeneity accounted for):            0.00%"
## [2] "Test for Residual Heterogeneity: "
## [3] "QE(df = 49) = 185.1844, p-val < .0001"
## [4] "Test of Moderators (coefficient(s) 2): "
## [5] "QM(df = 1) = 0.1091, p-val = 0.7412"
```

Late subgroup - Oral Production

```
summary(data_l_op$proficiency)
```

```
## equal    L1    L2  NA's
##    25    17     0    17
```

```
data_l_op$equal<-ifelse(data_l_op$proficiency=="equal",1,0)
data_l_op$l1   <-ifelse(data_l_op$proficiency=="L1",1,0)
```

```
print(m24<-rma(yi,vi,mods=cbind(equal,l1),intercept=F,data=data_l_op))
```

```
##
## Mixed-Effects Model (k = 42; tau^2 estimator: REML)
##
## tau^2 (estimated amount of residual heterogeneity):     0.0476 (SE = 0.0138)
## tau (square root of estimated tau^2 value):             0.2182
## I^2 (residual heterogeneity / unaccounted variability): 85.17%
## H^2 (unaccounted variability / sampling variability):   6.74
##
## Test for Residual Heterogeneity:
## QE(df = 40) = 200.2290, p-val < .0001
##
## Test of Moderators (coefficient(s) 1:2):
## QM(df = 2) = 15.4474, p-val = 0.0004
##
## Model Results:
##
##        estimate      se    zval    pval    ci.lb   ci.ub
## equal    0.0465  0.0505  0.9201  0.3575  -0.0526  0.1456
## l1       0.2312  0.0605  3.8211  0.0001   0.1126  0.3499  ***
##
## ---
## Signif. codes:  0 '***' 0.001 '**' 0.01 '*' 0.05 '.' 0.1 ' ' 1
```

```
#RR estimate,lower and upper boundaries of the 95% CI
round(exp(c(m24$b,m24$ci.lb,m24$ci.ub)),digits=2)
```

```
## [1] 1.05 1.26 0.95 1.12 1.16 1.42
```

Checking whether proficiency is a significant moderator.

```
capture.output(rma(yi,vi,mods=cbind(equal,l1),data=data_l_op))[c(8,10,11,13,14)]
```

```
## [1] "R^2 (amount of heterogeneity accounted for):            9.24%"
## [2] "Test for Residual Heterogeneity: "
## [3] "QE(df = 40) = 200.2290, p-val < .0001"
## [4] "Test of Moderators (coefficient(s) 2): "
## [5] "QM(df = 1) = 5.4900, p-val = 0.0191"
```

Language Use

Does language use moderate the difference betwen L1 and L2?

Whole group - Overall Performance

```
summary(data$language_use)
```

```
## equal    L1    L2  NA's
##    52    27    24    16
```

```
data$equal.use<-ifelse(data$language_use=="equal",1,0)
data$l1.use   <-ifelse(data$language_use=="L1",1,0)
data$l2.use   <-ifelse(data$language_use=="L2",1,0)
```

```
print(m25<-rma(yi,vi,mods=cbind(equal.use,l1.use,l2.use),intercept=F,data=data))
```

```
##
## Mixed-Effects Model (k = 103; tau^2 estimator: REML)
##
## tau^2 (estimated amount of residual heterogeneity):     0.0451 (SE = 0.0075)
## tau (square root of estimated tau^2 value):             0.2125
## I^2 (residual heterogeneity / unaccounted variability): 94.26%
## H^2 (unaccounted variability / sampling variability):   17.42
##
## Test for Residual Heterogeneity:
## QE(df = 100) = 860.1528, p-val < .0001
##
## Test of Moderators (coefficient(s) 1:3):
## QM(df = 3) = 22.0380, p-val < .0001
##
## Model Results:
##
##            estimate      se     zval    pval    ci.lb   ci.ub
## equal.use    0.0819  0.0322   2.5418  0.0110   0.0187  0.1450    *
## l1.use       0.1743  0.0462   3.7727  0.0002   0.0837  0.2648  ***
## l2.use      -0.0530  0.0457  -1.1594  0.2463  -0.1425  0.0366
##
## ---
## Signif. codes:  0 '***' 0.001 '**' 0.01 '*' 0.05 '.' 0.1 ' ' 1
```

```
#RR estimate,lower and upper boundaries of the 95% CI
round(exp(c(m25$b,m25$ci.lb,m25$ci.ub)),digits=2)
```

```
## [1] 1.09 1.19 0.95 1.02 1.09 0.87 1.16 1.30 1.04
```

Checking whether language use is a significant moderator.

```r
capture.output(rma(yi,vi,mods=cbind(equal.use,l1.use,l2.use),data=data))[8:24]
```

```
##  [1] "R^2 (amount of heterogeneity accounted for):            10.66%"
##  [2] ""
##  [3] "Test for Residual Heterogeneity: "
##  [4] "QE(df = 100) = 860.1528, p-val < .0001"
##  [5] ""
##  [6] "Test of Moderators (coefficient(s) 2:3): "
##  [7] "QM(df = 2) = 12.4754, p-val = 0.0020"
##  [8] ""
##  [9] "Model Results:"
## [10] ""
## [11] "            estimate      se     zval    pval    ci.lb   ci.ub     "
## [12] "intrcpt     -0.0530  0.0457  -1.1594  0.2463  -0.1425  0.0366     "
## [13] "equal.use    0.1348  0.0559   2.4122  0.0159   0.0253  0.2444    *"
## [14] "l1.use       0.2272  0.0650   3.4977  0.0005   0.0999  0.3546  ***"
## [15] ""
## [16] "---"
## [17] "Signif. codes:  0 '***' 0.001 '**' 0.01 '*' 0.05 '.' 0.1 ' ' 1 "
```

```r
capture.output(rma(yi,vi,mods=~relevel(factor(language_use),ref="equal"),data=data))[8:24]
```

```
##  [1] "R^2 (amount of heterogeneity accounted for):            10.66%"
##  [2] ""
##  [3] "Test for Residual Heterogeneity: "
##  [4] "QE(df = 100) = 860.1528, p-val < .0001"
##  [5] ""
##  [6] "Test of Moderators (coefficient(s) 2:3): "
##  [7] "QM(df = 2) = 12.4754, p-val = 0.0020"
##  [8] ""
##  [9] "Model Results:"
## [10] ""
## [11] "                                               estimate      se     zval    pval    ci.lb   [illegible]
## [12] "intrcpt                                        0.0819  0.0322   2.5418  0.0110   0.0187   0[illegible]
## [13] "relevel(factor(language_use), ref = \"equal\")L1    0.0924  0.0563   1.6411  0.1008  -0.0180   [illegible]
## [14] "relevel(factor(language_use), ref = \"equal\")L2   -0.1348  0.0559  -2.4122  0.0159  -0.2444  [illegible]
## [15] ""
## [16] "---"
## [17] "Signif. codes:  0 '***' 0.001 '**' 0.01 '*' 0.05 '.' 0.1 ' ' 1 "
```

Whole group - Auditory Comprehension

```
summary(data_ac$language_use)
```

```
## equal    L1    L2  NA's
##    34    22    23    12
```

```
data_ac$equal.use<-ifelse(data_ac$language_use=="equal",1,0)
data_ac$l1.use   <-ifelse(data_ac$language_use=="L1",1,0)
data_ac$l2.use   <-ifelse(data_ac$language_use=="L2",1,0)
```

```
print(m26<-rma(yi,vi,mods=cbind(equal.use,l1.use,l2.use),intercept=F,data=data_ac))
```

```
##
## Mixed-Effects Model (k = 79; tau^2 estimator: REML)
##
## tau^2 (estimated amount of residual heterogeneity):     0.0154 (SE = 0.0037)
## tau (square root of estimated tau^2 value):             0.1243
## I^2 (residual heterogeneity / unaccounted variability): 81.79%
## H^2 (unaccounted variability / sampling variability):   5.49
##
## Test for Residual Heterogeneity:
## QE(df = 76) = 279.0789, p-val < .0001
##
## Test of Moderators (coefficient(s) 1:3):
## QM(df = 3) = 11.3056, p-val = 0.0102
##
## Model Results:
##
##            estimate      se     zval    pval    ci.lb   ci.ub
## equal.use    0.0460  0.0268   1.7183  0.0857  -0.0065  0.0984   .
## l1.use       0.1022  0.0359   2.8425  0.0045   0.0317  0.1726  **
## l2.use      -0.0156  0.0299  -0.5226  0.6012  -0.0743  0.0430
##
## ---
## Signif. codes:  0 '***' 0.001 '**' 0.01 '*' 0.05 '.' 0.1 ' ' 1
```

```
#RR estimate  lower and upper boundaries of the 95% CI.
round(exp(c(m26$b,m26$ci.lb,m26$ci.ub)),digits=2)
```

```
## [1] 1.05 1.11 0.98 0.99 1.03 0.93 1.10 1.19 1.04
```

Checking whether language use is a significant moderator.

```
capture.output(rma(yi,vi,mods=cbind(equal.use,l1.use,l2.use),data=data_ac))[8:24]
```

```
##  [1] "R^2 (amount of heterogeneity accounted for):            8.31%"
##  [2] ""
##  [3] "Test for Residual Heterogeneity: "
```

```
##  [4] "QE(df = 76) = 279.0789, p-val < .0001"
##  [5] ""
##  [6] "Test of Moderators (coefficient(s) 2:3): "
##  [7] "QM(df = 2) = 6.4862, p-val = 0.0390"
##  [8] ""
##  [9] "Model Results:"
## [10] ""
## [11] "           estimate      se     zval    pval    ci.lb   ci.ub   "
## [12] "intrcpt     -0.0156  0.0299  -0.5226  0.6012  -0.0743  0.0430   "
## [13] "equal.use    0.0616  0.0402   1.5346  0.1249  -0.0171  0.1403   "
## [14] "l1.use       0.1178  0.0468   2.5185  0.0118   0.0261  0.2095  *"
## [15] ""
## [16] "---"
## [17] "Signif. codes:  0 '***' 0.001 '**' 0.01 '*' 0.05 '.' 0.1 ' ' 1 "
```

```
capture.output(rma(yi,vi,mods=~relevel(factor(language_use),ref="equal"),data=data_ac))[8:24]
```

```
##  [1] "R^2 (amount of heterogeneity accounted for):            8.31%"
##  [2] ""
##  [3] "Test for Residual Heterogeneity: "
##  [4] "QE(df = 76) = 279.0789, p-val < .0001"
##  [5] ""
##  [6] "Test of Moderators (coefficient(s) 2:3): "
##  [7] "QM(df = 2) = 6.4862, p-val = 0.0390"
##  [8] ""
##  [9] "Model Results:"
## [10] ""
## [11] "                                               estimate      se     zval    pval    ci.lb   c
## [12] "intrcpt                                          0.0460  0.0268   1.7183  0.0857  -0.0065  0.
## [13] "relevel(factor(language_use), ref = \"equal\")L1    0.0562  0.0448   1.2540  0.2098  -0.0316
## [14] "relevel(factor(language_use), ref = \"equal\")L2   -0.0616  0.0402  -1.5346  0.1249  -0.1403
## [15] ""
## [16] "---"
## [17] "Signif. codes:  0 '***' 0.001 '**' 0.01 '*' 0.05 '.' 0.1 ' ' 1 "
```

Whole group - Oral Production

```
summary(data_op$language_use)
```

```
## equal    L1    L2  NA's
##    41    17    21    12
```

```
data_op$equal.use<-ifelse(data_op$language_use=="equal",1,0)
data_op$l1.use   <-ifelse(data_op$language_use=="L1",1,0)
data_op$l2.use   <-ifelse(data_op$language_use=="L2",1,0)
```

```
print(m27<-rma(yi,vi,mods=cbind(equal.use,l1.use,l2.use),intercept=F,data=data_op))
```

```
##
## Mixed-Effects Model (k = 79; tau^2 estimator: REML)
##
## tau^2 (estimated amount of residual heterogeneity):     0.0791 (SE = 0.0150)
## tau (square root of estimated tau^2 value):             0.2813
## I^2 (residual heterogeneity / unaccounted variability): 93.94%
## H^2 (unaccounted variability / sampling variability):   16.50
##
## Test for Residual Heterogeneity:
## QE(df = 76) = 595.2075, p-val < .0001
##
## Test of Moderators (coefficient(s) 1:3):
## QM(df = 3) = 11.4454, p-val = 0.0095
##
## Model Results:
##
##            estimate      se     zval    pval    ci.lb   ci.ub
## equal.use    0.0640  0.0481   1.3299  0.1836  -0.0303  0.1583
## l1.use       0.2323  0.0748   3.1042  0.0019   0.0856  0.3790  **
## l2.use      -0.0132  0.0653  -0.2023  0.8397  -0.1412  0.1148
##
## ---
## Signif. codes:  0 '***' 0.001 '**' 0.01 '*' 0.05 '.' 0.1 ' ' 1
```

```
#RR estimate  lower and upper boundaries of the 95% CI.
round(exp(c(m27$b,m27$ci.lb,m27$ci.ub)),digits=2)
```

```
## [1] 1.07 1.26 0.99 0.97 1.09 0.87 1.17 1.46 1.12
```

Checking whether language use is a significant moderator.

```
capture.output(rma(yi,vi,mods=cbind(equal.use,l1.use,l2.use),data=data_op))[8:24]
```

```
##  [1] "R^2 (amount of heterogeneity accounted for):            5.88%"
##  [2] ""
##  [3] "Test for Residual Heterogeneity: "
```

```
## [4] "QE(df = 76) = 595.2075, p-val < .0001"
## [5] ""
## [6] "Test of Moderators (coefficient(s) 2:3): "
## [7] "QM(df = 2) = 6.2857, p-val = 0.0432"
## [8] ""
## [9] "Model Results:"
## [10] ""
## [11] "           estimate      se     zval    pval    ci.lb   ci.ub   "
## [12] "intrcpt     -0.0132  0.0653  -0.2023  0.8397  -0.1412  0.1148   "
## [13] "equal.use    0.0772  0.0811   0.9516  0.3413  -0.0818  0.2362   "
## [14] "l1.use       0.2455  0.0993   2.4716  0.0135   0.0508  0.4402  *"
## [15] ""
## [16] "---"
## [17] "Signif. codes:  0 '***' 0.001 '**' 0.01 '*' 0.05 '.' 0.1 ' ' 1 "
```

```
capture.output(rma(yi,vi,mods=~relevel(factor(language_use),ref="equal"), data=data_op))[8:24]
```

```
## [1] "R^2 (amount of heterogeneity accounted for):           5.88%"
## [2] ""
## [3] "Test for Residual Heterogeneity: "
## [4] "QE(df = 76) = 595.2075, p-val < .0001"
## [5] ""
## [6] "Test of Moderators (coefficient(s) 2:3): "
## [7] "QM(df = 2) = 6.2857, p-val = 0.0432"
## [8] ""
## [9] "Model Results:"
## [10] ""
## [11] "                                                estimate      se     zval    pval    ci.lb   c
## [12] "intrcpt                                           0.0640  0.0481   1.3299  0.1836  -0.0303  0.
## [13] "relevel(factor(language_use), ref = \"equal\")L1    0.1683  0.0890   1.8917  0.0585  -0.0061  
## [14] "relevel(factor(language_use), ref = \"equal\")L2   -0.0772  0.0811  -0.9516  0.3413  -0.2362  
## [15] ""
## [16] "---"
## [17] "Signif. codes:  0 '***' 0.001 '**' 0.01 '*' 0.05 '.' 0.1 ' ' 1 "
```

Early subgroup - Overall Performance

```
summary(data_e_overall$language_use)
```

```
## equal    L1    L2  NA's
##    21     5    13     5
```

```
data_e_overall$equal.use<-ifelse(data_e_overall$language_use=="equal",1,0)
data_e_overall$l1.use   <-ifelse(data_e_overall$language_use=="L1",1,0)
data_e_overall$l2.use   <-ifelse(data_e_overall$language_use=="L2",1,0)
```

```
print(m28<-rma(yi,vi,mods=cbind(equal.use,l1.use,l2.use),intercept=F,data=data_e_overall))
```

```
##
## Mixed-Effects Model (k = 39; tau^2 estimator: REML)
##
## tau^2 (estimated amount of residual heterogeneity):     0.0469 (SE = 0.0126)
## tau (square root of estimated tau^2 value):             0.2165
## I^2 (residual heterogeneity / unaccounted variability): 95.94%
## H^2 (unaccounted variability / sampling variability):   24.64
##
## Test for Residual Heterogeneity:
## QE(df = 36) = 324.7402, p-val < .0001
##
## Test of Moderators (coefficient(s) 1:3):
## QM(df = 3) = 7.2285, p-val = 0.0650
##
## Model Results:
##
##            estimate      se     zval    pval    ci.lb    ci.ub
## equal.use    0.0390  0.0498   0.7820  0.4342  -0.0587   0.1366
## l1.use       0.1261  0.1154   1.0930  0.2744  -0.1001   0.3523
## l2.use      -0.1487  0.0639  -2.3286  0.0199  -0.2739  -0.0235  *
##
## ---
## Signif. codes:  0 '***' 0.001 '**' 0.01 '*' 0.05 '.' 0.1 ' ' 1
```

```
#RR estimate  lower and upper boundaries of the 95% CI
round(exp(c(m28$b,m28$ci.lb,m28$ci.ub)),digits=2)
```

```
## [1] 1.04 1.13 0.86 0.94 0.90 0.76 1.15 1.42 0.98
```

Checking whether language use is a significant moderator.

```
data_e_overall_2<-subset(data_e_overall,language_use %in% c("equal","L2"))
capture.output(rma(yi,vi,mods=cbind(equal.use,l2.use),data=data_e_overall_2))[c(8,10,11,13,14)]
```

```
## [1] "R^2 (amount of heterogeneity accounted for):            12.77%"
## [2] "Test for Residual Heterogeneity: "
## [3] "QE(df = 32) = 316.4152, p-val < .0001"
## [4] "Test of Moderators (coefficient(s) 2): "
## [5] "QM(df = 1) = 5.1410, p-val = 0.0234"
```

Early subgroup - Auditory Comprehension

```
summary(data_e_ac$language_use)
```

```
## equal    L1    L2  NA's
##    19     3    12     4
```

```
data_e_ac$equal.use<-ifelse(data_e_ac$language_use=="equal",1,0)
data_e_ac$l1.use   <-ifelse(data_e_ac$language_use=="L1",1,0)
data_e_ac$l2.use   <-ifelse(data_e_ac$language_use=="L2",1,0)
```

```
print(m29<-rma(yi,vi,mods=cbind(equal.use,l1.use,l2.use),intercept=F,data=data_e_ac))
```

```
##
## Mixed-Effects Model (k = 34; tau^2 estimator: REML)
##
## tau^2 (estimated amount of residual heterogeneity):     0.0090 (SE = 0.0038)
## tau (square root of estimated tau^2 value):             0.0947
## I^2 (residual heterogeneity / unaccounted variability): 71.65%
## H^2 (unaccounted variability / sampling variability):   3.53
##
## Test for Residual Heterogeneity:
## QE(df = 31) = 86.5203, p-val < .0001
##
## Test of Moderators (coefficient(s) 1:3):
## QM(df = 3) = 6.8236, p-val = 0.0777
##
## Model Results:
##
##            estimate      se     zval    pval    ci.lb   ci.ub
## equal.use    0.0542  0.0296   1.8333  0.0668  -0.0037  0.1121  .
## l1.use      -0.0307  0.0793  -0.3865  0.6991  -0.1861  0.1248
## l2.use      -0.0624  0.0343  -1.8202  0.0687  -0.1296  0.0048  .
##
## ---
## Signif. codes:  0 '***' 0.001 '**' 0.01 '*' 0.05 '.' 0.1 ' ' 1
```

```
#RR estimate  lower and upper boundaries of the 95% CI.
round(exp(c(m29$b,m29$ci.lb,m29$ci.ub)),digits=2)
```

```
## [1] 1.06 0.97 0.94 1.00 0.83 0.88 1.12 1.13 1.00
```

Checking whether language use is a significant moderator.

```
data_e_ac_2<-subset(data_e_ac,language_use %in% c("equal","L2"))
capture.output(rma(yi,vi,mods=cbind(equal.use,l2.use),data=data_e_ac_2))[c(8,10,11,13,14)]
```

```
## [1] "R^2 (amount of heterogeneity accounted for):            30.05%"
## [2] "Test for Residual Heterogeneity: "
## [3] "QE(df = 29) = 83.1714, p-val < .0001"
## [4] "Test of Moderators (coefficient(s) 2): "
## [5] "QM(df = 1) = 6.5660, p-val = 0.0104"
```

Early subgroup - Oral Production

```r
summary(data_e_op$language_use)
```

```
## equal    L1    L2  NA's
##    14     4    10     4
```

```r
data_e_op$equal.use<-ifelse(data_e_op$language_use=="equal",1,0)
data_e_op$l1.use   <-ifelse(data_e_op$language_use=="L1",1,0)
data_e_op$l2.use   <-ifelse(data_e_op$language_use=="L2",1,0)
```

```r
print(m30<-rma(yi,vi,mods=cbind(equal.use,l1.use,l2.use),intercept=F,data=data_e_op))
```

```
##
## Mixed-Effects Model (k = 28; tau^2 estimator: REML)
##
## tau^2 (estimated amount of residual heterogeneity):     0.0904 (SE = 0.0286)
## tau (square root of estimated tau^2 value):             0.3007
## I^2 (residual heterogeneity / unaccounted variability): 96.65%
## H^2 (unaccounted variability / sampling variability):   29.88
##
## Test for Residual Heterogeneity:
## QE(df = 25) = 228.6500, p-val < .0001
##
## Test of Moderators (coefficient(s) 1:3):
## QM(df = 3) = 2.8521, p-val = 0.4150
##
## Model Results:
##
##            estimate      se     zval    pval    ci.lb   ci.ub
## equal.use   -0.0270  0.0848  -0.3179  0.7505  -0.1933  0.1393
## l1.use       0.1893  0.1716   1.1034  0.2699  -0.1470  0.5256
## l2.use      -0.1228  0.0991  -1.2384  0.2156  -0.3170  0.0715
##
## ---
## Signif. codes:  0 '***' 0.001 '**' 0.01 '*' 0.05 '.' 0.1 ' ' 1
```

```r
#RR estimate  lower and upper boundaries of the 95% CI.
round(exp(c(m30$b,m30$ci.lb,m30$ci.ub)),digits=2)
```

```
## [1] 0.97 1.21 0.88 0.82 0.86 0.73 1.15 1.69 1.07
```

Checking whether language use is a significant moderator.

```r
data_e_op_2<-subset(data_e_op,language_use %in% c("equal","L2"))
capture.output(rma(yi,vi,mods=cbind(equal.use,l2.use),data=data_e_op_2))[c(8,10,11,13,14)]
```

```
## [1] "R^2 (amount of heterogeneity accounted for):            0.00%"
## [2] "Test for Residual Heterogeneity: "
## [3] "QE(df = 22) = 222.0485, p-val < .0001"
## [4] "Test of Moderators (coefficient(s) 2): "
## [5] "QM(df = 1) = 0.5006, p-val = 0.4792"
```

Late subgroup - Overall Performance

```r
summary(data_l_overall$language_use)
```

```
## equal    L1    L2  NA's
##    31    22    11    11
```

```r
data_l_overall$equal.use<-ifelse(data_l_overall$language_use=="equal",1,0)
data_l_overall$l1.use   <-ifelse(data_l_overall$language_use=="L1",1,0)
data_l_overall$l2.use   <-ifelse(data_l_overall$language_use=="L2",1,0)
```

```r
print(m31<-rma(yi,vi,mods=cbind(equal.use,l1.use,l2.use),intercept=F,data=data_l_overall))
```

```
##
## Mixed-Effects Model (k = 64; tau^2 estimator: REML)
##
## tau^2 (estimated amount of residual heterogeneity):     0.0394 (SE = 0.0087)
## tau (square root of estimated tau^2 value):             0.1984
## I^2 (residual heterogeneity / unaccounted variability): 90.94%
## H^2 (unaccounted variability / sampling variability):   11.04
##
## Test for Residual Heterogeneity:
## QE(df = 61) = 421.2807, p-val < .0001
##
## Test of Moderators (coefficient(s) 1:3):
## QM(df = 3) = 23.3657, p-val < .0001
##
## Model Results:
##
##            estimate      se    zval    pval    ci.lb   ci.ub
## equal.use    0.1149  0.0404  2.8428  0.0045   0.0357  0.1941   **
## l1.use       0.1820  0.0477  3.8119  0.0001   0.0884  0.2756  ***
## l2.use       0.0542  0.0625  0.8681  0.3854  -0.0682  0.1767
##
## ---
## Signif. codes:  0 '***' 0.001 '**' 0.01 '*' 0.05 '.' 0.1 ' ' 1
```

```r
#RR estimate  lower and upper boundaries of the 95% CI
round(exp(c(m31$b,m31$ci.lb,m31$ci.ub)),digits=2)
```

```
## [1] 1.12 1.20 1.06 1.04 1.09 0.93 1.21 1.32 1.19
```

Checking whether language use is a significant moderator.

```r
capture.output(rma(yi,vi,mods=cbind(equal.use,l1.use,l2.use),data=data_l_overall))[c(8,10,11,13,14)]
```

```
## [1] "R^2 (amount of heterogeneity accounted for):            0.00%"
## [2] "Test for Residual Heterogeneity: "
## [3] "QE(df = 61) = 421.2807, p-val < .0001"
## [4] "Test of Moderators (coefficient(s) 2:3): "
## [5] "QM(df = 2) = 2.7691, p-val = 0.2504"
```

Late subgroup - Auditory Comprehension

```
summary(data_l_ac$language_use)
```

```
## equal    L1    L2  NA's
##    15    19    11     8
```

```
data_l_ac$equal.use<-ifelse(data_l_ac$language_use=="equal",1,0)
data_l_ac$l1.use   <-ifelse(data_l_ac$language_use=="L1",1,0)
data_l_ac$l2.use   <-ifelse(data_l_ac$language_use=="L2",1,0)
```

```
print(m32<-rma(yi,vi,mods=cbind(equal.use,l1.use,l2.use),intercept=F,data=data_l_ac))
```

```
##
## Mixed-Effects Model (k = 45; tau^2 estimator: REML)
##
## tau^2 (estimated amount of residual heterogeneity):     0.0182 (SE = 0.0058)
## tau (square root of estimated tau^2 value):             0.1351
## I^2 (residual heterogeneity / unaccounted variability): 82.56%
## H^2 (unaccounted variability / sampling variability):   5.73
##
## Test for Residual Heterogeneity:
## QE(df = 42) = 158.2786, p-val < .0001
##
## Test of Moderators (coefficient(s) 1:3):
## QM(df = 3) = 10.3308, p-val = 0.0160
##
## Model Results:
##
##            estimate      se    zval    pval    ci.lb   ci.ub
## equal.use    0.0268  0.0431  0.6216  0.5342  -0.0577  0.1113
## l1.use       0.1260  0.0411  3.0665  0.0022   0.0455  0.2065  **
## l2.use       0.0336  0.0457  0.7355  0.4621  -0.0559  0.1231
##
## ---
## Signif. codes:  0 '***' 0.001 '**' 0.01 '*' 0.05 '.' 0.1 ' ' 1
```

```
#RR estimate  lower and upper boundaries of the 95% CI
round(exp(c(m32$b,m32$ci.lb,m32$ci.ub)),digits=2)
```

```
## [1] 1.03 1.13 1.03 0.94 1.05 0.95 1.12 1.23 1.13
```

Checking whether language use is a significant moderator.

```
capture.output(rma(yi,vi,mods=cbind(equal.use,l1.use,l2.use),data=data_l_ac))[c(8,10,11,13,14)]
```

```
## [1] "R^2 (amount of heterogeneity accounted for):            0.83%"
## [2] "Test for Residual Heterogeneity: "
## [3] "QE(df = 42) = 158.2786, p-val < .0001"
## [4] "Test of Moderators (coefficient(s) 2:3): "
## [5] "QM(df = 2) = 3.4625, p-val = 0.1771"
```

Late subgroup - Oral Production

```
summary(data_l_op$language_use)
```

```
## equal    L1    L2  NA's
##    27    13    11     8
```

```
data_l_op$equal.use<-ifelse(data_l_op$language_use=="equal",1,0)
data_l_op$l1.use   <-ifelse(data_l_op$language_use=="L1",1,0)
data_l_op$l2.use   <-ifelse(data_l_op$language_use=="L2",1,0)
```

```
print(m33<-rma(yi,vi,mods=cbind(equal.use,l1.use,l2.use),intercept=F,data=data_l_op))
```

```
##
## Mixed-Effects Model (k = 51; tau^2 estimator: REML)
##
## tau^2 (estimated amount of residual heterogeneity):     0.0676 (SE = 0.0168)
## tau (square root of estimated tau^2 value):             0.2600
## I^2 (residual heterogeneity / unaccounted variability): 88.64%
## H^2 (unaccounted variability / sampling variability):   8.80
##
## Test for Residual Heterogeneity:
## QE(df = 48) = 291.5760, p-val < .0001
##
## Test of Moderators (coefficient(s) 1:3):
## QM(df = 3) = 15.1497, p-val = 0.0017
##
## Model Results:
##
##            estimate      se    zval    pval    ci.lb   ci.ub
## equal.use    0.1181  0.0565  2.0917  0.0365   0.0074  0.2288   *
## l1.use       0.2442  0.0787  3.1008  0.0019   0.0898  0.3985  **
## l2.use       0.0921  0.0855  1.0768  0.2816  -0.0755  0.2597
##
## ---
## Signif. codes:  0 '***' 0.001 '**' 0.01 '*' 0.05 '.' 0.1 ' ' 1
```

```
#RR estimate  lower and upper boundaries of the 95% CI
round(exp(c(m33$b,m33$ci.lb,m33$ci.ub)),digits=2)
```

```
## [1] 1.13 1.28 1.10 1.01 1.09 0.93 1.26 1.49 1.30
```

Checking whether language use is a significant moderator.

```
capture.output(rma(yi,vi,mods=cbind(equal.use,l1.use,l2.use),data=data_l_op))[c(8,10,11,13,14)]
```

```
## [1] "R^2 (amount of heterogeneity accounted for):            0.00%"
## [2] "Test for Residual Heterogeneity: "
## [3] "QE(df = 48) = 291.5760, p-val < .0001"
## [4] "Test of Moderators (coefficient(s) 2:3): "
## [5] "QM(df = 2) = 2.1951, p-val = 0.3337"
```

Linguistic Similarity

Does 2-level linguistic similarity moderate the difference betwen L1 and L2?

Whole group - Overall Performance

```
summary(data$ling_similar_2levels)
```

```
## different   similar
##        29        90
```

```
data$similar_binary  <-ifelse(data$ling_similar_2levels=="similar",1,0)
data$different_binary<-ifelse(data$ling_similar_2levels=="different",1,0)
```

```
print(m34<-rma(yi,vi,mods=cbind(similar_binary,different_binary),intercept=F,data=data))
```

```
##
## Mixed-Effects Model (k = 119; tau^2 estimator: REML)
##
## tau^2 (estimated amount of residual heterogeneity):     0.0525 (SE = 0.0080)
## tau (square root of estimated tau^2 value):             0.2290
## I^2 (residual heterogeneity / unaccounted variability): 94.87%
## H^2 (unaccounted variability / sampling variability):   19.50
##
## Test for Residual Heterogeneity:
## QE(df = 117) = 1012.9185, p-val < .0001
##
## Test of Moderators (coefficient(s) 1:2):
## QM(df = 2) = 16.8746, p-val = 0.0002
##
## Model Results:
##
##                   estimate      se    zval    pval   ci.lb   ci.ub
## similar_binary      0.0852  0.0264  3.2261  0.0013  0.0334  0.1370  **
## different_binary    0.1168  0.0459  2.5430  0.0110  0.0268  0.2067   *
##
## ---
## Signif. codes:  0 '***' 0.001 '**' 0.01 '*' 0.05 '.' 0.1 ' ' 1
```

```
#RR estimate  lower and upper boundaries of the 95% CI
round(exp(c(m34$b,m34$ci.lb,m34$ci.ub)),digits=2)
```

```
## [1] 1.09 1.12 1.03 1.03 1.15 1.23
```

Checking whether linguistic similarity is a significant moderator.

```
capture.output(rma(yi,vi,mods=cbind(similar_binary,different_binary),data=data))[c(8,10,11,13,14)]
```

```
## [1] "R^2 (amount of heterogeneity accounted for):            0.00%"
## [2] "Test for Residual Heterogeneity: "
## [3] "QE(df = 117) = 1012.9185, p-val < .0001"
## [4] "Test of Moderators (coefficient(s) 2): "
## [5] "QM(df = 1) = 0.3544, p-val = 0.5516"
```

Whole group - Auditory Comprehension

```r
summary(data_ac$ling_similar_2levels)
```

```
## different   similar
##        25        66
```

```r
data_ac$similar_binary  <-ifelse(data_ac$ling_similar_2levels=="similar",1,0)
data_ac$different_binary<-ifelse(data_ac$ling_similar_2levels=="different",1,0)
```

```r
print(m35<-rma(yi,vi,mods=cbind(similar_binary,different_binary),intercept=F,data=data_ac))
```

```
##
## Mixed-Effects Model (k = 91; tau^2 estimator: REML)
##
## tau^2 (estimated amount of residual heterogeneity):     0.0229 (SE = 0.0048)
## tau (square root of estimated tau^2 value):             0.1514
## I^2 (residual heterogeneity / unaccounted variability): 86.82%
## H^2 (unaccounted variability / sampling variability):   7.59
##
## Test for Residual Heterogeneity:
## QE(df = 89) = 362.7039, p-val < .0001
##
## Test of Moderators (coefficient(s) 1:2):
## QM(df = 2) = 10.6498, p-val = 0.0049
##
## Model Results:
##
##                   estimate      se    zval    pval   ci.lb   ci.ub
## similar_binary      0.0530  0.0227  2.3405  0.0193  0.0086  0.0974  *
## different_binary    0.0813  0.0358  2.2742  0.0230  0.0112  0.1514  *
##
## ---
## Signif. codes:  0 '***' 0.001 '**' 0.01 '*' 0.05 '.' 0.1 ' ' 1
```

```r
#RR estimate  lower and upper boundaries of the 95% CI
round(exp(c(m35$b,m35$ci.lb,m35$ci.ub)),digits=2)
```

```
## [1] 1.05 1.08 1.01 1.01 1.10 1.16
```

Checking whether linguistic similarity is a significant moderator.

```r
capture.output(rma(yi,vi,mods=cbind(similar_binary,different_binary),data=data_ac))[c(8,10,11,13,14)]
```

```
## [1] "R^2 (amount of heterogeneity accounted for):            0.00%"
## [2] "Test for Residual Heterogeneity: "
## [3] "QE(df = 89) = 362.7039, p-val < .0001"
## [4] "Test of Moderators (coefficient(s) 2): "
## [5] "QM(df = 1) = 0.4467, p-val = 0.5039"
```

Whole group - Oral Production

```
summary(data_op$ling_similar_2levels)
```

```
## different   similar
##        18        73
```

```
data_op$similar_binary  <-ifelse(data_op$ling_similar_2levels=="similar",1,0)
data_op$different_binary<-ifelse(data_op$ling_similar_2levels=="different",1,0)
```

```
print(m36<-rma(yi,vi,mods=cbind(similar_binary,different_binary),intercept=F,data=data_op))
```

```
##
## Mixed-Effects Model (k = 91; tau^2 estimator: REML)
##
## tau^2 (estimated amount of residual heterogeneity):     0.0778 (SE = 0.0137)
## tau (square root of estimated tau^2 value):             0.2790
## I^2 (residual heterogeneity / unaccounted variability): 93.81%
## H^2 (unaccounted variability / sampling variability):   16.14
##
## Test for Residual Heterogeneity:
## QE(df = 89) = 682.3731, p-val < .0001
##
## Test of Moderators (coefficient(s) 1:2):
## QM(df = 2) = 8.3664, p-val = 0.0152
##
## Model Results:
##
##                     estimate      se    zval    pval    ci.lb   ci.ub
## similar_binary        0.0959  0.0359  2.6743  0.0075   0.0256  0.1663  **
## different_binary      0.0772  0.0701  1.1020  0.2705  -0.0601  0.2146
##
## ---
## Signif. codes:  0 '***' 0.001 '**' 0.01 '*' 0.05 '.' 0.1 ' ' 1
```

```
#RR estimate  lower and upper boundaries of the 95% CI
round(exp(c(m36$b,m36$ci.lb,m36$ci.ub)),digits=2)
```

```
## [1] 1.10 1.08 1.03 0.94 1.18 1.24
```

Checking whether linguistic similarity is a significant moderator.

```
capture.output(rma(yi,vi,mods=cbind(similar_binary,different_binary),data=data_op))[c(8,10,11,13,14)]
```

```
## [1] "R^2 (amount of heterogeneity accounted for):            0.00%"
## [2] "Test for Residual Heterogeneity: "
## [3] "QE(df = 89) = 682.3731, p-val < .0001"
## [4] "Test of Moderators (coefficient(s) 2): "
## [5] "QM(df = 1) = 0.0564, p-val = 0.8123"
```

Early subgroup - Overall Performance

```
summary(data_e_overall$ling_similar_2levels)
```

```
## different   similar
##        16        28
```

```
data_e_overall$similar_binary  <-ifelse(data_e_overall$ling_similar_2levels=="similar",1,0)
data_e_overall$different_binary<-ifelse(data_e_overall$ling_similar_2levels=="different",1,0)
```

```
print(m37<-rma(yi,vi,mods=cbind(similar_binary,different_binary),intercept=F,data=data_e_overall))
```

```
##
## Mixed-Effects Model (k = 44; tau^2 estimator: REML)
##
## tau^2 (estimated amount of residual heterogeneity):     0.0529 (SE = 0.0131)
## tau (square root of estimated tau^2 value):             0.2299
## I^2 (residual heterogeneity / unaccounted variability): 96.53%
## H^2 (unaccounted variability / sampling variability):   28.83
##
## Test for Residual Heterogeneity:
## QE(df = 42) = 436.4206, p-val < .0001
##
## Test of Moderators (coefficient(s) 1:2):
## QM(df = 2) = 2.6398, p-val = 0.2672
##
## Model Results:
##
##                   estimate      se     zval    pval    ci.lb   ci.ub
## similar_binary     -0.0457  0.0467  -0.9795  0.3273  -0.1372  0.0458
## different_binary    0.0797  0.0615   1.2963  0.1949  -0.0408  0.2002
##
## ---
## Signif. codes:  0 '***' 0.001 '**' 0.01 '*' 0.05 '.' 0.1 ' ' 1
```

```
#RR estimate  lower and upper boundaries of the 95% CI
round(exp(c(m37$b,m37$ci.lb,m37$ci.ub)),digits=2)
```

```
## [1] 0.96 1.08 0.87 0.96 1.05 1.22
```

Checking whether linguistic similarity is a significant moderator.

```
capture.output(rma(yi,vi,mods=cbind(similar_binary,different_binary),data=data_e_overall))[c(8,10,11,13
```

```
## [1] "R^2 (amount of heterogeneity accounted for):            3.30%"
## [2] "Test for Residual Heterogeneity: "
## [3] "QE(df = 42) = 436.4206, p-val < .0001"
## [4] "Test of Moderators (coefficient(s) 2): "
## [5] "QM(df = 1) = 2.6397, p-val = 0.1042"
```

Early subgroup - Auditory Comprehension

```
summary(data_e_ac$ling_similar_2levels)
```

```
## different   similar
##        15        23
```

```
data_e_ac$similar_binary  <-ifelse(data_e_ac$ling_similar_2levels=="similar",1,0)
data_e_ac$different_binary<-ifelse(data_e_ac$ling_similar_2levels=="different",1,0)
```

```
print(m38<-rma(yi,vi,mods=cbind(similar_binary,different_binary),intercept=F,data=data_e_ac))
```

```
##
## Mixed-Effects Model (k = 38; tau^2 estimator: REML)
##
## tau^2 (estimated amount of residual heterogeneity):     0.0225 (SE = 0.0071)
## tau (square root of estimated tau^2 value):             0.1500
## I^2 (residual heterogeneity / unaccounted variability): 87.31%
## H^2 (unaccounted variability / sampling variability):   7.88
##
## Test for Residual Heterogeneity:
## QE(df = 36) = 137.4949, p-val < .0001
##
## Test of Moderators (coefficient(s) 1:2):
## QM(df = 2) = 0.9448, p-val = 0.6235
##
## Model Results:
##
##                   estimate      se    zval    pval    ci.lb   ci.ub
## similar_binary      0.0224  0.0364  0.6153  0.5384  -0.0489  0.0936
## different_binary    0.0347  0.0462  0.7525  0.4518  -0.0558  0.1252
##
## ---
## Signif. codes:  0 '***' 0.001 '**' 0.01 '*' 0.05 '.' 0.1 ' ' 1
```

```
#RR estimate  lower and upper boundaries of the 95% CI
round(exp(c(m38$b,m38$ci.lb,m38$ci.ub)),digits=2)
```

```
## [1] 1.02 1.04 0.95 0.95 1.10 1.13
```

Checking whether linguistic similarity is a significant moderator.

```
capture.output(rma(yi,vi,mods=cbind(similar_binary,different_binary),data=data_e_ac))[c(8,10,11,13,14)]
```

```
## [1] "R^2 (amount of heterogeneity accounted for):            0.00%"
## [2] "Test for Residual Heterogeneity: "
## [3] "QE(df = 36) = 137.4949, p-val < .0001"
## [4] "Test of Moderators (coefficient(s) 2): "
## [5] "QM(df = 1) = 0.0444, p-val = 0.8331"
```

Early subgroup - Oral Production

```
summary(data_e_op$ling_similar_2levels)
```

```
## different   similar
##         8        24
```

```
data_e_op$similar_binary  <-ifelse(data_e_op$ling_similar_2levels=="similar",1,0)
data_e_op$different_binary<-ifelse(data_e_op$ling_similar_2levels=="different",1,0)
```

```
print(m39<-rma(yi,vi,mods=cbind(similar_binary,different_binary),intercept=F,data=data_e_op))
```

```
##
## Mixed-Effects Model (k = 32; tau^2 estimator: REML)
##
## tau^2 (estimated amount of residual heterogeneity):     0.0784 (SE = 0.0231)
## tau (square root of estimated tau^2 value):             0.2799
## I^2 (residual heterogeneity / unaccounted variability): 96.26%
## H^2 (unaccounted variability / sampling variability):   26.75
##
## Test for Residual Heterogeneity:
## QE(df = 30) = 281.0732, p-val < .0001
##
## Test of Moderators (coefficient(s) 1:2):
## QM(df = 2) = 1.9537, p-val = 0.3765
##
## Model Results:
##
##                     estimate      se     zval    pval    ci.lb   ci.ub
## similar_binary       -0.0670  0.0619  -1.0829  0.2788  -0.1883  0.0543
## different_binary      0.0917  0.1038   0.8837  0.3769  -0.1117  0.2952
##
## ---
## Signif. codes:  0 '***' 0.001 '**' 0.01 '*' 0.05 '.' 0.1 ' ' 1
```

```
#RR estimate  lower and upper boundaries of the 95% CI
round(exp(c(m39$b,m39$ci.lb,m39$ci.ub)),digits=2)
```

```
## [1] 0.94 1.10 0.83 0.89 1.06 1.34
```

Checking whether linguistic similarity is a significant moderator.

```
capture.output(rma(yi,vi,mods=cbind(similar_binary,different_binary),data=data_e_op))[c(8,10,11,13,14)]
```

```
## [1] "R^2 (amount of heterogeneity accounted for):            2.01%"
## [2] "Test for Residual Heterogeneity: "
## [3] "QE(df = 30) = 281.0732, p-val < .0001"
## [4] "Test of Moderators (coefficient(s) 2): "
## [5] "QM(df = 1) = 1.7254, p-val = 0.1890"
```

Late subgroup - Overall Performance

```
summary(data_l_overall$ling_similar_2levels)
```

```
## different   similar
##        13        62
```

```
data_l_overall$similar_binary  <-ifelse(data_l_overall$ling_similar_2levels=="similar",1,0)
data_l_overall$different_binary<-ifelse(data_l_overall$ling_similar_2levels=="different",1,0)
```

```
print(m40<-rma(yi,vi,mods=cbind(similar_binary,different_binary),intercept=F,data=data_l_overall))
```

```
##
## Mixed-Effects Model (k = 75; tau^2 estimator: REML)
##
## tau^2 (estimated amount of residual heterogeneity):     0.0409 (SE = 0.0083)
## tau (square root of estimated tau^2 value):             0.2021
## I^2 (residual heterogeneity / unaccounted variability): 90.72%
## H^2 (unaccounted variability / sampling variability):   10.77
##
## Test for Residual Heterogeneity:
## QE(df = 73) = 475.7721, p-val < .0001
##
## Test of Moderators (coefficient(s) 1:2):
## QM(df = 2) = 32.0092, p-val < .0001
##
## Model Results:
##
##                   estimate      se    zval    pval   ci.lb   ci.ub
## similar_binary      0.1464  0.0289  5.0682  <.0001  0.0898  0.2031  ***
## different_binary    0.1566  0.0623  2.5144  0.0119  0.0345  0.2787    *
##
## ---
## Signif. codes:  0 '***' 0.001 '**' 0.01 '*' 0.05 '.' 0.1 ' ' 1
```

```
#RR estimate  lower and upper boundaries of the 95% CI
round(exp(c(m40$b,m40$ci.lb,m40$ci.ub)),digits=2)
```

```
## [1] 1.16 1.17 1.09 1.04 1.23 1.32
```

Checking whether linguistic similarity is a significant moderator.

```
capture.output(rma(yi,vi,mods=cbind(similar_binary,different_binary),data=data_l_overall))[c(8,10,11,13
```

```
## [1] "R^2 (amount of heterogeneity accounted for):            0.00%"
## [2] "Test for Residual Heterogeneity: "
## [3] "QE(df = 73) = 475.7721, p-val < .0001"
## [4] "Test of Moderators (coefficient(s) 2): "
## [5] "QM(df = 1) = 0.0220, p-val = 0.8820"
```

Late subgroup - Auditory Comprehension

```
summary(data_l_ac$ling_similar_2levels)
```

```
## different   similar
##        10        43
```

```
data_l_ac$similar_binary  <-ifelse(data_l_ac$ling_similar_2levels=="similar",1,0)
data_l_ac$different_binary<-ifelse(data_l_ac$ling_similar_2levels=="different",1,0)
```

```
print(m41<-rma(yi,vi,mods=cbind(similar_binary,different_binary),intercept=F,data=data_l_ac))
```

```
##
## Mixed-Effects Model (k = 53; tau^2 estimator: REML)
##
## tau^2 (estimated amount of residual heterogeneity):     0.0226 (SE = 0.0065)
## tau (square root of estimated tau^2 value):             0.1504
## I^2 (residual heterogeneity / unaccounted variability): 84.75%
## H^2 (unaccounted variability / sampling variability):   6.56
##
## Test for Residual Heterogeneity:
## QE(df = 51) = 198.5825, p-val < .0001
##
## Test of Moderators (coefficient(s) 1:2):
## QM(df = 2) = 13.3606, p-val = 0.0013
##
## Model Results:
##
##                   estimate      se    zval    pval   ci.lb   ci.ub
## similar_binary      0.0718  0.0287  2.5029  0.0123  0.0156  0.1281   *
## different_binary    0.1482  0.0556  2.6639  0.0077  0.0391  0.2572  **
##
## ---
## Signif. codes:  0 '***' 0.001 '**' 0.01 '*' 0.05 '.' 0.1 ' ' 1
```

```
#RR estimate  lower and upper boundaries of the 95% CI
round(exp(c(m41$b,m41$ci.lb,m41$ci.ub)),digits=2)
```

```
## [1] 1.07 1.16 1.02 1.04 1.14 1.29
```

Checking whether linguistic similarity is a significant moderator.

```
capture.output(rma(yi,vi,mods=cbind(similar_binary,different_binary),data=data_l_ac))[c(8,10,11,13,14)]
```

```
## [1] "R^2 (amount of heterogeneity accounted for):            0.00%"
## [2] "Test for Residual Heterogeneity: "
## [3] "QE(df = 51) = 198.5825, p-val < .0001"
## [4] "Test of Moderators (coefficient(s) 2): "
## [5] "QM(df = 1) = 1.4880, p-val = 0.2225"
```

Late subgroup - Oral Production

```r
summary(data_l_op$ling_similar_2levels)
```

```
## different   similar
##        10        49
```

```r
data_l_op$similar_binary  <-ifelse(data_l_op$ling_similar_2levels=="similar",1,0)
data_l_op$different_binary<-ifelse(data_l_op$ling_similar_2levels=="different",1,0)
```

```r
print(m42<-rma(yi,vi,mods=cbind(similar_binary,different_binary),intercept=F,data=data_l_op))
```

```
##
## Mixed-Effects Model (k = 59; tau^2 estimator: REML)
##
## tau^2 (estimated amount of residual heterogeneity):     0.0586 (SE = 0.0137)
## tau (square root of estimated tau^2 value):             0.2421
## I^2 (residual heterogeneity / unaccounted variability): 87.00%
## H^2 (unaccounted variability / sampling variability):   7.69
##
## Test for Residual Heterogeneity:
## QE(df = 57) = 318.0590, p-val < .0001
##
## Test of Moderators (coefficient(s) 1:2):
## QM(df = 2) = 21.9186, p-val < .0001
##
## Model Results:
##
##                     estimate      se    zval    pval    ci.lb   ci.ub
## similar_binary        0.1812  0.0392  4.6207  <.0001   0.1043  0.2580  ***
## different_binary      0.0636  0.0844  0.7534  0.4512  -0.1019  0.2291
##
## ---
## Signif. codes:  0 '***' 0.001 '**' 0.01 '*' 0.05 '.' 0.1 ' ' 1
```

```r
round(exp(c(m42$b,m42$ci.lb,m42$ci.ub)),digits=2)
```

```
## [1] 1.20 1.07 1.11 0.90 1.29 1.26
```

Checking whether linguistic similarity is a significant moderator.

```r
capture.output(rma(yi,vi,mods=cbind(similar_binary,different_binary),
                   data=data_l_op))[c(8,10,11,13,14)]
```

```
## [1] "R^2 (amount of heterogeneity accounted for):            2.62%"
## [2] "Test for Residual Heterogeneity: "
## [3] "QE(df = 57) = 318.0590, p-val < .0001"
## [4] "Test of Moderators (coefficient(s) 2): "
## [5] "QM(df = 1) = 1.5954, p-val = 0.2065"
```

Does 3-level linguistic similarity moderate the difference betwen L1 and L2?

Whole group - Overall Performance

```
summary(data$ling_similar_3levels)
```

```
##      close  different very close
##         69         29         21
```

```
data$very_close<-ifelse(data$ling_similar_3levels=="very close",1,0)
data$different <-ifelse(data$ling_similar_3levels=="different",1,0)
data$close     <-ifelse(data$ling_similar_3levels=="close",1,0)
```

```
print(m34<-rma(yi,vi,mods=cbind(very_close,different,close),intercept=F,data=data))
```

```
##
## Mixed-Effects Model (k = 119; tau^2 estimator: REML)
##
## tau^2 (estimated amount of residual heterogeneity):     0.0527 (SE = 0.0081)
## tau (square root of estimated tau^2 value):             0.2296
## I^2 (residual heterogeneity / unaccounted variability): 94.84%
## H^2 (unaccounted variability / sampling variability):   19.37
##
## Test for Residual Heterogeneity:
## QE(df = 116) = 1012.9154, p-val < .0001
##
## Test of Moderators (coefficient(s) 1:3):
## QM(df = 3) = 17.2421, p-val = 0.0006
##
## Model Results:
##
##             estimate      se    zval    pval    ci.lb   ci.ub
## very_close    0.0542  0.0546  0.9922  0.3211  -0.0528  0.1611
## different     0.1169  0.0460  2.5396  0.0111   0.0267  0.2071   *
## close         0.0948  0.0303  3.1318  0.0017   0.0355  0.1541  **
##
## ---
## Signif. codes:  0 '***' 0.001 '**' 0.01 '*' 0.05 '.' 0.1 ' ' 1
```

```
#RR estimate  lower and upper boundaries of the 95% CI
round(exp(c(m34$b,m34$ci.lb,m34$ci.ub)),digits=2)
```

```
## [1] 1.06 1.12 1.10 0.95 1.03 1.04 1.17 1.23 1.17
```

Checking whether linguistic similarity is a significant moderator.

```
capture.output(rma(yi,vi,mods=cbind(very_close,different,close),data=data))[c(8,10,11,13,14)]
```

```
## [1] "R^2 (amount of heterogeneity accounted for):            0.00%"
## [2] "Test for Residual Heterogeneity: "
## [3] "QE(df = 116) = 1012.9154, p-val < .0001"
## [4] "Test of Moderators (coefficient(s) 2:3): "
## [5] "QM(df = 2) = 0.7790, p-val = 0.6774"
```

Whole group - Auditory Comprehension

```
summary(data_ac$ling_similar_3levels)
```

```
##      close  different very close
##         49         25         17
```

```
data_ac$very_close<-ifelse(data_ac$ling_similar_3levels=="similar",1,0)
data_ac$different <-ifelse(data_ac$ling_similar_3levels=="different",1,0)
data_ac$close     <-ifelse(data_ac$ling_similar_3levels=="close",1,0)
```

```
print(m35<-rma(yi,vi,mods=cbind(very_close,different,close),intercept=F,data=data_ac))
```

```
##
## Mixed-Effects Model (k = 91; tau^2 estimator: REML)
##
## tau^2 (estimated amount of residual heterogeneity):     0.0240 (SE = 0.0049)
## tau (square root of estimated tau^2 value):             0.1550
## I^2 (residual heterogeneity / unaccounted variability): 87.22%
## H^2 (unaccounted variability / sampling variability):   7.83
##
## Test for Residual Heterogeneity:
## QE(df = 89) = 372.2566, p-val < .0001
##
## Test of Moderators (coefficient(s) 1:2):
## QM(df = 2) = 8.5055, p-val = 0.0142
##
## Model Results:
##
##            estimate      se    zval    pval    ci.lb   ci.ub
## different    0.0819  0.0364  2.2499  0.0245   0.0106  0.1533  *
## close        0.0497  0.0268  1.8557  0.0635  -0.0028  0.1022  .
##
## ---
## Signif. codes:  0 '***' 0.001 '**' 0.01 '*' 0.05 '.' 0.1 ' ' 1
```

```
#RR estimate  lower and upper boundaries of the 95% CI
round(exp(c(m35$b,m35$ci.lb,m35$ci.ub)),digits=2)
```

```
## [1] 1.09 1.05 1.01 1.00 1.17 1.11
```

Checking whether linguistic similarity is a significant moderator.

```
capture.output(rma(yi,vi,mods=cbind(very_close,different,close),data=data_ac))[c(8,10,11,13,14)]
```

```
## [1] "R^2 (amount of heterogeneity accounted for):            0.00%"
## [2] "Test for Residual Heterogeneity: "
## [3] "QE(df = 88) = 359.2996, p-val < .0001"
## [4] "Test of Moderators (coefficient(s) 2:3): "
## [5] "QM(df = 2) = 0.5239, p-val = 0.7696"
```

Whole group - Oral Production

```
summary(data_op$ling_similar_3levels)
```

```
##      close  different very close
##         57         18         16
```

```
data_op$very_close<-ifelse(data_op$ling_similar_3levels=="similar",1,0)
data_op$different <-ifelse(data_op$ling_similar_3levels=="different",1,0)
data_op$close     <-ifelse(data_op$ling_similar_3levels=="close",1,0)
```

```
print(m36<-rma(yi,vi,mods=cbind(very_close,different,close ),intercept=F,data=data_op))
```

```
##
## Mixed-Effects Model (k = 91; tau^2 estimator: REML)
##
## tau^2 (estimated amount of residual heterogeneity):     0.0784 (SE = 0.0138)
## tau (square root of estimated tau^2 value):             0.2800
## I^2 (residual heterogeneity / unaccounted variability): 93.81%
## H^2 (unaccounted variability / sampling variability):   16.16
##
## Test for Residual Heterogeneity:
## QE(df = 89) = 691.4279, p-val < .0001
##
## Test of Moderators (coefficient(s) 1:2):
## QM(df = 2) = 7.4715, p-val = 0.0239
##
## Model Results:
##
##            estimate      se    zval    pval    ci.lb   ci.ub
## different    0.0773  0.0703  1.0991  0.2717  -0.0605  0.2152
## close        0.1024  0.0409  2.5027  0.0123   0.0222  0.1827  *
##
## ---
## Signif. codes:  0 '***' 0.001 '**' 0.01 '*' 0.05 '.' 0.1 ' ' 1
```

```
#RR estimate  lower and upper boundaries of the 95% CI
round(exp(c(m36$b,m36$ci.lb,m36$ci.ub)),digits=2)
```

```
## [1] 1.08 1.11 0.94 1.02 1.24 1.20
```

Checking whether linguistic similarity is a significant moderator.

```
capture.output(rma(yi,vi,mods=cbind(very_close,different,close),data=data_op))[c(8,10,11,13,14)]
```

```
## [1] "R^2 (amount of heterogeneity accounted for):            0.00%"
## [2] "Test for Residual Heterogeneity: "
## [3] "QE(df = 88) = 681.7984, p-val < .0001"
## [4] "Test of Moderators (coefficient(s) 2:3): "
## [5] "QM(df = 2) = 0.1660, p-val = 0.9204"
```

Early subgroup - Overall Performance

```
summary(data_e_overall$ling_similar_3levels)
```

```
##      close  different very close
##         21         16          7
```

```
data_e_overall$very_close<-ifelse(data_e_overall$ling_similar_3levels=="similar",1,0)
data_e_overall$different <-ifelse(data_e_overall$ling_similar_3levels=="different",1,0)
data_e_overall$closet_new<-ifelse(data_e_overall$ling_similar_3levels=="close",1,0)
```

```
print(m37<-rma(yi,vi,mods=cbind(very_close,different,closet_new),intercept=F,data=data_e_overall))
```

```
##
## Mixed-Effects Model (k = 44; tau^2 estimator: REML)
##
## tau^2 (estimated amount of residual heterogeneity):     0.0530 (SE = 0.0131)
## tau (square root of estimated tau^2 value):             0.2302
## I^2 (residual heterogeneity / unaccounted variability): 96.53%
## H^2 (unaccounted variability / sampling variability):   28.81
##
## Test for Residual Heterogeneity:
## QE(df = 42) = 435.0546, p-val < .0001
##
## Test of Moderators (coefficient(s) 1:2):
## QM(df = 2) = 2.9197, p-val = 0.2323
##
## Model Results:
##
##              estimate      se     zval    pval    ci.lb   ci.ub
## different      0.0797  0.0615   1.2954  0.1952  -0.0409  0.2003
## closet_new    -0.0591  0.0531  -1.1142  0.2652  -0.1631  0.0449
##
## ---
## Signif. codes:  0 '***' 0.001 '**' 0.01 '*' 0.05 '.' 0.1 ' ' 1
```

```
#RR estimate  lower and upper boundaries of the 95% CI
round(exp(c(m37$b,m37$ci.lb,m37$ci.ub)),digits=2)
```

```
## [1] 1.08 0.94 0.96 0.85 1.22 1.05
```

Checking whether linguistic similarity is a significant moderator.

```
capture.output(rma(yi,vi,mods=cbind(very_close,different,closet_new),data=data_e_overall))[c(8,10,11,13
```

```
## [1] "R^2 (amount of heterogeneity accounted for):            0.07%"
## [2] "Test for Residual Heterogeneity: "
## [3] "QE(df = 41) = 434.4402, p-val < .0001"
## [4] "Test of Moderators (coefficient(s) 2:3): "
## [5] "QM(df = 2) = 2.8655, p-val = 0.2387"
```

Early subgroup - Auditory Comprehension

```
summary(data_e_ac$ling_similar_3levels)
```

```
##      close  different very close
##         19         15          4
```

```
data_e_ac$very_close  <-ifelse(data_e_ac$ling_similar_3levels=="similar",1,0)
data_e_ac$different<-ifelse(data_e_ac$ling_similar_3levels=="different",1,0)
data_e_ac$close<-ifelse(data_e_ac$ling_similar_3levels=="close",1,0)
```

```
print(m38<-rma(yi,vi,mods=cbind(very_close,different,close),intercept=F,data=data_e_ac))
```

```
##
## Mixed-Effects Model (k = 38; tau^2 estimator: REML)
##
## tau^2 (estimated amount of residual heterogeneity):     0.0226 (SE = 0.0071)
## tau (square root of estimated tau^2 value):             0.1504
## I^2 (residual heterogeneity / unaccounted variability): 87.33%
## H^2 (unaccounted variability / sampling variability):   7.89
##
## Test for Residual Heterogeneity:
## QE(df = 36) = 136.6047, p-val < .0001
##
## Test of Moderators (coefficient(s) 1:2):
## QM(df = 2) = 0.8267, p-val = 0.6614
##
## Model Results:
##
##            estimate      se    zval    pval    ci.lb   ci.ub
## different    0.0348  0.0463  0.7514  0.4524  -0.0559  0.1254
## close        0.0206  0.0402  0.5120  0.6087  -0.0582  0.0993
##
## ---
## Signif. codes:  0 '***' 0.001 '**' 0.01 '*' 0.05 '.' 0.1 ' ' 1
```

```
#RR estimate  lower and upper boundaries of the 95% CI
round(exp(c(m38$b,m38$ci.lb,m38$ci.ub)),digits=2)
```

```
## [1] 1.04 1.02 0.95 0.94 1.13 1.10
```

Checking whether linguistic similarity is a significant moderator.

```
capture.output(rma(yi,vi,mods=cbind(very_close,different,close),data=data_e_ac))[c(8,10,11,13,14)]
```

```
## [1] "R^2 (amount of heterogeneity accounted for):            0.00%"
## [2] "Test for Residual Heterogeneity: "
## [3] "QE(df = 35) = 136.4694, p-val < .0001"
## [4] "Test of Moderators (coefficient(s) 2:3): "
## [5] "QM(df = 2) = 0.0475, p-val = 0.9765"
```

Early subgroup - Oral Production

```
summary(data_e_op$ling_similar_3levels)
```

```
##      close  different very close
##         17          8          7
```

```
data_e_op$very_close  <-ifelse(data_e_op$ling_similar_3levels=="similar",1,0)
data_e_op$different<-ifelse(data_e_op$ling_similar_3levels=="different",1,0)
data_e_op$close<-ifelse(data_e_op$ling_similar_3levels=="close",1,0)
```

```
print(m39<-rma(yi,vi,mods=cbind(very_close,different,close),intercept=F,data=data_e_op))
```

```
##
## Mixed-Effects Model (k = 32; tau^2 estimator: REML)
##
## tau^2 (estimated amount of residual heterogeneity):     0.0786 (SE = 0.0232)
## tau (square root of estimated tau^2 value):             0.2804
## I^2 (residual heterogeneity / unaccounted variability): 96.24%
## H^2 (unaccounted variability / sampling variability):   26.58
##
## Test for Residual Heterogeneity:
## QE(df = 30) = 280.7257, p-val < .0001
##
## Test of Moderators (coefficient(s) 1:2):
## QM(df = 2) = 2.2670, p-val = 0.3219
##
## Model Results:
##
##            estimate      se     zval    pval    ci.lb   ci.ub
## different    0.0918  0.1040   0.8828  0.3773  -0.1120  0.2956
## close       -0.0883  0.0724  -1.2197  0.2226  -0.2303  0.0536
##
## ---
## Signif. codes:  0 '***' 0.001 '**' 0.01 '*' 0.05 '.' 0.1 ' ' 1
```

```
#RR estimate  lower and upper boundaries of the 95% CI
round(exp(c(m39$b,m39$ci.lb,m39$ci.ub)),digits=2)
```

```
## [1] 1.10 0.92 0.89 0.79 1.34 1.06
```

Checking whether linguistic similarity is a significant moderator.

```
capture.output(rma(yi,vi,mods=cbind(very_close,different,close),data=data_e_op))[c(8,10,11,13,14)]
```

```
## [1] "R^2 (amount of heterogeneity accounted for):            0.00%"
## [2] "Test for Residual Heterogeneity: "
## [3] "QE(df = 29) = 280.5239, p-val < .0001"
## [4] "Test of Moderators (coefficient(s) 2:3): "
## [5] "QM(df = 2) = 1.9903, p-val = 0.3697"
```

Late subgroup - Overall Performance

```
summary(data_l_overall$ling_similar_3levels)
```

```
##      close  different very close
##         48         13         14
```

```
data_l_overall$very_close  <-ifelse(data_l_overall$ling_similar_3levels=="similar",1,0)
data_l_overall$different<-ifelse(data_l_overall$ling_similar_3levels=="different",1,0)
data_l_overall$close<-ifelse(data_l_overall$ling_similar_3levels=="close",1,0)
```

```
print(m40<-rma(yi,vi,mods=cbind(very_close,different,close),intercept=F,data=data_l_overall))
```

```
##
## Mixed-Effects Model (k = 75; tau^2 estimator: REML)
##
## tau^2 (estimated amount of residual heterogeneity):     0.0402 (SE = 0.0082)
## tau (square root of estimated tau^2 value):             0.2006
## I^2 (residual heterogeneity / unaccounted variability): 90.60%
## H^2 (unaccounted variability / sampling variability):   10.64
##
## Test for Residual Heterogeneity:
## QE(df = 73) = 530.5710, p-val < .0001
##
## Test of Moderators (coefficient(s) 1:2):
## QM(df = 2) = 32.5524, p-val < .0001
##
## Model Results:
##
##            estimate      se    zval    pval   ci.lb   ci.ub
## different    0.1561  0.0619  2.5230  0.0116  0.0348  0.2775    *
## close        0.1690  0.0330  5.1173  <.0001  0.1043  0.2337  ***
##
## ---
## Signif. codes:  0 '***' 0.001 '**' 0.01 '*' 0.05 '.' 0.1 ' ' 1
```

```
#RR estimate  lower and upper boundaries of the 95% CI
round(exp(c(m40$b,m40$ci.lb,m40$ci.ub)),digits=2)
```

```
## [1] 1.17 1.18 1.04 1.11 1.32 1.26
```

Checking whether linguistic similarity is a significant moderator.

```
capture.output(rma(yi,vi,mods=cbind(very_close,different,close),data=data_l_overall))[c(8,10,11,13,14)]
```

```
## [1] "R^2 (amount of heterogeneity accounted for):            0.01%"
## [2] "Test for Residual Heterogeneity: "
## [3] "QE(df = 72) = 472.0054, p-val < .0001"
## [4] "Test of Moderators (coefficient(s) 2:3): "
## [5] "QM(df = 2) = 1.9592, p-val = 0.3755"
```

Late subgroup - Auditory Comprehension

```
summary(data_l_ac$ling_similar_3levels)
```

```
##      close  different very close
##         30         10         13
```

```
data_l_ac$very_close  <-ifelse(data_l_ac$ling_similar_3levels=="similar",1,0)
data_l_ac$different<-ifelse(data_l_ac$ling_similar_3levels=="different",1,0)
data_l_ac$close<-ifelse(data_l_ac$ling_similar_3levels=="close",1,0)
```

```
print(m41<-rma(yi,vi,mods=cbind(very_close,different,close),intercept=F,data=data_l_ac))
```

```
##
## Mixed-Effects Model (k = 53; tau^2 estimator: REML)
##
## tau^2 (estimated amount of residual heterogeneity):     0.0248 (SE = 0.0070)
## tau (square root of estimated tau^2 value):             0.1574
## I^2 (residual heterogeneity / unaccounted variability): 85.40%
## H^2 (unaccounted variability / sampling variability):   6.85
##
## Test for Residual Heterogeneity:
## QE(df = 51) = 213.7761, p-val < .0001
##
## Test of Moderators (coefficient(s) 1:2):
## QM(df = 2) = 10.7670, p-val = 0.0046
##
## Model Results:
##
##            estimate      se    zval    pval   ci.lb   ci.ub
## different    0.1507  0.0577  2.6142  0.0089  0.0377  0.2637  **
## close        0.0708  0.0357  1.9832  0.0473  0.0008  0.1408   *
##
## ---
## Signif. codes:  0 '***' 0.001 '**' 0.01 '*' 0.05 '.' 0.1 ' ' 1
```

```
#RR estimate  lower and upper boundaries of the 95% CI
round(exp(c(m41$b,m41$ci.lb,m41$ci.ub)),digits=2)
```

```
## [1] 1.16 1.07 1.04 1.00 1.30 1.15
```

Checking whether linguistic similarity is a significant moderator.

```
capture.output(rma(yi,vi,mods=cbind(very_close,different,close),data=data_l_ac))[c(8,10,11,13,14)]
```

```
## [1] "R^2 (amount of heterogeneity accounted for):            0.00%"
## [2] "Test for Residual Heterogeneity: "
## [3] "QE(df = 50) = 197.3278, p-val < .0001"
## [4] "Test of Moderators (coefficient(s) 2:3): "
## [5] "QM(df = 2) = 1.4850, p-val = 0.4759"
```

Late subgroup - Oral Production

```
summary(data_l_op$ling_similar_3levels)
```

```
##      close  different very close
##         40         10          9
```

```
data_l_op$very_close  <-ifelse(data_l_op$ling_similar_3levels=="similar",1,0)
data_l_op$different<-ifelse(data_l_op$ling_similar_3levels=="different",1,0)
data_l_op$close<-ifelse(data_l_op$ling_similar_3levels=="close",1,0)
```

```
print(m42<-rma(yi,vi,mods=cbind(very_close,different,close),intercept=F,
                 data=data_l_op))
```

```
##
## Mixed-Effects Model (k = 59; tau^2 estimator: REML)
##
## tau^2 (estimated amount of residual heterogeneity):     0.0611 (SE = 0.0142)
## tau (square root of estimated tau^2 value):             0.2471
## I^2 (residual heterogeneity / unaccounted variability): 87.52%
## H^2 (unaccounted variability / sampling variability):   8.02
##
## Test for Residual Heterogeneity:
## QE(df = 57) = 322.8733, p-val < .0001
##
## Test of Moderators (coefficient(s) 1:2):
## QM(df = 2) = 19.4494, p-val < .0001
##
## Model Results:
##
##            estimate      se    zval    pval    ci.lb   ci.ub
## different    0.0638  0.0859  0.7430  0.4575  -0.1045  0.2322
## close        0.1951  0.0449  4.3471  <.0001   0.1071  0.2830  ***
##
## ---
## Signif. codes:  0 '***' 0.001 '**' 0.01 '*' 0.05 '.' 0.1 ' ' 1
```

```
round(exp(c(m42$b,m42$ci.lb,m42$ci.ub)),digits=2)
```

```
## [1] 1.07 1.22 0.90 1.11 1.26 1.33
```

Checking whether linguistic similarity is a significant moderator.

```
capture.output(rma(yi,vi,mods=cbind(very_close,different,close),data=data_l_op))[c(8,10,11,13,14)]
```

```
## [1] "R^2 (amount of heterogeneity accounted for):            1.84%"
## [2] "Test for Residual Heterogeneity: "
## [3] "QE(df = 56) = 305.0587, p-val < .0001"
## [4] "Test of Moderators (coefficient(s) 2:3): "
## [5] "QM(df = 2) = 2.0772, p-val = 0.3539"
```

Additional Variables

Does age at the time of assessment moderate the difference betwen L1 and L2?

Whole group - Overall Performance

```
summary(data$case_age)
```

```
##    Min. 1st Qu.  Median    Mean 3rd Qu.    Max.
##    17.0    47.7    59.0    58.5    69.0    91.0
```

```
print(mpo_all_overall<-rma(yi,vi,mods=case_age,data=data))
```

```
##
## Mixed-Effects Model (k = 119; tau^2 estimator: REML)
##
## tau^2 (estimated amount of residual heterogeneity):     0.0476 (SE = 0.0074)
## tau (square root of estimated tau^2 value):             0.2182
## I^2 (residual heterogeneity / unaccounted variability): 94.39%
## H^2 (unaccounted variability / sampling variability):   17.83
## R^2 (amount of heterogeneity accounted for):            7.97%
##
## Test for Residual Heterogeneity:
## QE(df = 117) = 991.8132, p-val < .0001
##
## Test of Moderators (coefficient(s) 2):
## QM(df = 1) = 8.7099, p-val = 0.0032
##
## Model Results:
##
##          estimate      se     zval    pval    ci.lb   ci.ub
## intrcpt   -0.1805  0.0950  -1.9003  0.0574  -0.3667  0.0057   .
## mods       0.0047  0.0016   2.9513  0.0032   0.0016  0.0078  **
##
## ---
## Signif. codes:  0 '***' 0.001 '**' 0.01 '*' 0.05 '.' 0.1 ' ' 1
```

```
#RR estimate,lower and upper boundaries of the 95% CI
round(exp(c(mpo_all_overall$b,mpo_all_overall$ci.lb,mpo_all_overall$ci.ub)),digits=2)
```

```
## [1] 0.83 1.00 0.69 1.00 1.01 1.01
```

Whole group - Auditory Comprehension

```
summary(data_ac$case_age)
```

```
##    Min. 1st Qu.  Median    Mean 3rd Qu.    Max.
##   17.00   46.50   55.00   56.54   65.00   91.00
```

```r
print(mpo_all_ac<-rma(yi,vi,mods=case_age,data=data_ac))
```

```
##
## Mixed-Effects Model (k = 91; tau^2 estimator: REML)
##
## tau^2 (estimated amount of residual heterogeneity):     0.0203 (SE = 0.0043)
## tau (square root of estimated tau^2 value):             0.1426
## I^2 (residual heterogeneity / unaccounted variability): 85.60%
## H^2 (unaccounted variability / sampling variability):   6.95
## R^2 (amount of heterogeneity accounted for):            9.74%
##
## Test for Residual Heterogeneity:
## QE(df = 89) = 353.7820, p-val < .0001
##
## Test of Moderators (coefficient(s) 2):
## QM(df = 1) = 5.6965, p-val = 0.0170
##
## Model Results:
##
##          estimate      se     zval    pval    ci.lb   ci.ub
## intrcpt   -0.1186  0.0770  -1.5404  0.1235  -0.2695  0.0323
## mods       0.0032  0.0013   2.3867  0.0170   0.0006  0.0058  *
##
## ---
## Signif. codes:  0 '***' 0.001 '**' 0.01 '*' 0.05 '.' 0.1 ' ' 1
```

```r
#RR estimate,lower and upper boundaries of the 95% CI
round(exp(c(mpo_all_ac$b,mpo_all_ac$ci.lb,mpo_all_ac$ci.ub)),digits=2)
```

```
## [1] 0.89 1.00 0.76 1.00 1.03 1.01
```

Whole group - Oral Production

```r
summary(data_op$case_age)
```

```
##    Min. 1st Qu.  Median    Mean 3rd Qu.    Max.
##   17.00   49.00   59.00   58.78   69.50   91.00
```

```r
print(mpo_all_op<-rma(yi,vi,mods=case_age,data=data_op))
```

```
##
## Mixed-Effects Model (k = 91; tau^2 estimator: REML)
##
## tau^2 (estimated amount of residual heterogeneity):     0.0736 (SE = 0.0131)
## tau (square root of estimated tau^2 value):             0.2712
## I^2 (residual heterogeneity / unaccounted variability): 93.45%
## H^2 (unaccounted variability / sampling variability):   15.26
## R^2 (amount of heterogeneity accounted for):            4.16%
##
## Test for Residual Heterogeneity:
```

```
## QE(df = 89) = 671.0586, p-val < .0001
##
## Test of Moderators (coefficient(s) 2):
## QM(df = 1) = 3.7151, p-val = 0.0539
##
## Model Results:
##
##          estimate      se     zval    pval    ci.lb   ci.ub
## intrcpt   -0.1685  0.1387  -1.2144  0.2246  -0.4404  0.1034
## mods       0.0044  0.0023   1.9275  0.0539  -0.0001  0.0090  .
##
## ---
## Signif. codes:  0 '***' 0.001 '**' 0.01 '*' 0.05 '.' 0.1 ' ' 1
```

```r
#RR estimate,lower and upper boundaries of the 95% CI
round(exp(c(mpo_all_op$b,mpo_all_op$ci.lb,mpo_all_op$ci.ub)),digits=2)
```

```
## [1] 0.84 1.00 0.64 1.00 1.11 1.01
```

Early subgroup - Overearly Performance

```r
summary(data_e_overall$case_age)
```

```
##    Min. 1st Qu.  Median    Mean 3rd Qu.    Max.
##   17.00   43.00   53.50   52.87   62.25   84.00
```

```r
print(mpo_early_overall<-rma(yi,vi,mods=case_age,data=data_e_overall))
```

```
##
## Mixed-Effects Model (k = 44; tau^2 estimator: REML)
##
## tau^2 (estimated amount of residual heterogeneity):     0.0554 (SE = 0.0137)
## tau (square root of estimated tau^2 value):             0.2354
## I^2 (residual heterogeneity / unaccounted variability): 96.70%
## H^2 (unaccounted variability / sampling variability):   30.33
## R^2 (amount of heterogeneity accounted for):            0.00%
##
## Test for Residual Heterogeneity:
## QE(df = 42) = 436.3241, p-val < .0001
##
## Test of Moderators (coefficient(s) 2):
## QM(df = 1) = 1.2032, p-val = 0.2727
##
## Model Results:
##
##          estimate      se     zval    pval    ci.lb   ci.ub
## intrcpt   -0.1591  0.1503  -1.0588  0.2897  -0.4537  0.1354
## mods       0.0030  0.0028   1.0969  0.2727  -0.0024  0.0085
##
## ---
## Signif. codes:  0 '***' 0.001 '**' 0.01 '*' 0.05 '.' 0.1 ' ' 1
```

```r
#RR estimate,lower and upper boundaries of the 95% CI
round(exp(c(mpo_early_overall$b,mpo_early_overall$ci.lb,mpo_early_overall$ci.ub)),digits=2)
```

```
## [1] 0.85 1.00 0.64 1.00 1.15 1.01
```

Early subgroup - Auditory Comprehension

```r
summary(data_e_ac$case_age)
```

```
##    Min. 1st Qu.  Median    Mean 3rd Qu.    Max.
##   17.00   43.25   53.00   51.96   61.50   80.00
```

```r
print(mpo_early_ac<-rma(yi,vi,mods=case_age,data=data_e_ac))
```

```
##
## Mixed-Effects Model (k = 38; tau^2 estimator: REML)
##
## tau^2 (estimated amount of residual heterogeneity):     0.0221 (SE = 0.0070)
## tau (square root of estimated tau^2 value):             0.1485
## I^2 (residual heterogeneity / unaccounted variability): 87.33%
## H^2 (unaccounted variability / sampling variability):   7.89
## R^2 (amount of heterogeneity accounted for):            0.00%
##
## Test for Residual Heterogeneity:
## QE(df = 36) = 141.2390, p-val < .0001
##
## Test of Moderators (coefficient(s) 2):
## QM(df = 1) = 0.9793, p-val = 0.3224
##
## Model Results:
##
##          estimate      se     zval    pval    ci.lb   ci.ub
## intrcpt   -0.0798  0.1115  -0.7158  0.4741  -0.2984  0.1388
## mods       0.0021  0.0021   0.9896  0.3224  -0.0020  0.0062
##
## ---
## Signif. codes:  0 '***' 0.001 '**' 0.01 '*' 0.05 '.' 0.1 ' ' 1
```

```r
#RR estimate,lower and upper boundaries of the 95% CI
round(exp(c(mpo_early_ac$b,mpo_early_ac$ci.lb,mpo_early_ac$ci.ub)),digits=2)
```

```
## [1] 0.92 1.00 0.74 1.00 1.15 1.01
```

Early subgroup - Oral Production

```r
summary(data_e_op$case_age)
```

```
##    Min. 1st Qu.  Median    Mean 3rd Qu.    Max.
##   17.00   43.75   53.50   54.33   65.25   84.00
```

```r
print(mpo_early_op<-rma(yi,vi,mods=case_age,data=data_e_op))
```

```
##
## Mixed-Effects Model (k = 32; tau^2 estimator: REML)
##
## tau^2 (estimated amount of residual heterogeneity):     0.0838 (SE = 0.0246)
## tau (square root of estimated tau^2 value):             0.2895
## I^2 (residual heterogeneity / unaccounted variability): 96.49%
## H^2 (unaccounted variability / sampling variability):   28.47
## R^2 (amount of heterogeneity accounted for):            0.00%
##
## Test for Residual Heterogeneity:
## QE(df = 30) = 281.4171, p-val < .0001
##
## Test of Moderators (coefficient(s) 2):
## QM(df = 1) = 0.0910, p-val = 0.7629
##
## Model Results:
##
##          estimate      se     zval    pval    ci.lb   ci.ub
## intrcpt   -0.0863  0.2096  -0.4116  0.6806  -0.4970  0.3245
## mods       0.0011  0.0037   0.3017  0.7629  -0.0062  0.0085
##
## ---
## Signif. codes:  0 '***' 0.001 '**' 0.01 '*' 0.05 '.' 0.1 ' ' 1
```

```r
#RR estimate,lower and upper boundaries of the 95% CI
round(exp(c(mpo_early_op$b,mpo_early_op$ci.lb,mpo_early_op$ci.ub)),digits=2)
```

```
## [1] 0.92 1.00 0.61 0.99 1.38 1.01
```

Late subgroup - Overlate Performance

```r
summary(data_l_overall$case_age)
```

```
##    Min. 1st Qu.  Median    Mean 3rd Qu.    Max.
##    33.0    52.0    63.0    61.8    72.0    91.0
```

```r
print(mpo_late_overall<-rma(yi,vi,mods=case_age,data=data_l_overall))
```

```
##
## Mixed-Effects Model (k = 75; tau^2 estimator: REML)
##
## tau^2 (estimated amount of residual heterogeneity):     0.0376 (SE = 0.0078)
## tau (square root of estimated tau^2 value):             0.1938
## I^2 (residual heterogeneity / unaccounted variability): 89.80%
## H^2 (unaccounted variability / sampling variability):   9.80
## R^2 (amount of heterogeneity accounted for):            5.62%
##
```

```
## Test for Residual Heterogeneity:
## QE(df = 73) = 464.9453, p-val < .0001
##
## Test of Moderators (coefficient(s) 2):
## QM(df = 1) = 3.3600, p-val = 0.0668
##
## Model Results:
##
##          estimate      se     zval    pval    ci.lb   ci.ub
## intrcpt   -0.0785  0.1258  -0.6241  0.5325  -0.3252  0.1681
## mods       0.0037  0.0020   1.8330  0.0668  -0.0003  0.0076  .
##
## ---
## Signif. codes:  0 '***' 0.001 '**' 0.01 '*' 0.05 '.' 0.1 ' ' 1
```

```r
#RR estimate,lower and upper boundaries of the 95% CI
round(exp(c(mpo_late_overall$b,mpo_late_overall$ci.lb,mpo_late_overall$ci.ub)),digits=2)
```

```
## [1] 0.92 1.00 0.72 1.00 1.18 1.01
```

Late subgroup - Auditory Comprehension

```r
summary(data_l_ac$case_age)
```

```
##    Min. 1st Qu.  Median    Mean 3rd Qu.    Max.
##   33.00   50.00   57.00   59.83   70.00   91.00
```

```r
print(mpo_late_ac<-rma(yi,vi,mods=case_age,data=data_l_ac))
```

```
##
## Mixed-Effects Model (k = 53; tau^2 estimator: REML)
##
## tau^2 (estimated amount of residual heterogeneity):     0.0194 (SE = 0.0057)
## tau (square root of estimated tau^2 value):             0.1392
## I^2 (residual heterogeneity / unaccounted variability): 83.10%
## H^2 (unaccounted variability / sampling variability):   5.92
## R^2 (amount of heterogeneity accounted for):            11.88%
##
## Test for Residual Heterogeneity:
## QE(df = 51) = 191.4492, p-val < .0001
##
## Test of Moderators (coefficient(s) 2):
## QM(df = 1) = 3.3790, p-val = 0.0660
##
## Model Results:
##
##          estimate      se     zval    pval    ci.lb   ci.ub
## intrcpt   -0.1208  0.1152  -1.0480  0.2946  -0.3466  0.1051
## mods       0.0035  0.0019   1.8382  0.0660  -0.0002  0.0072  .
##
## ---
## Signif. codes:  0 '***' 0.001 '**' 0.01 '*' 0.05 '.' 0.1 ' ' 1
```

```r
#RR estimate,lower and upper boundaries of the 95% CI
round(exp(c(mpo_late_ac$b,mpo_late_ac$ci.lb,mpo_late_ac$ci.ub)),digits=2)
```

```
## [1] 0.89 1.00 0.71 1.00 1.11 1.01
```

Late subgroup - Oral Production

```r
summary(data_l_op$case_age)
```

```
##    Min. 1st Qu.  Median    Mean 3rd Qu.    Max.
##    40.0    52.0    60.0    61.2    71.0    91.0
```

```r
print(mpo_late_op<-rma(yi,vi,mods=case_age,data=data_l_op))
```

```
##
## Mixed-Effects Model (k = 59; tau^2 estimator: REML)
##
## tau^2 (estimated amount of residual heterogeneity):     0.0576 (SE = 0.0136)
## tau (square root of estimated tau^2 value):             0.2401
## I^2 (residual heterogeneity / unaccounted variability): 86.58%
## H^2 (unaccounted variability / sampling variability):   7.45
## R^2 (amount of heterogeneity accounted for):            4.21%
##
## Test for Residual Heterogeneity:
## QE(df = 57) = 323.9185, p-val < .0001
##
## Test of Moderators (coefficient(s) 2):
## QM(df = 1) = 2.3992, p-val = 0.1214
##
## Model Results:
##
##          estimate      se     zval    pval    ci.lb   ci.ub
## intrcpt   -0.1178  0.1830  -0.6434  0.5200  -0.4765  0.2409
## mods       0.0045  0.0029   1.5489  0.1214  -0.0012  0.0103
##
## ---
## Signif. codes:  0 '***' 0.001 '**' 0.01 '*' 0.05 '.' 0.1 ' ' 1
```

```r
#RR estimate,lower and upper boundaries of the 95% CI
round(exp(c(mpo_late_op$b,mpo_late_op$ci.lb,mpo_late_op$ci.ub)),digits=2)
```

```
## [1] 0.89 1.00 0.62 1.00 1.27 1.01
```

Do years of education moderate the difference betwen L1 and L2?

Whole group - Overall Performance

```r
summary(data$case_scholarity_years)
```

```
##    Min. 1st Qu.  Median    Mean 3rd Qu.    Max.    NA's
##    1.00    9.50   12.00   12.24   16.00   22.00      48
```

```r
print(education_all_overall<-rma(yi,vi,mods=case_scholarity_years,data=data))
```

```
##
## Mixed-Effects Model (k = 71; tau^2 estimator: REML)
##
## tau^2 (estimated amount of residual heterogeneity):     0.0426 (SE = 0.0085)
## tau (square root of estimated tau^2 value):             0.2064
## I^2 (residual heterogeneity / unaccounted variability): 93.70%
## H^2 (unaccounted variability / sampling variability):   15.88
## R^2 (amount of heterogeneity accounted for):            6.25%
##
## Test for Residual Heterogeneity:
## QE(df = 69) = 646.6613, p-val < .0001
##
## Test of Moderators (coefficient(s) 2):
## QM(df = 1) = 3.9005, p-val = 0.0483
##
## Model Results:
##
##          estimate      se     zval    pval    ci.lb    ci.ub
## intrcpt    0.2050  0.0715   2.8666  0.0041   0.0648   0.3452  **
## mods      -0.0105  0.0053  -1.9750  0.0483  -0.0209  -0.0001   *
##
## ---
## Signif. codes:  0 '***' 0.001 '**' 0.01 '*' 0.05 '.' 0.1 ' ' 1
```

```r
#RR estimate,lower and upper boundaries of the 95% CI
round(exp(c(education_all_overall$b,education_all_overall$ci.lb,education_all_overall$ci.ub)),digits=2)
```

```
## [1] 1.23 0.99 1.07 0.98 1.41 1.00
```

Whole group - Auditory Comprehension

```r
summary(data_ac$case_scholarity_years)
```

```
##    Min. 1st Qu.  Median    Mean 3rd Qu.    Max.    NA's
##    4.00   11.75   13.00   13.48   16.00   22.00      39
```

```r
print(education_all_ac<-rma(yi,vi,mods=case_scholarity_years,data=data_ac))
```

```
##
## Mixed-Effects Model (k = 52; tau^2 estimator: REML)
##
## tau^2 (estimated amount of residual heterogeneity):     0.0188 (SE = 0.0050)
## tau (square root of estimated tau^2 value):             0.1369
## I^2 (residual heterogeneity / unaccounted variability): 84.03%
## H^2 (unaccounted variability / sampling variability):   6.26
## R^2 (amount of heterogeneity accounted for):            0.00%
##
## Test for Residual Heterogeneity:
## QE(df = 50) = 231.8110, p-val < .0001
##
## Test of Moderators (coefficient(s) 2):
## QM(df = 1) = 0.1287, p-val = 0.7198
##
## Model Results:
##
##          estimate      se     zval    pval    ci.lb   ci.ub
## intrcpt    0.0527  0.0816   0.6460  0.5183  -0.1072  0.2126
## mods      -0.0020  0.0057  -0.3587  0.7198  -0.0131  0.0091
##
## ---
## Signif. codes:  0 '***' 0.001 '**' 0.01 '*' 0.05 '.' 0.1 ' ' 1
```

```r
#RR estimate,lower and upper boundaries of the 95% CI
round(exp(c(education_all_ac$b,education_all_ac$ci.lb,education_all_ac$ci.ub)),digits=2)
```

```
## [1] 1.05 1.00 0.90 0.99 1.24 1.01
```

Whole group - Oral Production

```r
summary(data_op$case_scholarity_years)
```

```
##    Min. 1st Qu.  Median    Mean 3rd Qu.    Max.    NA's
##    1.00   10.00   12.00   12.46   16.00   22.00      28
```

```r
print(education_all_op<-rma(yi,vi,mods=case_scholarity_years,data=data_op))
```

```
##
## Mixed-Effects Model (k = 63; tau^2 estimator: REML)
##
## tau^2 (estimated amount of residual heterogeneity):     0.0809 (SE = 0.0173)
## tau (square root of estimated tau^2 value):             0.2845
## I^2 (residual heterogeneity / unaccounted variability): 92.51%
## H^2 (unaccounted variability / sampling variability):   13.35
## R^2 (amount of heterogeneity accounted for):            5.34%
##
```

```
## Test for Residual Heterogeneity:
## QE(df = 61) = 478.0339, p-val < .0001
##
## Test of Moderators (coefficient(s) 2):
## QM(df = 1) = 3.0385, p-val = 0.0813
##
## Model Results:
##
##          estimate      se     zval    pval    ci.lb   ci.ub
## intrcpt    0.2434  0.1010   2.4106  0.0159   0.0455  0.4413  *
## mods      -0.0130  0.0075  -1.7431  0.0813  -0.0276  0.0016  .
##
## ---
## Signif. codes:  0 '***' 0.001 '**' 0.01 '*' 0.05 '.' 0.1 ' ' 1
```

```r
#RR estimate,lower and upper boundaries of the 95% CI
round(exp(c(education_all_op$b,education_all_op$ci.lb,education_all_op$ci.ub)),digits=2)
```

```
## [1] 1.28 0.99 1.05 0.97 1.55 1.00
```

Early subgroup - Overall Performance

```r
summary(data_e_overall$case_scholarity_years)
```

```
##    Min. 1st Qu.  Median    Mean 3rd Qu.    Max.    NA's
##    8.00   11.25   12.00   13.19   16.00   22.00      18
```

```r
print(education_early_overall<-rma(yi,vi,mods=case_scholarity_years,data=data_e_overall))
```

```
##
## Mixed-Effects Model (k = 26; tau^2 estimator: REML)
##
## tau^2 (estimated amount of residual heterogeneity):     0.0319 (SE = 0.0109)
## tau (square root of estimated tau^2 value):             0.1787
## I^2 (residual heterogeneity / unaccounted variability): 93.87%
## H^2 (unaccounted variability / sampling variability):   16.30
## R^2 (amount of heterogeneity accounted for):            0.00%
##
## Test for Residual Heterogeneity:
## QE(df = 24) = 168.4644, p-val < .0001
##
## Test of Moderators (coefficient(s) 2):
## QM(df = 1) = 0.7359, p-val = 0.3910
##
## Model Results:
##
##          estimate      se     zval    pval    ci.lb   ci.ub
## intrcpt    0.1185  0.1428   0.8298  0.4067  -0.1614  0.3983
## mods      -0.0089  0.0104  -0.8579  0.3910  -0.0293  0.0115
##
## ---
## Signif. codes:  0 '***' 0.001 '**' 0.01 '*' 0.05 '.' 0.1 ' ' 1
```

```r
#RR estimate,lower and upper boundaries of the 95% CI
round(exp(c(education_early_overall$b,education_early_overall$ci.lb,education_early_overall$ci.ub)),dig
```

```
## [1] 1.13 0.99 0.85 0.97 1.49 1.01
```

Early subgroup - Auditory Comprehension

```r
summary(data_e_ac$case_scholarity_years)
```

```
##    Min. 1st Qu.  Median    Mean 3rd Qu.    Max.    NA's
##    8.00   10.25   12.00   12.68   15.75   22.00      16
```

```r
print(education_early_ac<-rma(yi,vi,mods=case_scholarity_years,data=data_e_ac))
```

```
##
## Mixed-Effects Model (k = 22; tau^2 estimator: REML)
##
## tau^2 (estimated amount of residual heterogeneity):     0.0113 (SE = 0.0050)
## tau (square root of estimated tau^2 value):             0.1062
## I^2 (residual heterogeneity / unaccounted variability): 79.95%
## H^2 (unaccounted variability / sampling variability):   4.99
## R^2 (amount of heterogeneity accounted for):            0.00%
##
## Test for Residual Heterogeneity:
## QE(df = 20) = 70.4546, p-val < .0001
##
## Test of Moderators (coefficient(s) 2):
## QM(df = 1) = 0.0194, p-val = 0.8892
##
## Model Results:
##
##          estimate      se     zval    pval    ci.lb   ci.ub
## intrcpt    0.0124  0.1075   0.1157  0.9079  -0.1983  0.2232
## mods      -0.0011  0.0080  -0.1393  0.8892  -0.0169  0.0146
##
## ---
## Signif. codes:  0 '***' 0.001 '**' 0.01 '*' 0.05 '.' 0.1 ' ' 1
```

```r
#RR estimate,lower and upper boundaries of the 95% CI
round(exp(c(education_early_ac$b,education_early_ac$ci.lb,education_early_ac$ci.ub)),digits=2)
```

```
## [1] 1.01 1.00 0.82 0.98 1.25 1.01
```

Early subgroup - Oral Production

```r
summary(data_e_op$case_scholarity_years)
```

```
##    Min. 1st Qu.  Median    Mean 3rd Qu.    Max.    NA's 
##    9.00   12.00   14.00   14.26   16.00   22.00      13
```

```r
print(education_early_op<-rma(yi,vi,mods=case_scholarity_years,data=data_e_op))
```

```
## 
## Mixed-Effects Model (k = 19; tau^2 estimator: REML)
## 
## tau^2 (estimated amount of residual heterogeneity):     0.0878 (SE = 0.0346)
## tau (square root of estimated tau^2 value):             0.2964
## I^2 (residual heterogeneity / unaccounted variability): 94.84%
## H^2 (unaccounted variability / sampling variability):   19.39
## R^2 (amount of heterogeneity accounted for):            0.00%
## 
## Test for Residual Heterogeneity:
## QE(df = 17) = 146.8709, p-val < .0001
## 
## Test of Moderators (coefficient(s) 2):
## QM(df = 1) = 0.5990, p-val = 0.4389
## 
## Model Results:
## 
##          estimate      se     zval    pval    ci.lb   ci.ub 
## intrcpt    0.1972  0.3029   0.6509  0.5151  -0.3965  0.7909 
## mods      -0.0160  0.0206  -0.7740  0.4389  -0.0564  0.0245 
## 
## ---
## Signif. codes:  0 '***' 0.001 '**' 0.01 '*' 0.05 '.' 0.1 ' ' 1
```

```r
#RR estimate,lower and upper boundaries of the 95% CI
round(exp(c(education_early_op$b,education_early_op$ci.lb,education_early_op$ci.ub)),digits=2)
```

```
## [1] 1.22 0.98 0.67 0.95 2.21 1.02
```

Late subgroup - Overall Performance

```r
summary(data_l_overall$case_scholarity_years)
```

```
##    Min. 1st Qu.  Median    Mean 3rd Qu.    Max.    NA's 
##    1.00    6.00   12.00   11.69   16.00   22.00      30
```

```r
print(education_late_overall<-rma(yi,vi,mods=case_scholarity_years,data=data_l_overall))
```

```
## 
## Mixed-Effects Model (k = 45; tau^2 estimator: REML)
## 
## tau^2 (estimated amount of residual heterogeneity):     0.0450 (SE = 0.0114)
## tau (square root of estimated tau^2 value):             0.2122
## I^2 (residual heterogeneity / unaccounted variability): 92.13%
```

```
## H^2 (unaccounted variability / sampling variability):   12.71
## R^2 (amount of heterogeneity accounted for):            5.90%
##
## Test for Residual Heterogeneity:
## QE(df = 43) = 368.1597, p-val < .0001
##
## Test of Moderators (coefficient(s) 2):
## QM(df = 1) = 2.3614, p-val = 0.1244
##
## Model Results:
##
##          estimate      se     zval    pval    ci.lb   ci.ub
## intrcpt    0.2291  0.0817   2.8043  0.0050   0.0690  0.3892  **
## mods      -0.0094  0.0061  -1.5367  0.1244  -0.0215  0.0026
##
## ---
## Signif. codes:  0 '***' 0.001 '**' 0.01 '*' 0.05 '.' 0.1 ' ' 1
```

```
#RR estimate,lower and upper boundaries of the 95% CI
round(exp(c(education_late_overall$b,education_late_overall$ci.lb,education_late_overall$ci.ub)),digits=
```

```
## [1] 1.26 0.99 1.07 0.98 1.48 1.00
```

Late subgroup - Auditory Comprehension

```
summary(data_l_ac$case_scholarity_years)
```

```
##    Min. 1st Qu.  Median    Mean 3rd Qu.    Max.    NA's
##    4.00   12.00   14.50   14.07   16.00   22.00      23
```

```
print(education_late_ac<-rma(yi,vi,mods=case_scholarity_years,data=data_l_ac))
```

```
##
## Mixed-Effects Model (k = 30; tau^2 estimator: REML)
##
## tau^2 (estimated amount of residual heterogeneity):     0.0250 (SE = 0.0087)
## tau (square root of estimated tau^2 value):             0.1581
## I^2 (residual heterogeneity / unaccounted variability): 84.36%
## H^2 (unaccounted variability / sampling variability):   6.39
## R^2 (amount of heterogeneity accounted for):            0.00%
##
## Test for Residual Heterogeneity:
## QE(df = 28) = 138.3523, p-val < .0001
##
## Test of Moderators (coefficient(s) 2):
## QM(df = 1) = 0.4510, p-val = 0.5019
##
## Model Results:
##
##          estimate      se     zval    pval    ci.lb   ci.ub
```

```
## intrcpt    0.1227  0.1218   1.0073  0.3138  -0.1160  0.3613
## mods      -0.0054  0.0080  -0.6715  0.5019  -0.0212  0.0104
##
## ---
## Signif. codes:  0 '***' 0.001 '**' 0.01 '*' 0.05 '.' 0.1 ' ' 1
```

```
#RR estimate,lower and upper boundaries of the 95% CI
round(exp(c(education_late_ac$b,education_late_ac$ci.lb,education_late_ac$ci.ub)),digits=2)
```

```
## [1] 1.13 0.99 0.89 0.98 1.44 1.01
```

Late subgroup - Oral Production

```
summary(data_l_op$case_scholarity_years)
```

```
##    Min. 1st Qu.  Median    Mean 3rd Qu.    Max.    NA's
##    1.00    6.00   12.00   11.68   16.00   22.00      15
```

```
print(education_late_op<-rma(yi,vi,mods=case_scholarity_years,data=data_l_op))
```

```
##
## Mixed-Effects Model (k = 44; tau^2 estimator: REML)
##
## tau^2 (estimated amount of residual heterogeneity):     0.0746 (SE = 0.0196)
## tau (square root of estimated tau^2 value):             0.2731
## I^2 (residual heterogeneity / unaccounted variability): 89.62%
## H^2 (unaccounted variability / sampling variability):   9.63
## R^2 (amount of heterogeneity accounted for):            2.22%
##
## Test for Residual Heterogeneity:
## QE(df = 42) = 277.6920, p-val < .0001
##
## Test of Moderators (coefficient(s) 2):
## QM(df = 1) = 1.3867, p-val = 0.2390
##
## Model Results:
##
##          estimate      se     zval    pval    ci.lb   ci.ub
## intrcpt    0.2420  0.1041   2.3247  0.0201   0.0380  0.4460  *
## mods      -0.0094  0.0080  -1.1776  0.2390  -0.0251  0.0063
##
## ---
## Signif. codes:  0 '***' 0.001 '**' 0.01 '*' 0.05 '.' 0.1 ' ' 1
```

```
#RR estimate,lower and upper boundaries of the 95% CI
round(exp(c(education_late_op$b,education_late_op$ci.lb,education_late_op$ci.ub)),digits=2)
```

```
## [1] 1.27 0.99 1.04 0.98 1.56 1.01
```

Do months post onset moderate the difference betwen L1 and L2?

Whole group - Overall Performance

```r
summary(data$case_mpo)
```

```
##    Min. 1st Qu.  Median    Mean 3rd Qu.    Max.    NA's
##    1.00   15.00   29.00   28.33   43.00   53.00      13
```

```r
print(mpo_all_overall<-rma(yi,vi,mods=case_mpo,data=data))
```

```
##
## Mixed-Effects Model (k = 106; tau^2 estimator: REML)
##
## tau^2 (estimated amount of residual heterogeneity):     0.0500 (SE = 0.0082)
## tau (square root of estimated tau^2 value):             0.2237
## I^2 (residual heterogeneity / unaccounted variability): 93.76%
## H^2 (unaccounted variability / sampling variability):   16.02
## R^2 (amount of heterogeneity accounted for):            0.00%
##
## Test for Residual Heterogeneity:
## QE(df = 104) = 823.4685, p-val < .0001
##
## Test of Moderators (coefficient(s) 2):
## QM(df = 1) = 0.9744, p-val = 0.3236
##
## Model Results:
##
##          estimate      se    zval    pval    ci.lb   ci.ub
## intrcpt    0.0594  0.0501  1.1858  0.2357  -0.0387  0.1575
## mods       0.0015  0.0016  0.9871  0.3236  -0.0015  0.0046
##
## ---
## Signif. codes:  0 '***' 0.001 '**' 0.01 '*' 0.05 '.' 0.1 ' ' 1
```

```r
#RR estimate,lower and upper boundaries of the 95% CI
round(exp(c(mpo_all_overall$b,mpo_all_overall$ci.lb,mpo_all_overall$ci.ub)),digits=2)
```

```
## [1] 1.06 1.00 0.96 1.00 1.17 1.00
```

Whole group - Auditory Comprehension

```r
summary(data_ac$case_mpo)
```

```
##    Min. 1st Qu.  Median    Mean 3rd Qu.    Max.    NA's
##    2.00   15.00   29.00   28.33   42.50   53.00      12
```

```r
print(mpo_all_ac<-rma(yi,vi,mods=case_mpo,data=data_ac))
```

```
##
## Mixed-Effects Model (k = 79; tau^2 estimator: REML)
##
## tau^2 (estimated amount of residual heterogeneity):     0.0220 (SE = 0.0049)
## tau (square root of estimated tau^2 value):             0.1483
## I^2 (residual heterogeneity / unaccounted variability): 86.76%
## H^2 (unaccounted variability / sampling variability):   7.56
## R^2 (amount of heterogeneity accounted for):            0.00%
##
## Test for Residual Heterogeneity:
## QE(df = 77) = 319.8829, p-val < .0001
##
## Test of Moderators (coefficient(s) 2):
## QM(df = 1) = 1.1809, p-val = 0.2772
##
## Model Results:
##
##          estimate      se     zval    pval    ci.lb   ci.ub
## intrcpt    0.1051  0.0429   2.4478  0.0144   0.0209  0.1892  *
## mods      -0.0015  0.0013  -1.0867  0.2772  -0.0041  0.0012
##
## ---
## Signif. codes:  0 '***' 0.001 '**' 0.01 '*' 0.05 '.' 0.1 ' ' 1
```

```r
#RR estimate,lower and upper boundaries of the 95% CI
round(exp(c(mpo_all_ac$b,mpo_all_ac$ci.lb,mpo_all_ac$ci.ub)),digits=2)
```

```
## [1] 1.11 1.00 1.02 1.00 1.21 1.00
```

Whole group - Oral Production

```r
summary(data_op$case_mpo)
```

```
##    Min. 1st Qu.  Median    Mean 3rd Qu.    Max.    NA's
##    1.00   15.00   29.00   29.62   44.00   53.00       6
```

```r
print(mpo_all_op<-rma(yi,vi,mods=case_mpo,data=data_op))
```

```
##
## Mixed-Effects Model (k = 85; tau^2 estimator: REML)
##
## tau^2 (estimated amount of residual heterogeneity):     0.0647 (SE = 0.0122)
## tau (square root of estimated tau^2 value):             0.2543
## I^2 (residual heterogeneity / unaccounted variability): 91.15%
## H^2 (unaccounted variability / sampling variability):   11.30
## R^2 (amount of heterogeneity accounted for):            0.41%
##
## Test for Residual Heterogeneity:
```

```
## QE(df = 83) = 560.0567, p-val < .0001
##
## Test of Moderators (coefficient(s) 2):
## QM(df = 1) = 2.0369, p-val = 0.1535
##
## Model Results:
##
##          estimate      se    zval    pval    ci.lb   ci.ub
## intrcpt    0.0215  0.0681  0.3158  0.7521  -0.1120  0.1551
## mods       0.0029  0.0021  1.4272  0.1535  -0.0011  0.0070
##
## ---
## Signif. codes:  0 '***' 0.001 '**' 0.01 '*' 0.05 '.' 0.1 ' ' 1
```

```
#RR estimate,lower and upper boundaries of the 95% CI
round(exp(c(mpo_all_op$b,mpo_all_op$ci.lb,mpo_all_op$ci.ub)),digits=2)
```

```
## [1] 1.02 1.00 0.89 1.00 1.17 1.01
```

Early subgroup - Overearly Performance

```
summary(data_e_overall$case_mpo)
```

```
##    Min. 1st Qu.  Median    Mean 3rd Qu.    Max.    NA's
##    2.00   14.75   29.50   28.34   39.50   53.00      12
```

```
print(mpo_early_overall<-rma(yi,vi,mods=case_mpo,data=data_e_overall))
```

```
##
## Mixed-Effects Model (k = 32; tau^2 estimator: REML)
##
## tau^2 (estimated amount of residual heterogeneity):     0.0539 (SE = 0.0160)
## tau (square root of estimated tau^2 value):             0.2321
## I^2 (residual heterogeneity / unaccounted variability): 95.65%
## H^2 (unaccounted variability / sampling variability):   23.01
## R^2 (amount of heterogeneity accounted for):            0.09%
##
## Test for Residual Heterogeneity:
## QE(df = 30) = 253.2300, p-val < .0001
##
## Test of Moderators (coefficient(s) 2):
## QM(df = 1) = 1.3076, p-val = 0.2528
##
## Model Results:
##
##          estimate      se     zval    pval    ci.lb   ci.ub
## intrcpt   -0.1116  0.0930  -1.2001  0.2301  -0.2937  0.0706
## mods       0.0033  0.0029   1.1435  0.2528  -0.0024  0.0090
##
## ---
## Signif. codes:  0 '***' 0.001 '**' 0.01 '*' 0.05 '.' 0.1 ' ' 1
```

```r
#RR estimate,lower and upper boundaries of the 95% CI
round(exp(c(mpo_early_overall$b,mpo_early_overall$ci.lb,mpo_early_overall$ci.ub)),digits=2)
```

```
## [1] 0.89 1.00 0.75 1.00 1.07 1.01
```

Early subgroup - Auditory Comprehension

```r
summary(data_e_ac$case_mpo)
```

```
##    Min. 1st Qu.  Median    Mean 3rd Qu.    Max.    NA's
##    2.00   14.50   29.00   28.22   40.00   53.00      11
```

```r
print(mpo_early_ac<-rma(yi,vi,mods=case_mpo,data=data_e_ac))
```

```
##
## Mixed-Effects Model (k = 27; tau^2 estimator: REML)
##
## tau^2 (estimated amount of residual heterogeneity):     0.0218 (SE = 0.0080)
## tau (square root of estimated tau^2 value):             0.1476
## I^2 (residual heterogeneity / unaccounted variability): 88.13%
## H^2 (unaccounted variability / sampling variability):   8.42
## R^2 (amount of heterogeneity accounted for):            0.00%
##
## Test for Residual Heterogeneity:
## QE(df = 25) = 103.0558, p-val < .0001
##
## Test of Moderators (coefficient(s) 2):
## QM(df = 1) = 0.7140, p-val = 0.3981
##
## Model Results:
##
##          estimate      se     zval    pval    ci.lb   ci.ub
## intrcpt    0.0635  0.0675   0.9402  0.3471  -0.0689  0.1958
## mods      -0.0018  0.0022  -0.8450  0.3981  -0.0061  0.0024
##
## ---
## Signif. codes:  0 '***' 0.001 '**' 0.01 '*' 0.05 '.' 0.1 ' ' 1
```

```r
#RR estimate,lower and upper boundaries of the 95% CI
round(exp(c(mpo_early_ac$b,mpo_early_ac$ci.lb,mpo_early_ac$ci.ub)),digits=2)
```

```
## [1] 1.07 1.00 0.93 0.99 1.22 1.00
```

Early subgroup - Oral Production

```r
summary(data_e_op$case_mpo)
```

```
##    Min. 1st Qu.  Median    Mean 3rd Qu.    Max.    NA's
##    2.00   14.50   27.00   26.96   38.50   53.00       5
```

```r
print(mpo_early_op<-rma(yi,vi,mods=case_mpo,data=data_e_op))
```

```
##
## Mixed-Effects Model (k = 27; tau^2 estimator: REML)
##
## tau^2 (estimated amount of residual heterogeneity):     0.0562 (SE = 0.0192)
## tau (square root of estimated tau^2 value):             0.2371
## I^2 (residual heterogeneity / unaccounted variability): 93.09%
## H^2 (unaccounted variability / sampling variability):   14.47
## R^2 (amount of heterogeneity accounted for):            6.98%
##
## Test for Residual Heterogeneity:
## QE(df = 25) = 173.8255, p-val < .0001
##
## Test of Moderators (coefficient(s) 2):
## QM(df = 1) = 3.1476, p-val = 0.0760
##
## Model Results:
##
##          estimate      se     zval    pval    ci.lb   ci.ub
## intrcpt   -0.1842  0.1028  -1.7915  0.0732  -0.3857  0.0173  .
## mods       0.0058  0.0033   1.7741  0.0760  -0.0006  0.0123  .
##
## ---
## Signif. codes:  0 '***' 0.001 '**' 0.01 '*' 0.05 '.' 0.1 ' ' 1
```

```r
#RR estimate,lower and upper boundaries of the 95% CI
round(exp(c(mpo_early_op$b,mpo_early_op$ci.lb,mpo_early_op$ci.ub)),digits=2)
```

```
## [1] 0.83 1.01 0.68 1.00 1.02 1.01
```

Late subgroup - Overlate Performance

```r
summary(data_l_overall$case_mpo)
```

```
##    Min. 1st Qu.  Median    Mean 3rd Qu.    Max.    NA's
##    1.00   15.00   29.00   28.32   43.00   52.00       1
```

```r
print(mpo_late_overall<-rma(yi,vi,mods=case_mpo,data=data_l_overall))
```

```
##
## Mixed-Effects Model (k = 74; tau^2 estimator: REML)
##
## tau^2 (estimated amount of residual heterogeneity):     0.0364 (SE = 0.0076)
## tau (square root of estimated tau^2 value):             0.1909
## I^2 (residual heterogeneity / unaccounted variability): 89.61%
## H^2 (unaccounted variability / sampling variability):   9.62
## R^2 (amount of heterogeneity accounted for):            0.00%
##
```

```
## Test for Residual Heterogeneity:
## QE(df = 72) = 430.7715, p-val < .0001
##
## Test of Moderators (coefficient(s) 2):
## QM(df = 1) = 0.1619, p-val = 0.6874
##
## Model Results:
##
##          estimate      se    zval    pval    ci.lb   ci.ub
## intrcpt    0.1360  0.0527  2.5805  0.0099   0.0327  0.2392  **
## mods       0.0007  0.0016  0.4024  0.6874  -0.0026  0.0039
##
## ---
## Signif. codes:  0 '***' 0.001 '**' 0.01 '*' 0.05 '.' 0.1 ' ' 1
```

```
#RR estimate,lower and upper boundaries of the 95% CI
round(exp(c(mpo_late_overall$b,mpo_late_overall$ci.lb,mpo_late_overall$ci.ub)),digits=2)
```

```
## [1] 1.15 1.00 1.03 1.00 1.27 1.00
```

Late subgroup - Auditory Comprehension

```
summary(data_l_ac$case_mpo)
```

```
##    Min. 1st Qu.  Median    Mean 3rd Qu.    Max.    NA's
##    2.00   15.00   30.00   28.38   43.00   52.00       1
```

```
print(mpo_late_ac<-rma(yi,vi,mods=case_mpo,data=data_l_ac))
```

```
##
## Mixed-Effects Model (k = 52; tau^2 estimator: REML)
##
## tau^2 (estimated amount of residual heterogeneity):     0.0203 (SE = 0.0060)
## tau (square root of estimated tau^2 value):             0.1425
## I^2 (residual heterogeneity / unaccounted variability): 83.87%
## H^2 (unaccounted variability / sampling variability):   6.20
## R^2 (amount of heterogeneity accounted for):            0.00%
##
## Test for Residual Heterogeneity:
## QE(df = 50) = 183.1323, p-val < .0001
##
## Test of Moderators (coefficient(s) 2):
## QM(df = 1) = 0.7866, p-val = 0.3751
##
## Model Results:
##
##          estimate      se     zval    pval    ci.lb   ci.ub
## intrcpt    0.1362  0.0540   2.5205  0.0117   0.0303  0.2421  *
## mods      -0.0015  0.0017  -0.8869  0.3751  -0.0047  0.0018
##
## ---
## Signif. codes:  0 '***' 0.001 '**' 0.01 '*' 0.05 '.' 0.1 ' ' 1
```

```r
#RR estimate,lower and upper boundaries of the 95% CI
round(exp(c(mpo_late_ac$b,mpo_late_ac$ci.lb,mpo_late_ac$ci.ub)),digits=2)
```

```
## [1] 1.15 1.00 1.03 1.00 1.27 1.00
```

Late subgroup - Oral Production

```r
summary(data_l_op$case_mpo)
```

```
##    Min. 1st Qu.  Median    Mean 3rd Qu.    Max.    NA's
##    1.00   19.50   30.00   30.86   45.00   52.00       1
```

```r
print(mpo_late_op<-rma(yi,vi,mods=case_mpo,data=data_l_op))
```

```
##
## Mixed-Effects Model (k = 58; tau^2 estimator: REML)
##
## tau^2 (estimated amount of residual heterogeneity):     0.0518 (SE = 0.0125)
## tau (square root of estimated tau^2 value):             0.2276
## I^2 (residual heterogeneity / unaccounted variability): 85.59%
## H^2 (unaccounted variability / sampling variability):   6.94
## R^2 (amount of heterogeneity accounted for):            0.00%
##
## Test for Residual Heterogeneity:
## QE(df = 56) = 295.0171, p-val < .0001
##
## Test of Moderators (coefficient(s) 2):
## QM(df = 1) = 0.0089, p-val = 0.9249
##
## Model Results:
##
##          estimate      se    zval    pval    ci.lb   ci.ub
## intrcpt    0.1667  0.0795  2.0961  0.0361   0.0108  0.3225  *
## mods       0.0002  0.0023  0.0943  0.9249  -0.0044  0.0048
##
## ---
## Signif. codes:  0 '***' 0.001 '**' 0.01 '*' 0.05 '.' 0.1 ' ' 1
```

```r
#RR estimate,lower and upper boundaries of the 95% CI
round(exp(c(mpo_late_op$b,mpo_late_op$ci.lb,mpo_late_op$ci.ub)),digits=2)
```

```
## [1] 1.18 1.00 1.01 1.00 1.38 1.00
```

Does gender moderate the difference betwen L1 and L2?

Whole group - Overall Performance

```
summary(data$case_gender)
```

```
##  f  m
## 57 62
```

```
data$male  <-ifelse(data$case_gender=="m",1,0)
data$female<-ifelse(data$case_gender=="f",1,0)
```

```
print(m_gender<-rma(yi,vi,mods=cbind(male,female),intercept=F,data=data))
```

```
##
## Mixed-Effects Model (k = 119; tau^2 estimator: REML)
##
## tau^2 (estimated amount of residual heterogeneity):     0.0522 (SE = 0.0080)
## tau (square root of estimated tau^2 value):             0.2285
## I^2 (residual heterogeneity / unaccounted variability): 94.87%
## H^2 (unaccounted variability / sampling variability):   19.49
##
## Test for Residual Heterogeneity:
## QE(df = 117) = 1013.8464, p-val < .0001
##
## Test of Moderators (coefficient(s) 1:2):
## QM(df = 2) = 16.6837, p-val = 0.0002
##
## Model Results:
##
##         estimate      se    zval    pval   ci.lb   ci.ub
## male      0.1002  0.0314  3.1875  0.0014  0.0386  0.1619  **
## female    0.0850  0.0333  2.5542  0.0106  0.0198  0.1502   *
##
## ---
## Signif. codes:  0 '***' 0.001 '**' 0.01 '*' 0.05 '.' 0.1 ' ' 1
```

```
#RR estimate  lower and upper boundaries of the 95% CI
round(exp(c(m_gender$b,m_gender$ci.lb,m_gender$ci.ub)),digits=2)
```

```
## [1] 1.11 1.09 1.04 1.02 1.18 1.16
```

Checking whether linguistic similarity is a significant moderator.

```
capture.output(rma(yi,vi,mods=cbind(male,female),data=data))[c(8,10,11,13,14)]
```

```
## [1] "R^2 (amount of heterogeneity accounted for):            0.00%"
## [2] "Test for Residual Heterogeneity: "
## [3] "QE(df = 117) = 1013.8464, p-val < .0001"
## [4] "Test of Moderators (coefficient(s) 2): "
## [5] "QM(df = 1) = 0.1110, p-val = 0.7390"
```

Whole group - Auditory Comprehension

```r
summary(data_ac$case_gender)
```

```
##  f  m
## 47 44
```

```r
data_ac$male  <-ifelse(data_ac$case_gender=="m",1,0)
data_ac$female<-ifelse(data_ac$case_gender=="f",1,0)
```

```r
print(m35<-rma(yi,vi,mods=cbind(male,female),intercept=F,data=data_ac))
```

```
##
## Mixed-Effects Model (k = 91; tau^2 estimator: REML)
##
## tau^2 (estimated amount of residual heterogeneity):     0.0222 (SE = 0.0046)
## tau (square root of estimated tau^2 value):             0.1491
## I^2 (residual heterogeneity / unaccounted variability): 86.52%
## H^2 (unaccounted variability / sampling variability):   7.42
##
## Test for Residual Heterogeneity:
## QE(df = 89) = 353.2968, p-val < .0001
##
## Test of Moderators (coefficient(s) 1:2):
## QM(df = 2) = 11.0020, p-val = 0.0041
##
## Model Results:
##
##         estimate      se    zval    pval    ci.lb   ci.ub
## male      0.0758  0.0264  2.8756  0.0040   0.0241  0.1274  **
## female    0.0449  0.0272  1.6533  0.0983  -0.0083  0.0981   .
##
## ---
## Signif. codes:  0 '***' 0.001 '**' 0.01 '*' 0.05 '.' 0.1 ' ' 1
```

```r
#RR estimate  lower and upper boundaries of the 95% CI
round(exp(c(m35$b,m35$ci.lb,m35$ci.ub)),digits=2)
```

```
## [1] 1.08 1.05 1.02 0.99 1.14 1.10
```

Checking whether linguistic similarity is a significant moderator.

```r
capture.output(rma(yi,vi,mods=cbind(male,female),data=data_ac))[c(8,10,11,13,14)]
```

```
## [1] "R^2 (amount of heterogeneity accounted for):            1.23%"
## [2] "Test for Residual Heterogeneity: "
## [3] "QE(df = 89) = 353.2968, p-val < .0001"
## [4] "Test of Moderators (coefficient(s) 2): "
## [5] "QM(df = 1) = 0.6657, p-val = 0.4146"
```

Whole group - Oral Production

```r
summary(data_op$case_gender)
```

```
##  f  m
## 42 49
```

```r
data_op$male  <-ifelse(data_op$case_gender=="m",1,0)
data_op$female<-ifelse(data_op$case_gender=="f",1,0)
```

```r
print(m36<-rma(yi,vi,mods=cbind(male,female),intercept=F,data=data_op))
```

```
##
## Mixed-Effects Model (k = 91; tau^2 estimator: REML)
##
## tau^2 (estimated amount of residual heterogeneity):     0.0752 (SE = 0.0133)
## tau (square root of estimated tau^2 value):             0.2743
## I^2 (residual heterogeneity / unaccounted variability): 93.60%
## H^2 (unaccounted variability / sampling variability):   15.62
##
## Test for Residual Heterogeneity:
## QE(df = 89) = 668.9459, p-val < .0001
##
## Test of Moderators (coefficient(s) 1:2):
## QM(df = 2) = 11.3187, p-val = 0.0035
##
## Model Results:
##
##         estimate      se    zval    pval    ci.lb   ci.ub
## male      0.1401  0.0427  3.2782  0.0010   0.0563  0.2239  **
## female    0.0352  0.0465  0.7565  0.4494  -0.0560  0.1264
##
## ---
## Signif. codes:  0 '***' 0.001 '**' 0.01 '*' 0.05 '.' 0.1 ' ' 1
```

```r
#RR estimate  lower and upper boundaries of the 95% CI
round(exp(c(m36$b,m36$ci.lb,m36$ci.ub)),digits=2)
```

```
## [1] 1.15 1.04 1.06 0.95 1.25 1.13
```

Checking whether linguistic similarity is a significant moderator.

```r
capture.output(rma(yi,vi,mods=cbind(male,female),data=data_op))[c(8,10,11,13,14)]
```

```
## [1] "R^2 (amount of heterogeneity accounted for):            1.99%"
## [2] "Test for Residual Heterogeneity: "
## [3] "QE(df = 89) = 668.9459, p-val < .0001"
## [4] "Test of Moderators (coefficient(s) 2): "
## [5] "QM(df = 1) = 2.7588, p-val = 0.0967"
```

Early subgroup - Overall Performance

```
summary(data_e_overall$case_gender)
```

```
##  f  m
## 21 23
```

```
data_e_overall$male  <-ifelse(data_e_overall$case_gender=="m",1,0)
data_e_overall$female<-ifelse(data_e_overall$case_gender=="f",1,0)
```

```
print(m37<-rma(yi,vi,mods=cbind(male,female),intercept=F,data=data_e_overall))
```

```
##
## Mixed-Effects Model (k = 44; tau^2 estimator: REML)
##
## tau^2 (estimated amount of residual heterogeneity):     0.0565 (SE = 0.0139)
## tau (square root of estimated tau^2 value):             0.2377
## I^2 (residual heterogeneity / unaccounted variability): 96.76%
## H^2 (unaccounted variability / sampling variability):   30.86
##
## Test for Residual Heterogeneity:
## QE(df = 42) = 436.2570, p-val < .0001
##
## Test of Moderators (coefficient(s) 1:2):
## QM(df = 2) = 0.1017, p-val = 0.9504
##
## Model Results:
##
##         estimate      se     zval    pval    ci.lb   ci.ub
## male     -0.0113  0.0533  -0.2125  0.8317  -0.1157  0.0931
## female    0.0131  0.0552   0.2377  0.8121  -0.0950  0.1212
##
## ---
## Signif. codes:  0 '***' 0.001 '**' 0.01 '*' 0.05 '.' 0.1 ' ' 1
```

```
#RR estimate  lower and upper boundaries of the 95% CI
round(exp(c(m37$b,m37$ci.lb,m37$ci.ub)),digits=2)
```

```
## [1] 0.99 1.01 0.89 0.91 1.10 1.13
```

Checking whether linguistic similarity is a significant moderator.

```
capture.output(rma(yi,vi,mods=cbind(male,female),data=data_e_overall))[c(8,10,11,13,14)]
```

```
## [1] "R^2 (amount of heterogeneity accounted for):            0.00%"
## [2] "Test for Residual Heterogeneity: "
## [3] "QE(df = 42) = 436.2570, p-val < .0001"
## [4] "Test of Moderators (coefficient(s) 2): "
## [5] "QM(df = 1) = 0.1015, p-val = 0.7500"
```

Early subgroup - Auditory Comprehension

```r
summary(data_e_ac$case_gender)
```

```
##  f  m
## 18 20
```

```r
data_e_ac$male  <-ifelse(data_e_ac$case_gender=="m",1,0)
data_e_ac$female<-ifelse(data_e_ac$case_gender=="f",1,0)
```

```r
print(m38<-rma(yi,vi,mods=cbind(male,female),intercept=F,data=data_e_ac))
```

```
##
## Mixed-Effects Model (k = 38; tau^2 estimator: REML)
##
## tau^2 (estimated amount of residual heterogeneity):     0.0205 (SE = 0.0066)
## tau (square root of estimated tau^2 value):             0.1432
## I^2 (residual heterogeneity / unaccounted variability): 86.30%
## H^2 (unaccounted variability / sampling variability):   7.30
##
## Test for Residual Heterogeneity:
## QE(df = 36) = 127.9624, p-val < .0001
##
## Test of Moderators (coefficient(s) 1:2):
## QM(df = 2) = 1.8248, p-val = 0.4016
##
## Model Results:
##
##         estimate      se     zval    pval    ci.lb   ci.ub
## male      0.0507  0.0376   1.3498  0.1771  -0.0229  0.1244
## female   -0.0021  0.0405  -0.0530  0.9577  -0.0815  0.0772
##
## ---
## Signif. codes:  0 '***' 0.001 '**' 0.01 '*' 0.05 '.' 0.1 ' ' 1
```

```r
#RR estimate  lower and upper boundaries of the 95% CI
round(exp(c(m38$b,m38$ci.lb,m38$ci.ub)),digits=2)
```

```
## [1] 1.05 1.00 0.98 0.92 1.13 1.08
```

Checking whether linguistic similarity is a significant moderator.

```r
capture.output(rma(yi,vi,mods=cbind(male,female),data=data_e_ac))[c(8,10,11,13,14)]
```

```
## [1] "R^2 (amount of heterogeneity accounted for):            3.43%"
## [2] "Test for Residual Heterogeneity: "
## [3] "QE(df = 36) = 127.9624, p-val < .0001"
## [4] "Test of Moderators (coefficient(s) 2): "
## [5] "QM(df = 1) = 0.9165, p-val = 0.3384"
```

Early subgroup - Oral Production

```
summary(data_e_op$case_gender)
```

```
##  f  m
## 17 15
```

```
data_e_op$male  <-ifelse(data_e_op$case_gender=="m",1,0)
data_e_op$female<-ifelse(data_e_op$case_gender=="f",1,0)
```

```
print(m39<-rma(yi,vi,mods=cbind(male,female),intercept=F,data=data_e_op))
```

```
##
## Mixed-Effects Model (k = 32; tau^2 estimator: REML)
##
## tau^2 (estimated amount of residual heterogeneity):     0.0835 (SE = 0.0245)
## tau (square root of estimated tau^2 value):             0.2889
## I^2 (residual heterogeneity / unaccounted variability): 96.46%
## H^2 (unaccounted variability / sampling variability):   28.25
##
## Test for Residual Heterogeneity:
## QE(df = 30) = 280.3992, p-val < .0001
##
## Test of Moderators (coefficient(s) 1:2):
## QM(df = 2) = 0.3044, p-val = 0.8588
##
## Model Results:
##
##         estimate      se     zval    pval    ci.lb   ci.ub
## male     -0.0076  0.0801  -0.0945  0.9247  -0.1646  0.1494
## female   -0.0407  0.0748  -0.5436  0.5867  -0.1873  0.1060
##
## ---
## Signif. codes:  0 '***' 0.001 '**' 0.01 '*' 0.05 '.' 0.1 ' ' 1
```

```
#RR estimate  lower and upper boundaries of the 95% CI
round(exp(c(m39$b,m39$ci.lb,m39$ci.ub)),digits=2)
```

```
## [1] 0.99 0.96 0.85 0.83 1.16 1.11
```

Checking whether linguistic similarity is a significant moderator.

```
capture.output(rma(yi,vi,mods=cbind(male,female),data=data_e_op))[c(8,10,11,13,14)]
```

```
## [1] "R^2 (amount of heterogeneity accounted for):            0.00%"
## [2] "Test for Residual Heterogeneity: "
## [3] "QE(df = 30) = 280.3992, p-val < .0001"
## [4] "Test of Moderators (coefficient(s) 2): "
## [5] "QM(df = 1) = 0.0912, p-val = 0.7627"
```

Late subgroup - Overall Performance

```
summary(data_l_overall$case_gender)
```

```
##  f  m
## 36 39
```

```
data_l_overall$male  <-ifelse(data_l_overall$case_gender=="m",1,0)
data_l_overall$female<-ifelse(data_l_overall$case_gender=="f",1,0)
```

```
print(m40<-rma(yi,vi,mods=cbind(male,female),intercept=F,data=data_l_overall))
```

```
##
## Mixed-Effects Model (k = 75; tau^2 estimator: REML)
##
## tau^2 (estimated amount of residual heterogeneity):     0.0401 (SE = 0.0082)
## tau (square root of estimated tau^2 value):             0.2002
## I^2 (residual heterogeneity / unaccounted variability): 90.48%
## H^2 (unaccounted variability / sampling variability):   10.50
##
## Test for Residual Heterogeneity:
## QE(df = 73) = 469.8867, p-val < .0001
##
## Test of Moderators (coefficient(s) 1:2):
## QM(df = 2) = 32.8968, p-val < .0001
##
## Model Results:
##
##         estimate      se    zval    pval   ci.lb   ci.ub
## male      0.1649  0.0355  4.6485  <.0001  0.0954  0.2345  ***
## female    0.1285  0.0382  3.3598  0.0008  0.0535  0.2034  ***
##
## ---
## Signif. codes:  0 '***' 0.001 '**' 0.01 '*' 0.05 '.' 0.1 ' ' 1
```

```
#RR estimate  lower and upper boundaries of the 95% CI
round(exp(c(m40$b,m40$ci.lb,m40$ci.ub)),digits=2)
```

```
## [1] 1.18 1.14 1.10 1.05 1.26 1.23
```

Checking whether linguistic similarity is a significant moderator.

```
capture.output(rma(yi,vi,mods=cbind(male,female),data=data_l_overall))[c(8,10,11,13,14)]
```

```
## [1] "R^2 (amount of heterogeneity accounted for):            0.00%"
## [2] "Test for Residual Heterogeneity: "
## [3] "QE(df = 73) = 469.8867, p-val < .0001"
## [4] "Test of Moderators (coefficient(s) 2): "
## [5] "QM(df = 1) = 0.4876, p-val = 0.4850"
```

Late subgroup - Auditory Comprehension

```r
summary(data_l_ac$case_gender)
```

```
##  f  m
## 29 24
```

```r
data_l_ac$male  <-ifelse(data_l_ac$case_gender=="m",1,0)
data_l_ac$female<-ifelse(data_l_ac$case_gender=="f",1,0)
```

```r
print(m41<-rma(yi,vi,mods=cbind(male,female),intercept=F,data=data_l_ac))
```

```
##
## Mixed-Effects Model (k = 53; tau^2 estimator: REML)
##
## tau^2 (estimated amount of residual heterogeneity):     0.0226 (SE = 0.0065)
## tau (square root of estimated tau^2 value):             0.1503
## I^2 (residual heterogeneity / unaccounted variability): 84.76%
## H^2 (unaccounted variability / sampling variability):   6.56
##
## Test for Residual Heterogeneity:
## QE(df = 51) = 199.5421, p-val < .0001
##
## Test of Moderators (coefficient(s) 1:2):
## QM(df = 2) = 12.0123, p-val = 0.0025
##
## Model Results:
##
##         estimate      se    zval    pval   ci.lb   ci.ub
## male      0.0971  0.0361  2.6873  0.0072  0.0263  0.1679  **
## female    0.0787  0.0360  2.1887  0.0286  0.0082  0.1492   *
##
## ---
## Signif. codes:  0 '***' 0.001 '**' 0.01 '*' 0.05 '.' 0.1 ' ' 1
```

```r
#RR estimate  lower and upper boundaries of the 95% CI
round(exp(c(m41$b,m41$ci.lb,m41$ci.ub)),digits=2)
```

```
## [1] 1.10 1.08 1.03 1.01 1.18 1.16
```

Checking whether linguistic similarity is a significant moderator.

```r
capture.output(rma(yi,vi,mods=cbind(male,female),data=data_l_ac))[c(8,10,11,13,14)]
```

```
## [1] "R^2 (amount of heterogeneity accounted for):            0.00%"
## [2] "Test for Residual Heterogeneity: "
## [3] "QE(df = 51) = 199.5421, p-val < .0001"
## [4] "Test of Moderators (coefficient(s) 2): "
## [5] "QM(df = 1) = 0.1301, p-val = 0.7183"
```

Late subgroup - Oral Production

```
summary(data_l_op$case_gender)
```

```
##  f  m
## 25 34
```

```
data_l_op$male  <-ifelse(data_l_op$case_gender=="m",1,0)
data_l_op$female<-ifelse(data_l_op$case_gender=="f",1,0)
```

```
print(m42<-rma(yi,vi,mods=cbind(male,female),intercept=F,data=data_l_op))
```

```
##
## Mixed-Effects Model (k = 59; tau^2 estimator: REML)
##
## tau^2 (estimated amount of residual heterogeneity):     0.0591 (SE = 0.0138)
## tau (square root of estimated tau^2 value):             0.2431
## I^2 (residual heterogeneity / unaccounted variability): 86.99%
## H^2 (unaccounted variability / sampling variability):   7.69
##
## Test for Residual Heterogeneity:
## QE(df = 57) = 321.7174, p-val < .0001
##
## Test of Moderators (coefficient(s) 1:2):
## QM(df = 2) = 22.5882, p-val < .0001
##
## Model Results:
##
##         estimate      se    zval    pval    ci.lb   ci.ub
## male      0.2068  0.0466  4.4404  <.0001   0.1155  0.2981  ***
## female    0.0941  0.0555  1.6943  0.0902  -0.0148  0.2030    .
##
## ---
## Signif. codes:  0 '***' 0.001 '**' 0.01 '*' 0.05 '.' 0.1 ' ' 1
```

```
round(exp(c(m42$b,m42$ci.lb,m42$ci.ub)),digits=2)
```

```
## [1] 1.23 1.10 1.12 0.99 1.35 1.23
```

Checking whether linguistic similarity is a significant moderator.

```
capture.output(rma(yi,vi,mods=cbind(male,female),data=data_l_op))[c(8,10,11,13,14)]
```

```
## [1] "R^2 (amount of heterogeneity accounted for):            1.78%"
## [2] "Test for Residual Heterogeneity: "
## [3] "QE(df = 57) = 321.7174, p-val < .0001"
## [4] "Test of Moderators (coefficient(s) 2): "
## [5] "QM(df = 1) = 2.4168, p-val = 0.1200"
```

Does the research question type moderate the difference betwen L1 and L2??

Overall Performance - Whole group

```
data$rq_rel  <-ifelse(data$study_rq=="yes"|data$study_rq=="yes_rep",1,0)
data$rq_unrel<-ifelse(data$study_rq=="no"|data$study_rq=="no_rep",1,0)
```

```
print(c(sum(data$rq_rel=="1"),sum(data$rq_unrel=="1")))
```

```
## [1] 24 30
```

```
print(m_rq_overall<-rma(yi,vi,mods=cbind(rq_rel,rq_unrel),intercept=F,data=data))
```

```
##
## Mixed-Effects Model (k = 119; tau^2 estimator: REML)
##
## tau^2 (estimated amount of residual heterogeneity):     0.0563 (SE = 0.0086)
## tau (square root of estimated tau^2 value):             0.2372
## I^2 (residual heterogeneity / unaccounted variability): 95.32%
## H^2 (unaccounted variability / sampling variability):   21.35
##
## Test for Residual Heterogeneity:
## QE(df = 117) = 1043.7910, p-val < .0001
##
## Test of Moderators (coefficient(s) 1:2):
## QM(df = 2) = 9.0858, p-val = 0.0106
##
## Model Results:
##
##            estimate      se    zval    pval    ci.lb   ci.ub
## rq_rel       0.0253  0.0513  0.4936  0.6216  -0.0753  0.1259
## rq_unrel     0.1381  0.0464  2.9736  0.0029   0.0471  0.2291  **
##
## ---
## Signif. codes:  0 '***' 0.001 '**' 0.01 '*' 0.05 '.' 0.1 ' ' 1
```

```
#RR estimate,lower and upper boundaries of the 95% CI
round(exp(c(m_rq_overall$b,m_rq_overall$ci.lb,m_rq_overall$ci.ub)),digits=2)
```

```
## [1] 1.03 1.15 0.93 1.05 1.13 1.26
```

Checking whether the research question is a significant moderator.

```
capture.output(rma(yi,vi,mods=cbind(rq_rel,rq_unrel),data=data))[c(8,10,11,13,14)]
```

```
## [1] "R^2 (amount of heterogeneity accounted for):            0.36%"
## [2] "Test for Residual Heterogeneity: "
## [3] "QE(df = 116) = 1006.9518, p-val < .0001"
## [4] "Test of Moderators (coefficient(s) 2:3): "
## [5] "QM(df = 2) = 2.8892, p-val = 0.2358"
```

Auditory comprehension - Whole group

```
data_ac$rq_rel  <-ifelse(data_ac$study_rq=="yes"|data_ac$study_rq=="yes_rep",1,0)
data_ac$rq_unrel<-ifelse(data_ac$study_rq=="no"|data_ac$study_rq=="no_rep",1,0)
```

```
print(c(sum(data_ac$rq_rel=="1"), sum(data_ac$rq_unrel=="1")))
```

```
## [1] 21 23
```

```
print(m_rq_ac<-rma(yi,vi,mods=cbind(rq_rel,rq_unrel),intercept=F,data=data_ac))
```

```
##
## Mixed-Effects Model (k = 91; tau^2 estimator: REML)
##
## tau^2 (estimated amount of residual heterogeneity):     0.0244 (SE = 0.0050)
## tau (square root of estimated tau^2 value):             0.1563
## I^2 (residual heterogeneity / unaccounted variability): 87.40%
## H^2 (unaccounted variability / sampling variability):   7.94
##
## Test for Residual Heterogeneity:
## QE(df = 89) = 363.9116, p-val < .0001
##
## Test of Moderators (coefficient(s) 1:2):
## QM(df = 2) = 4.7609, p-val = 0.0925
##
## Model Results:
##
##           estimate      se    zval    pval    ci.lb   ci.ub
## rq_rel      0.0459  0.0402  1.1425  0.2533  -0.0329  0.1247
## rq_unrel    0.0692  0.0372  1.8590  0.0630  -0.0038  0.1421  .
##
## ---
## Signif. codes:  0 '***' 0.001 '**' 0.01 '*' 0.05 '.' 0.1 ' ' 1
```

```
#RR estimate,lower and upper boundaries of the 95% CI
round(exp(c(m_rq_ac$b,m_rq_ac$ci.lb,m_rq_ac$ci.ub)),digits=2)
```

```
## [1] 1.05 1.07 0.97 1.00 1.13 1.15
```

Checking whether the research question is a significant moderator.

```
capture.output(rma(yi,vi,mods=cbind(rq_rel ,rq_unrel),data=data_ac))[c(8,10,11,13,14)]
```

```
## [1] "R^2 (amount of heterogeneity accounted for):            0.00%"
## [2] "Test for Residual Heterogeneity: "
## [3] "QE(df = 88) = 359.5618, p-val < .0001"
## [4] "Test of Moderators (coefficient(s) 2:3): "
## [5] "QM(df = 2) = 0.2072, p-val = 0.9016"
```

Oral production - Whole group

```
data_op$rq_rel  <-ifelse(data_op$study_rq=="yes"|data_op$study_rq=="yes_rep",1,0)
data_op$rq_unrel<-ifelse(data_op$study_rq=="no"|data_op$study_rq=="no_rep",1,0)
```

```
print(c(sum(data_op$rq_rel=="1"), sum(data_op$rq_unrel=="1")))
```

```
## [1] 20 27
```

```
print(m_rq_oral<-rma(yi,vi,mods=cbind(rq_rel,rq_unrel),intercept=F,data=data_op))
```

```
##
## Mixed-Effects Model (k = 91; tau^2 estimator: REML)
##
## tau^2 (estimated amount of residual heterogeneity):     0.0830 (SE = 0.0145)
## tau (square root of estimated tau^2 value):             0.2881
## I^2 (residual heterogeneity / unaccounted variability): 94.42%
## H^2 (unaccounted variability / sampling variability):   17.92
##
## Test for Residual Heterogeneity:
## QE(df = 89) = 708.0430, p-val < .0001
##
## Test of Moderators (coefficient(s) 1:2):
## QM(df = 2) = 3.9045, p-val = 0.1420
##
## Model Results:
##
##           estimate      se    zval    pval    ci.lb   ci.ub
## rq_rel      0.0404  0.0694  0.5831  0.5598  -0.0955  0.1764
## rq_unrel    0.1136  0.0602  1.8880  0.0590  -0.0043  0.2315  .
##
## ---
## Signif. codes:  0 '***' 0.001 '**' 0.01 '*' 0.05 '.' 0.1 ' ' 1
```

```
#RR estimate,lower and upper boundaries of the 95% CI
round(exp(c(m_rq_oral$b,m_rq_oral$ci.lb,m_rq_oral$ci.ub)),digits=2)
```

```
## [1] 1.04 1.12 0.91 1.00 1.19 1.26
```

Checking whether the research question is a significant moderator.

```
capture.output(rma(yi,vi,mods=cbind(rq_rel ,rq_unrel),data=data_op))[c(8,10,11,13,14)]
```

```
## [1] "R^2 (amount of heterogeneity accounted for):            0.00%"
## [2] "Test for Residual Heterogeneity: "
## [3] "QE(df = 88) = 680.1428, p-val < .0001"
## [4] "Test of Moderators (coefficient(s) 2:3): "
## [5] "QM(df = 2) = 0.7643, p-val = 0.6824"
```
